# Supplementary material for: Canonical autophagy remains inactive in induced pluripotent stem cells and neuronal progenitor cells following DNA damage induced by BPDE or etoposide
Source: Sci Rep. 2026 Jun 8;16:18028. doi: 10.1038/s41598-026-54127-6 (PMC13254337; doi:10.1038/s41598-026-54127-6)
Supplement: Supplementary file 1 — Supplementary Material 1 [file 41598_2026_54127_MOESM1_ESM.pdf]

Figure 1:

iPS11

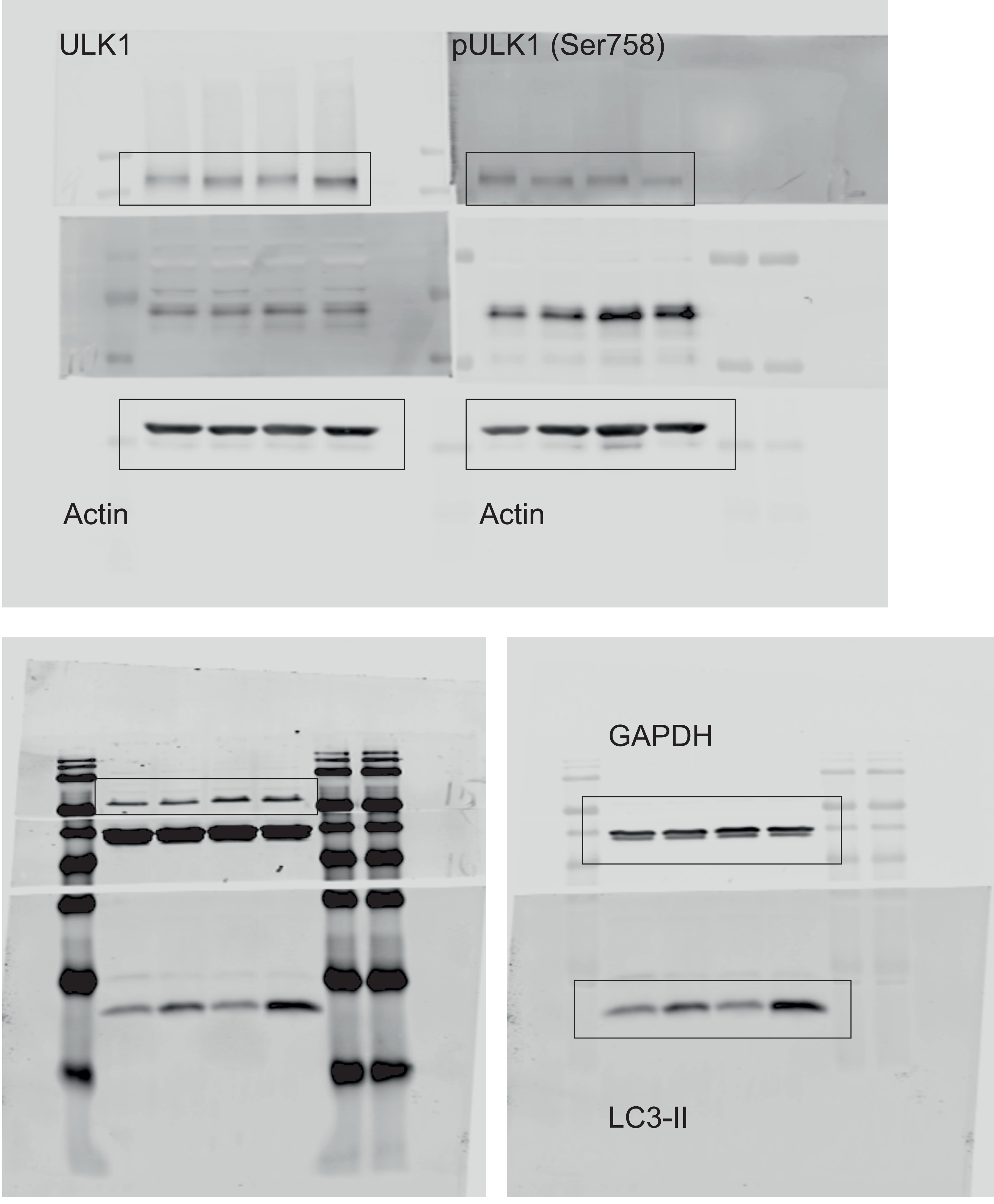

p62

Figure 1:

iPS12

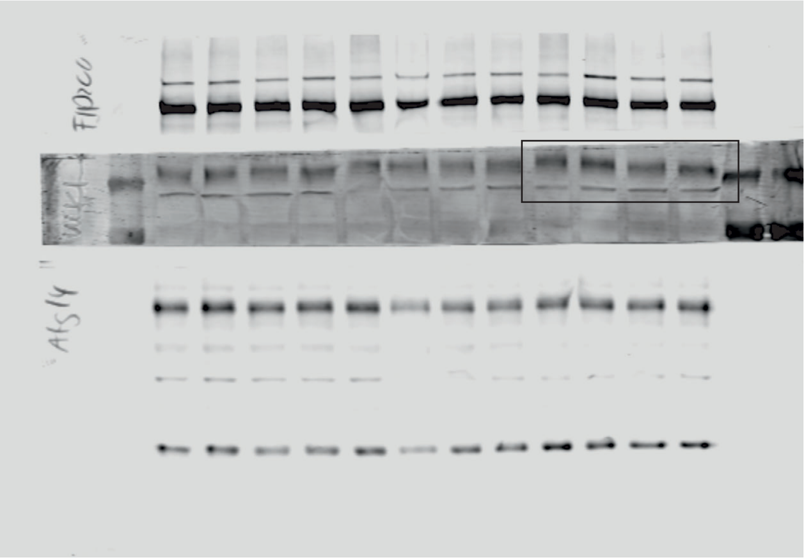

ULK1

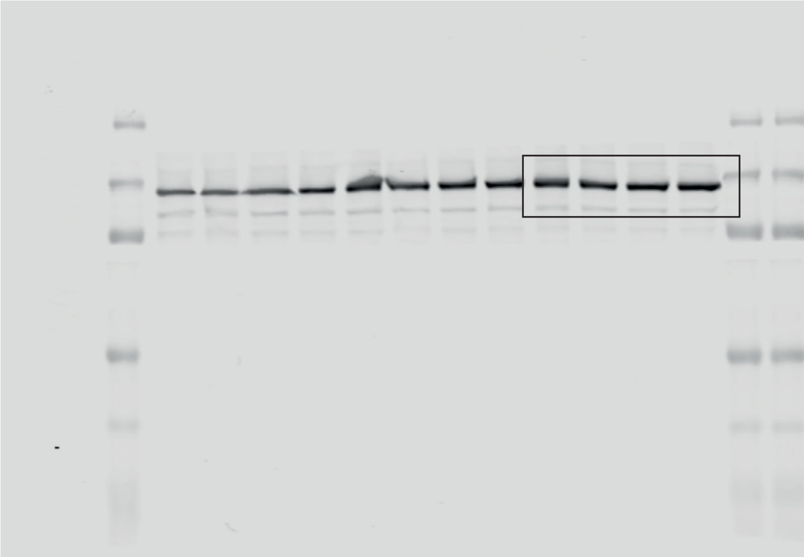

Vinculin

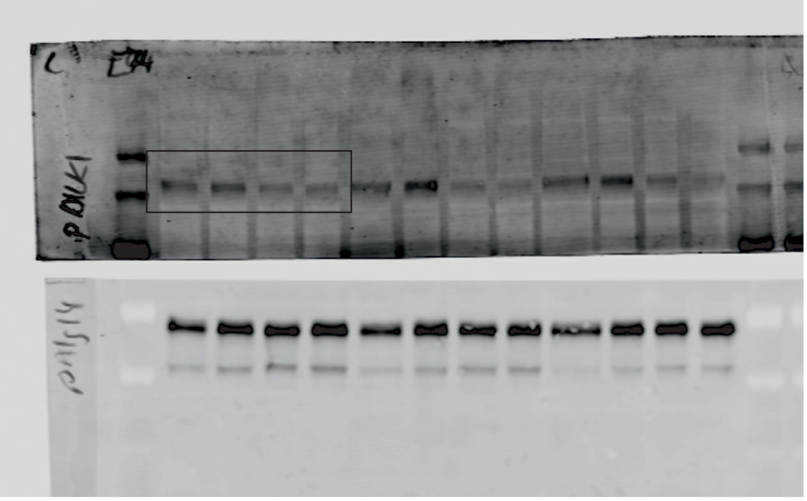

pULK1 (Ser 758)

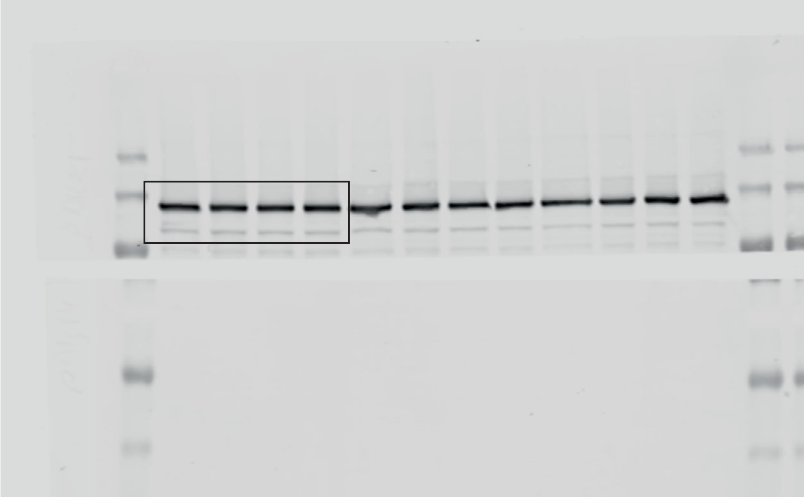

Vinculin

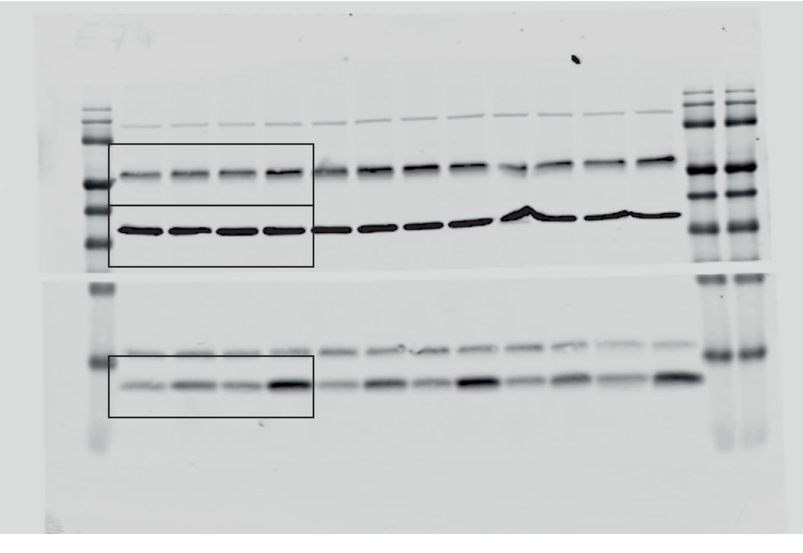

p62

GAPDH

LC3-II

Figure 1:

niPS11

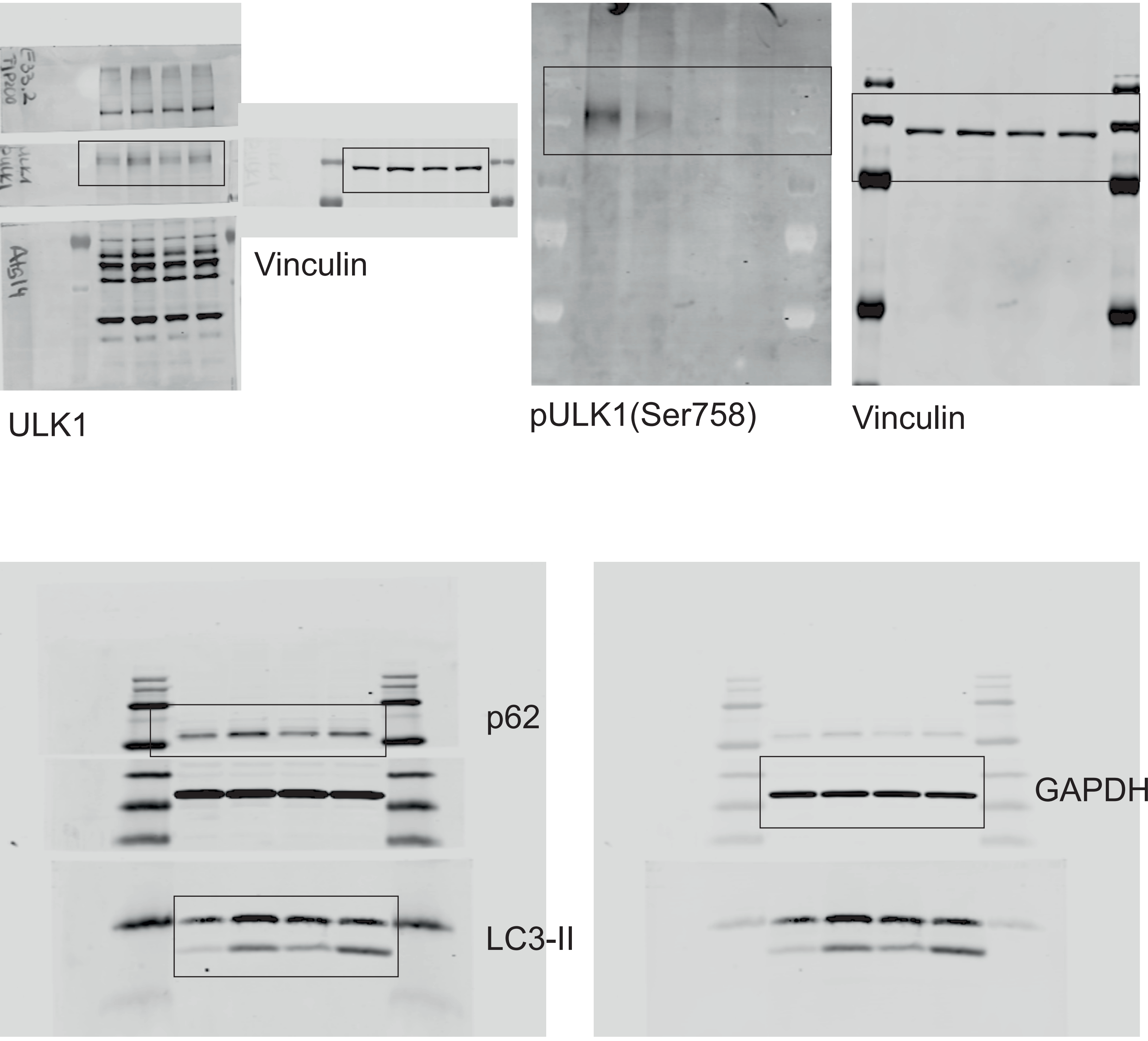

Figure 1:

niPS12

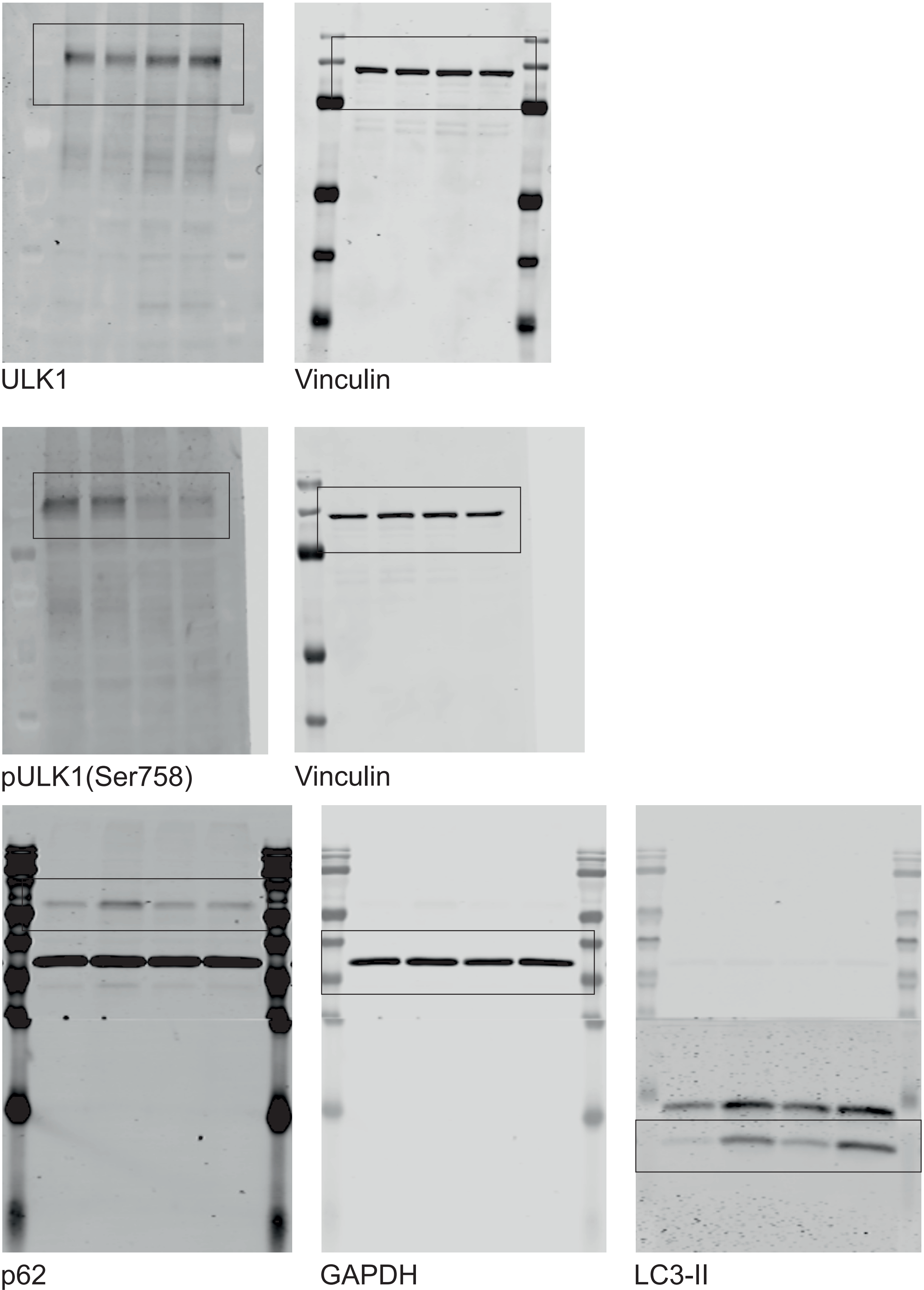

Figure 3:

iPS11; Etoposide

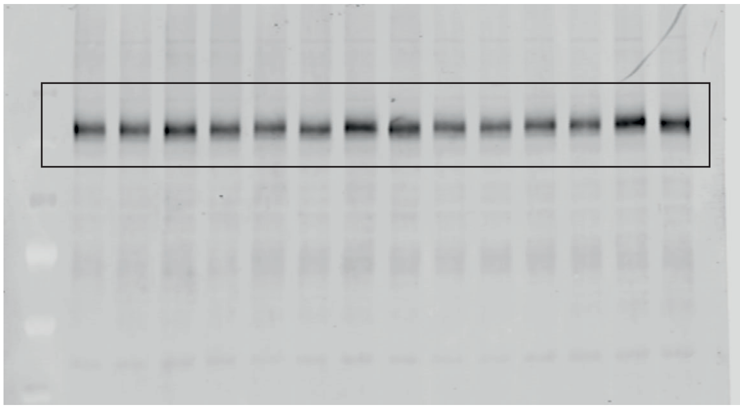

ULK1

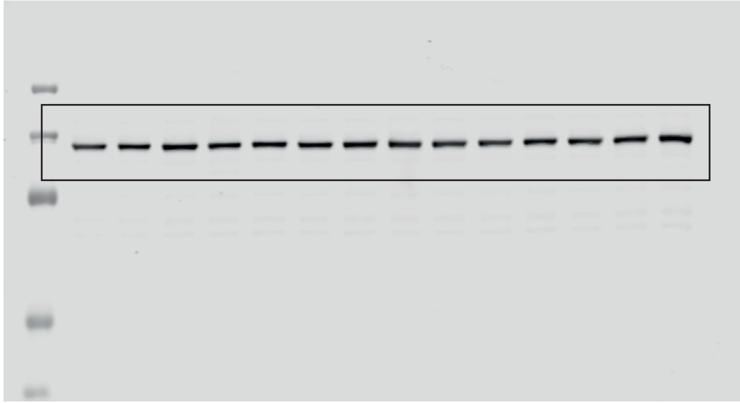

Vinculin

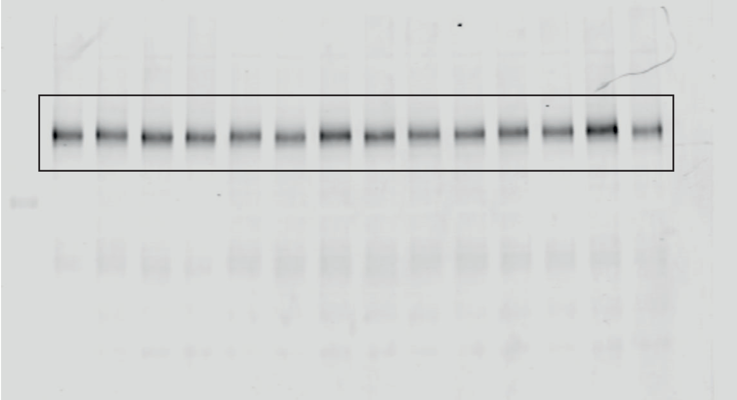

pULK1 (Ser758)

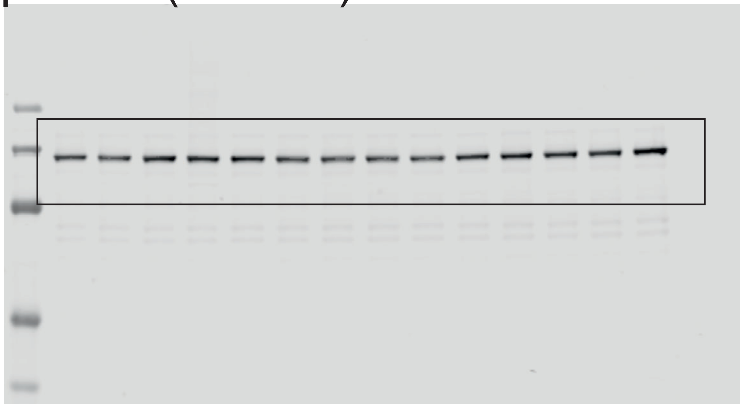

Vinculin

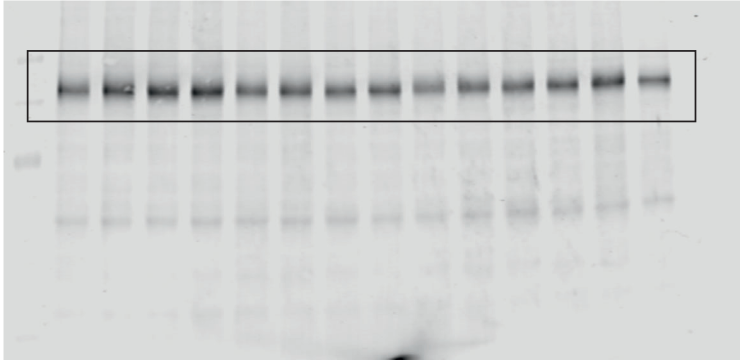

pULK1 (Ser638)

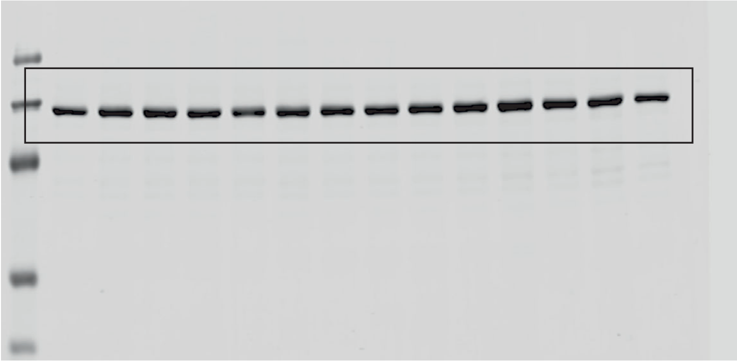

Vinculin

Figure 3:

niPS11; Etoposide

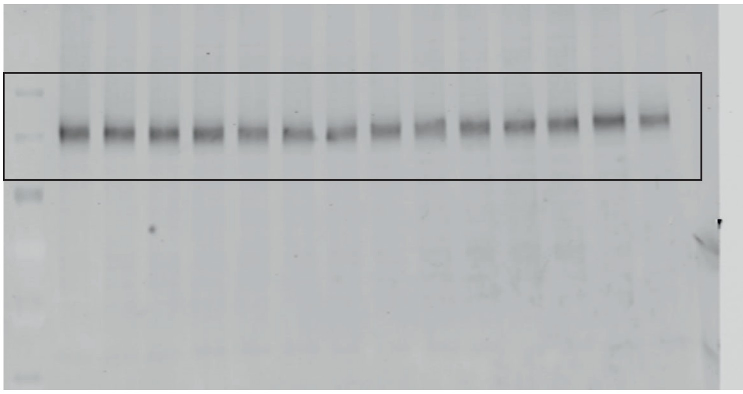

ULK1

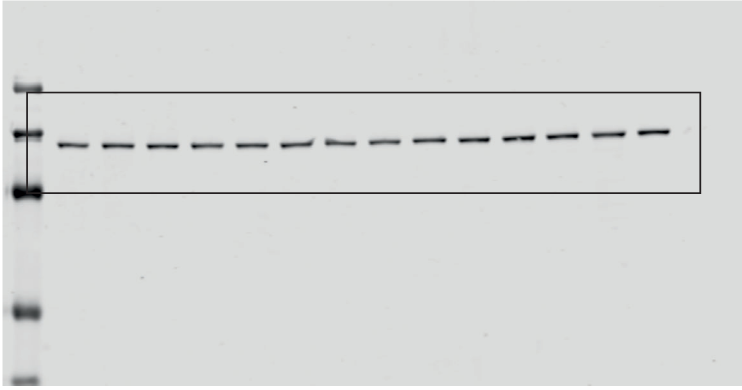

Vinculin

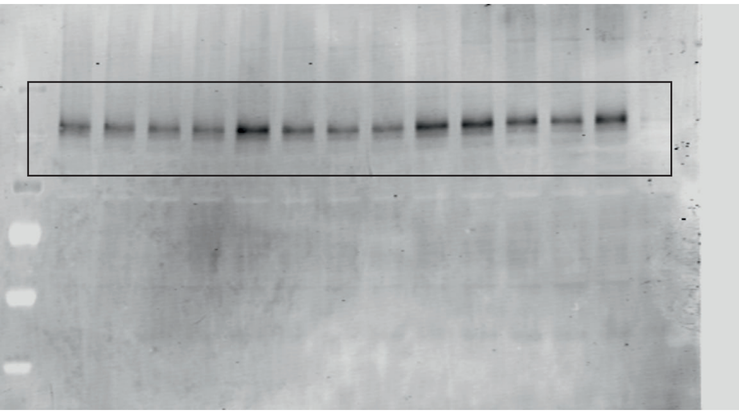

pULK1 (Ser758)

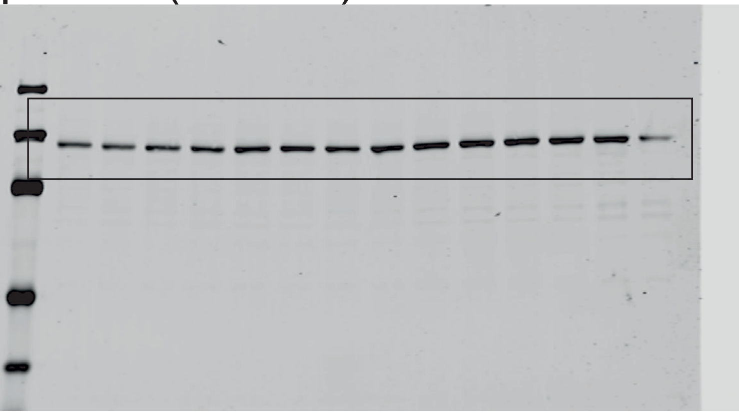

Vinculin

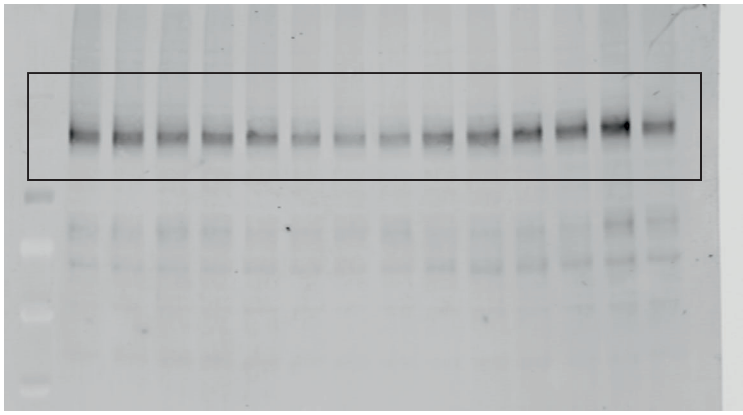

pULK1 (Ser638)

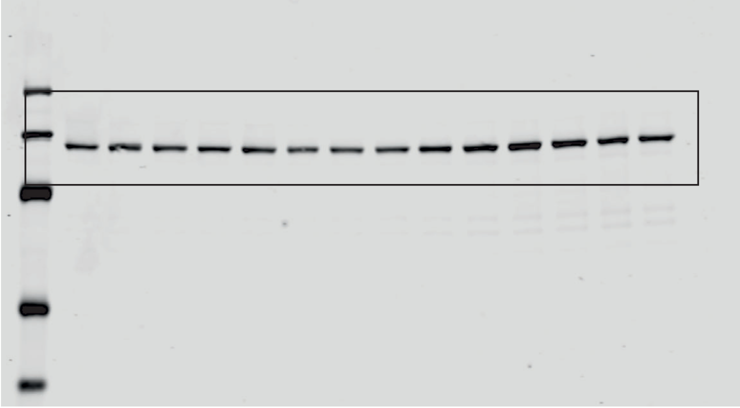

Vinculin

Figure 3:

HCT116; Etoposide

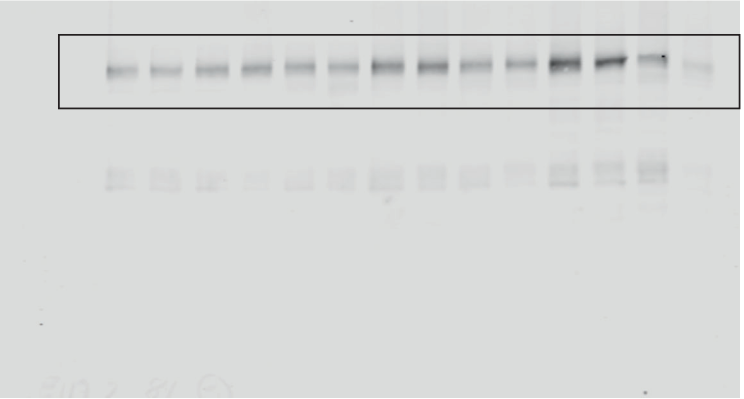

ULK1

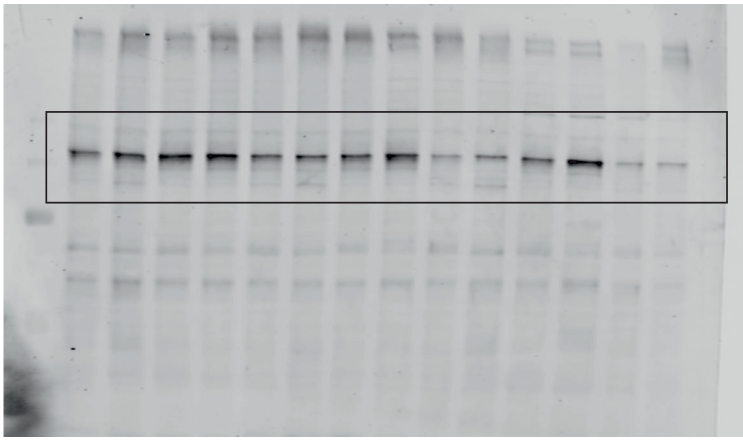

pULK1 (Ser638)

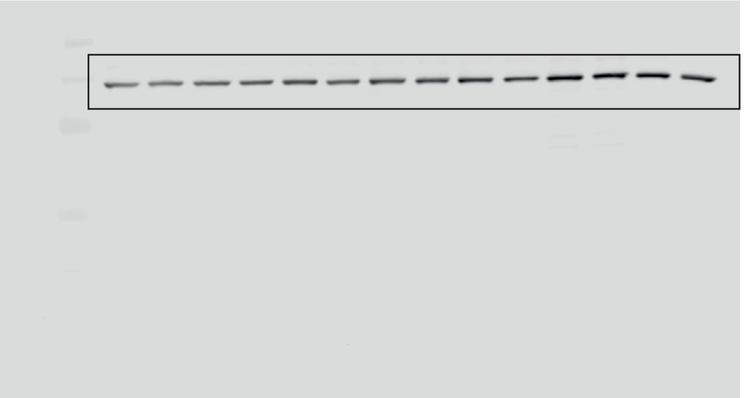

Vinculin

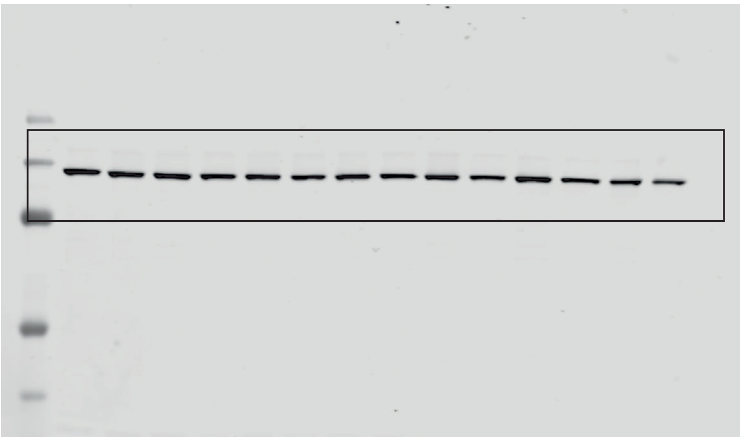

Vinculin

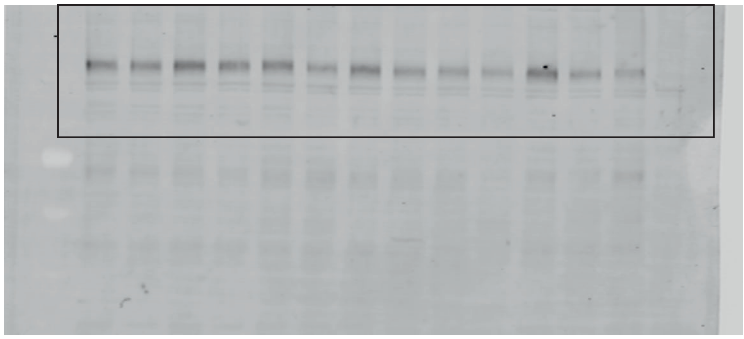

pULK1 (Ser758)

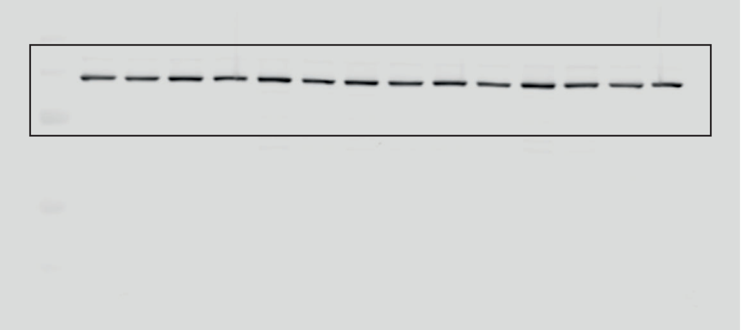

Vinculin

Figure 4:

iPS11; BPDE

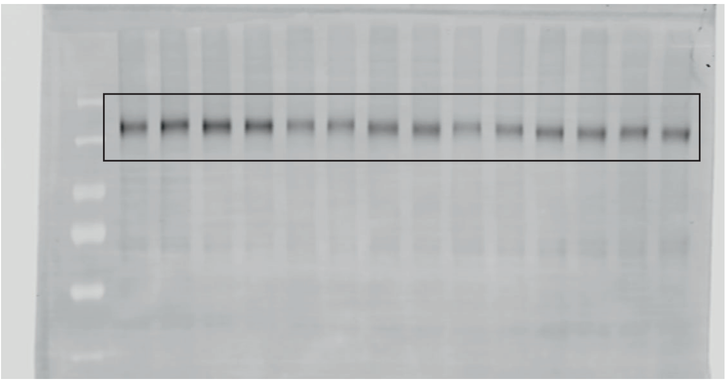

ULK1

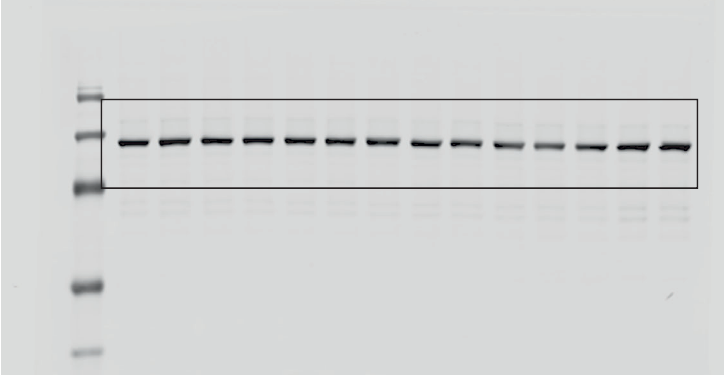

Vinculin

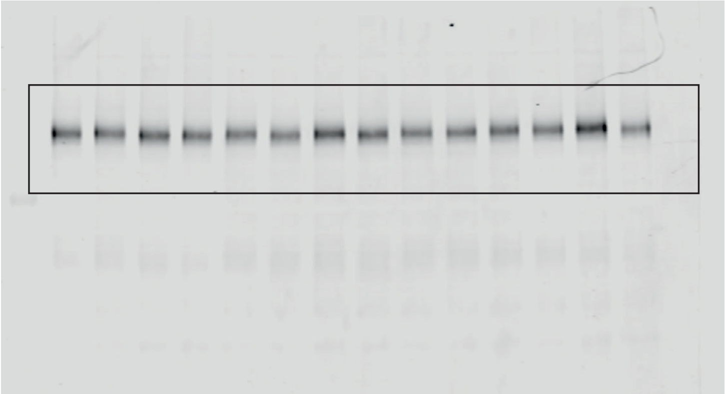

pULK1 (Ser758)

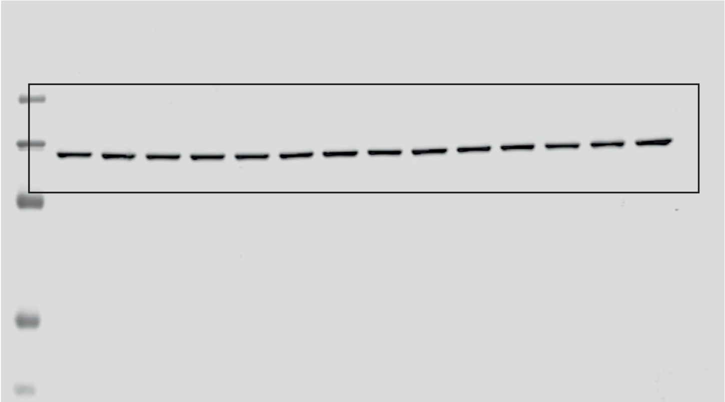

Vinculin

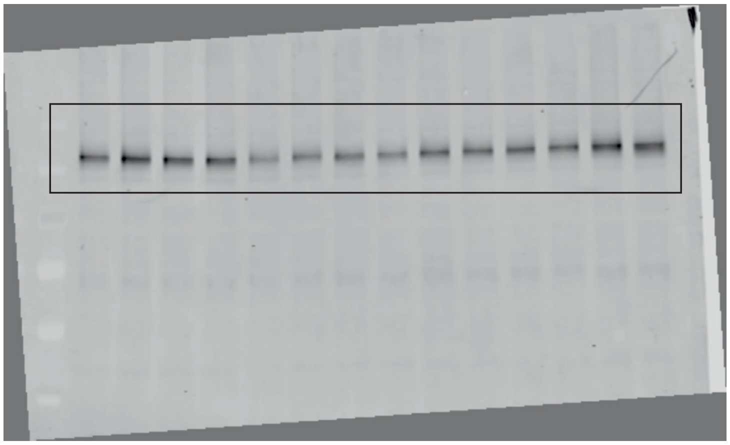

pULK1 (Ser638)

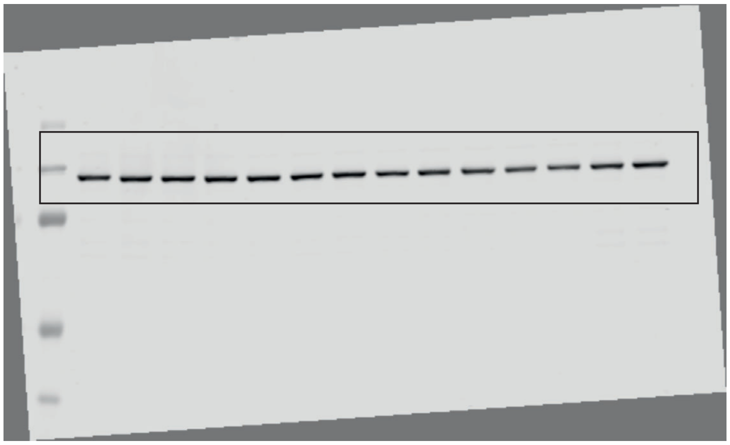

Vinculin

Figure 4:

niPS11; BPDE

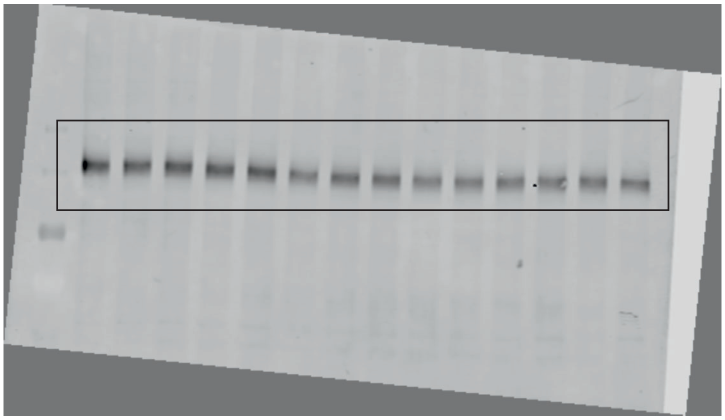

ULK1

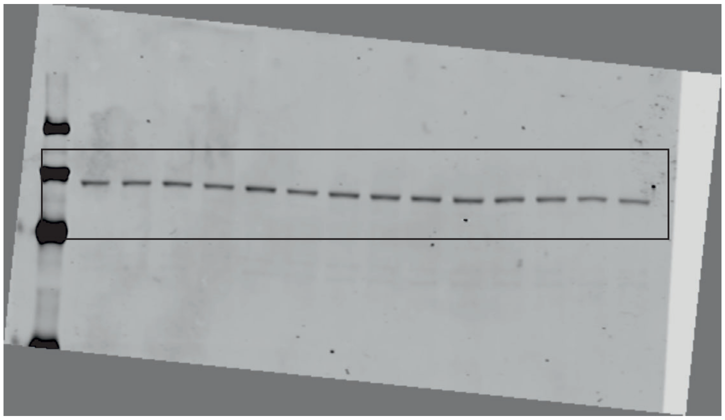

Vinculin

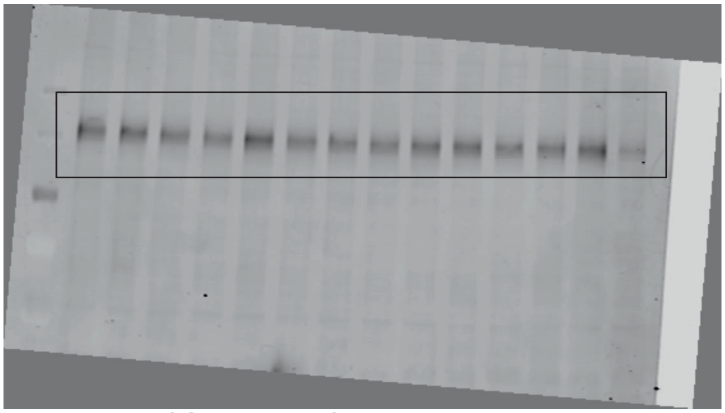

pULK1 (Ser758)

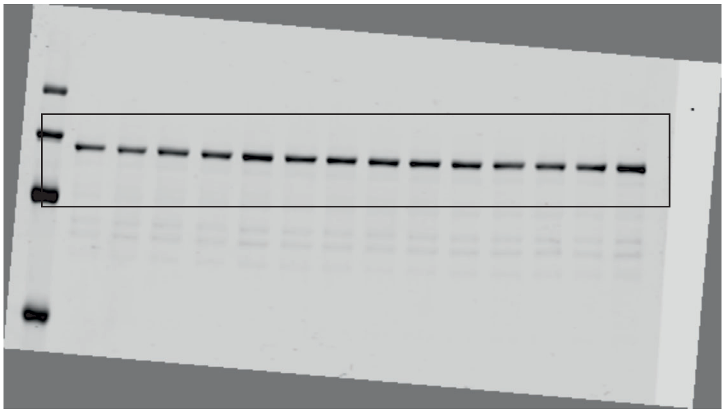

Vinculin

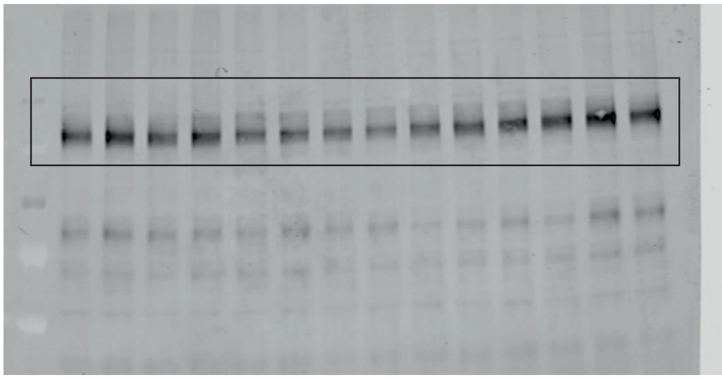

pULK1 (Ser638)

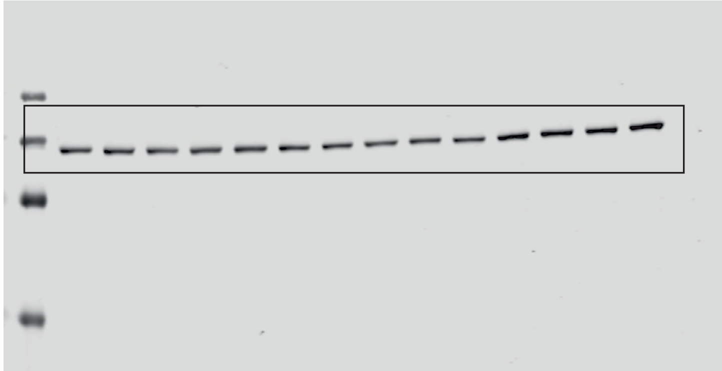

Vinculin

Figure 4:

HCT116; BPDE

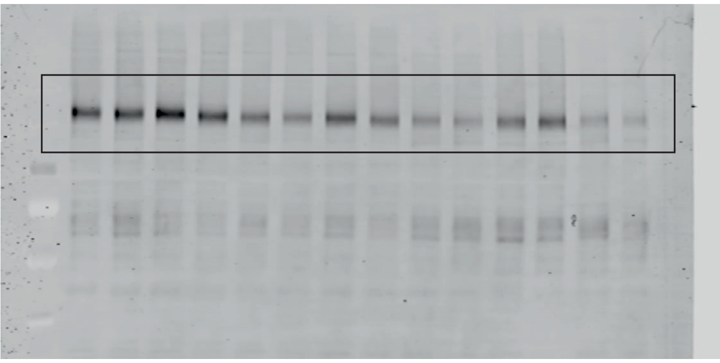

ULK1

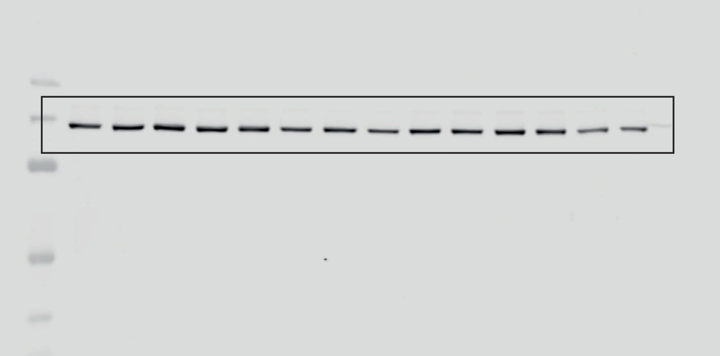

Vinculin

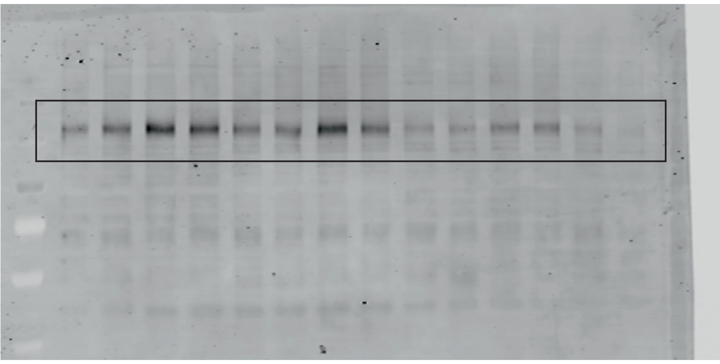

pULK1 (Ser758)

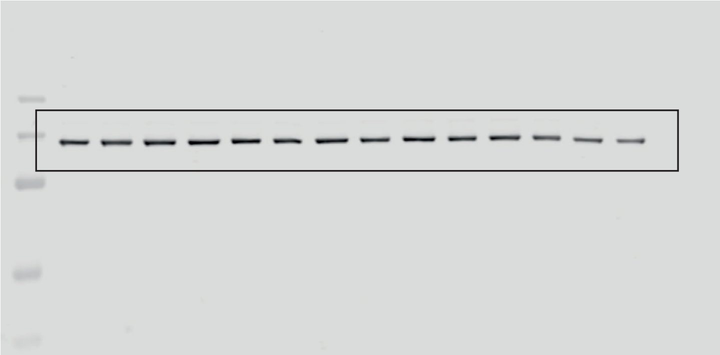

Vinculin

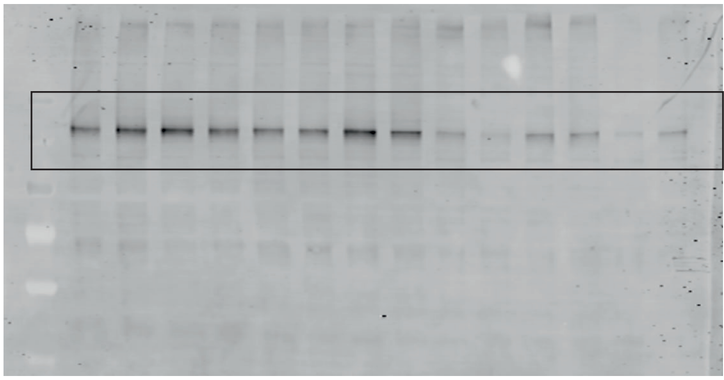

pULK1 (Ser638)

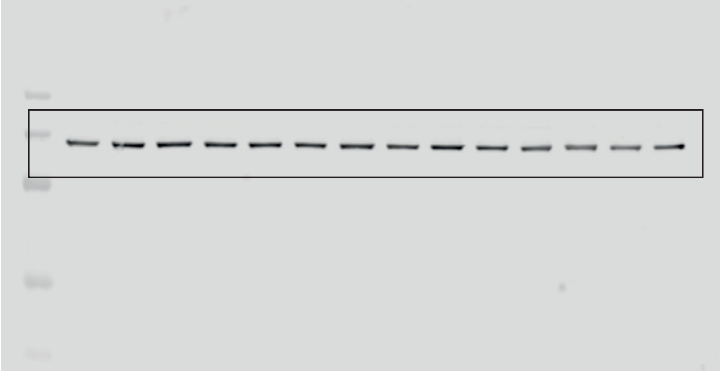

Vinculin

Figure 5:

iPS11; Etoposide

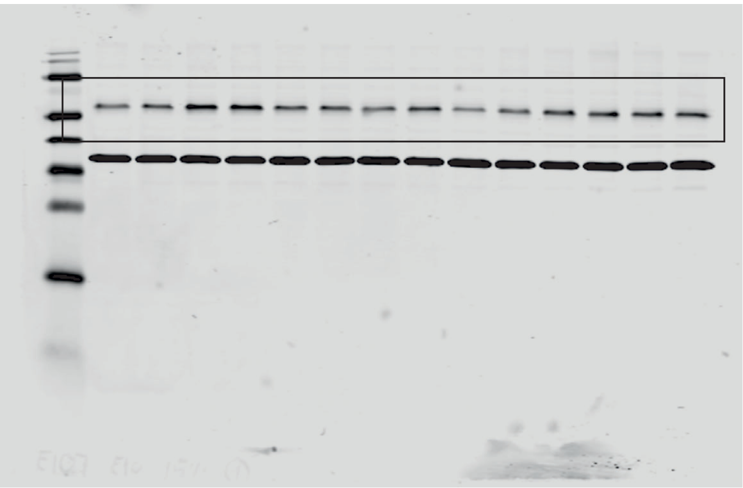

p62

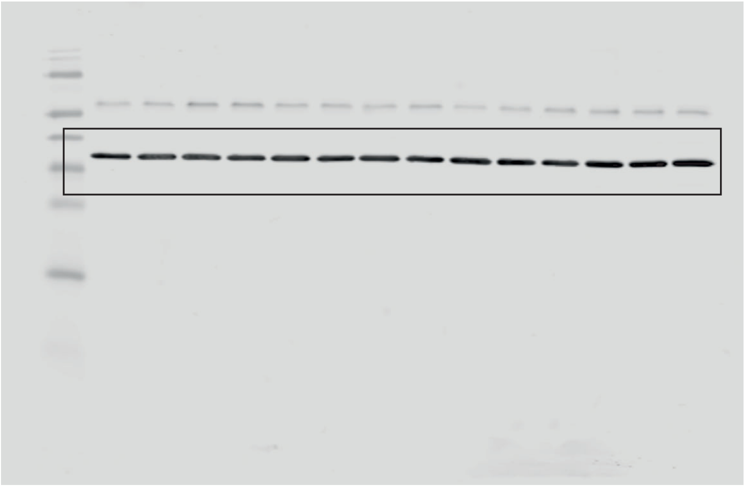

GAPDH

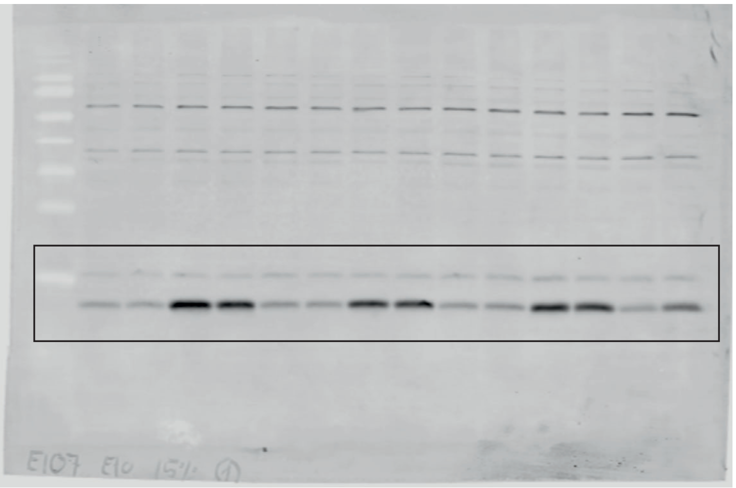

LC3

iPS11; BPDE

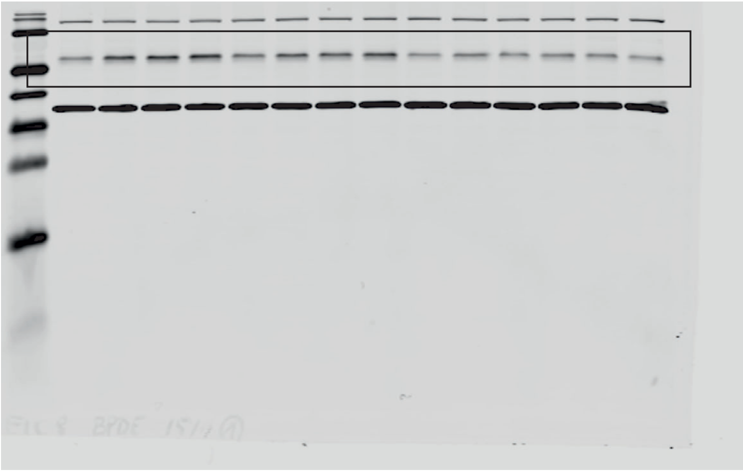

p62

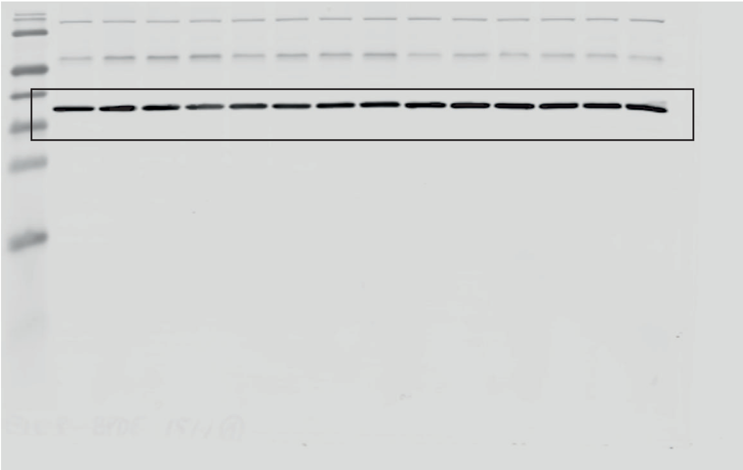

GAPDH

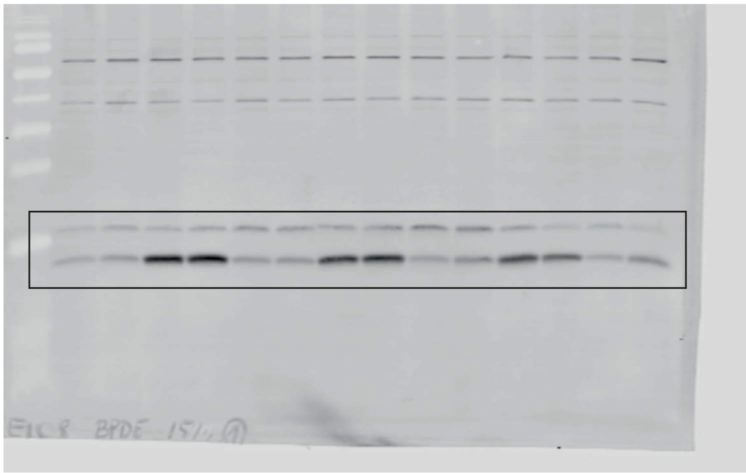

LC3

Figure 5:

niPS11; Etoposide

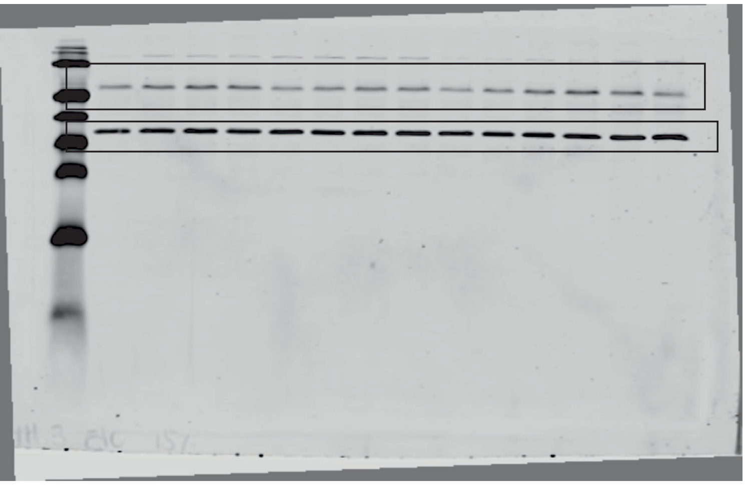

p62
GAPDH

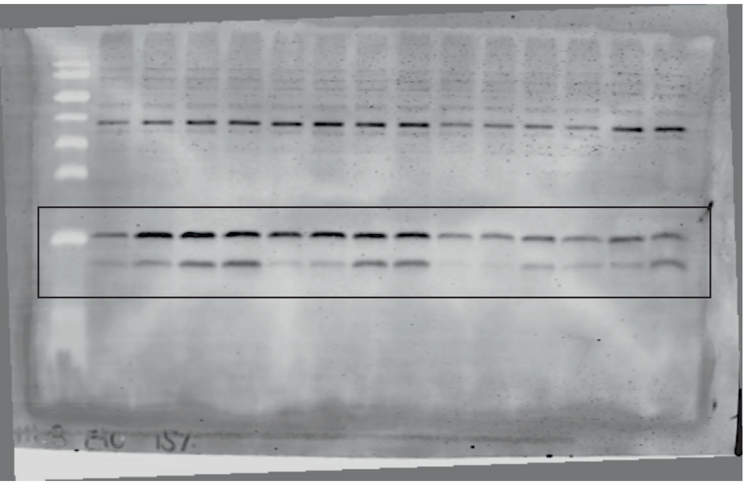

LC3

niPS11; BPDE

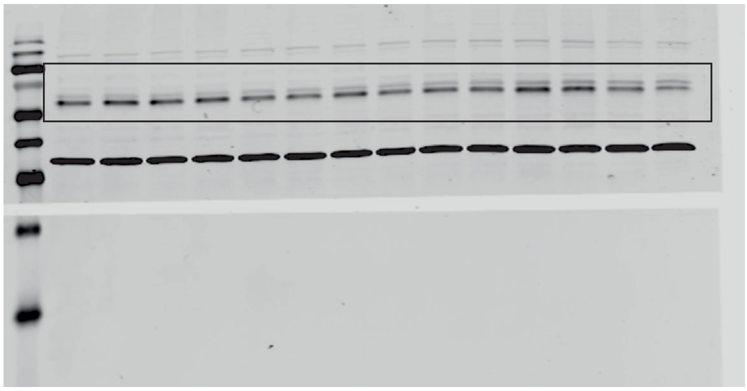

p62

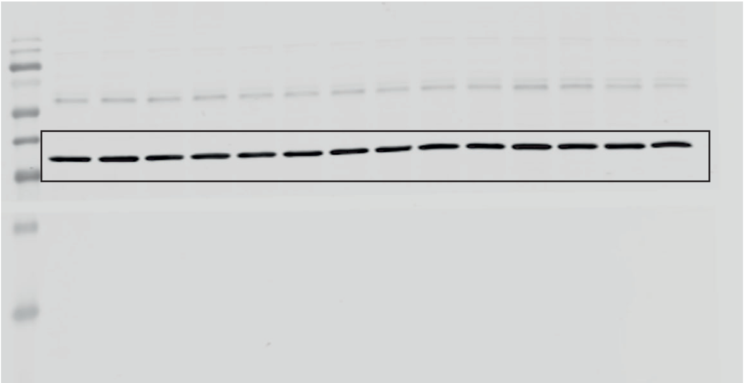

GAPDH

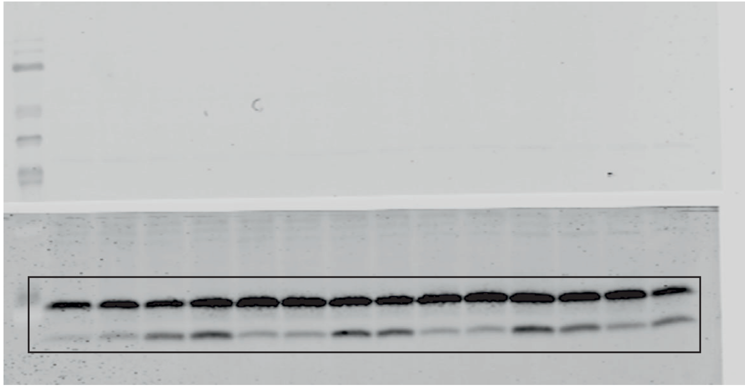

LC3

Figure 5:

HCT116; Etoposide

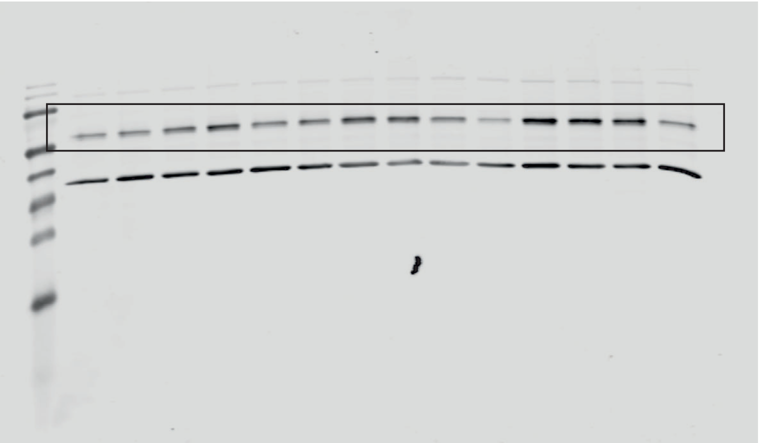

p62

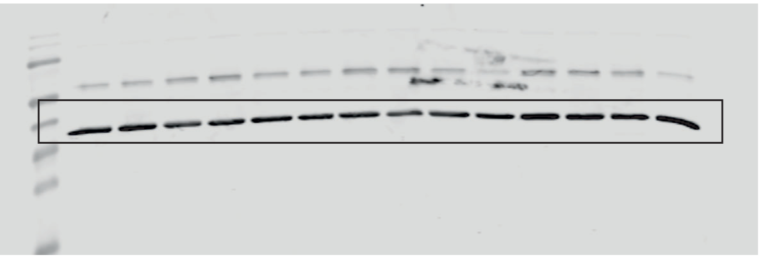

GAPDH

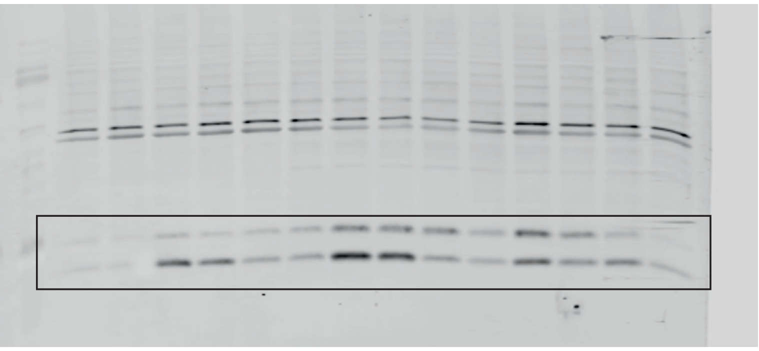

LC3

HCT116; BPDE

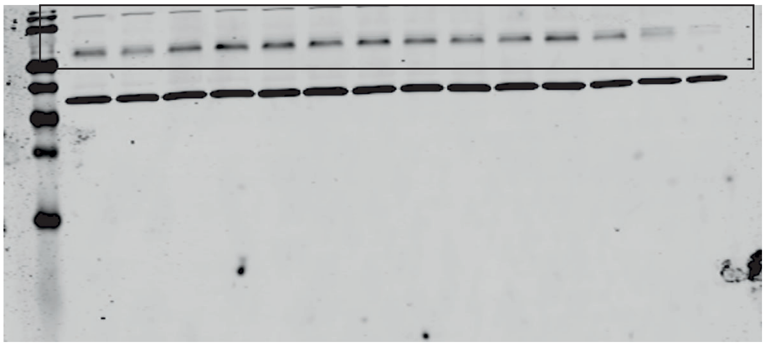

p62

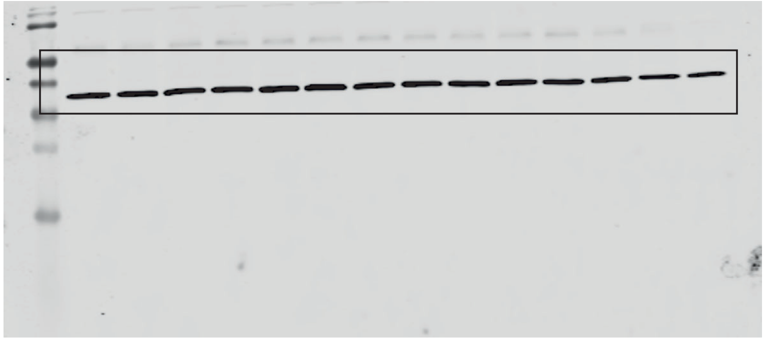

GAPDH

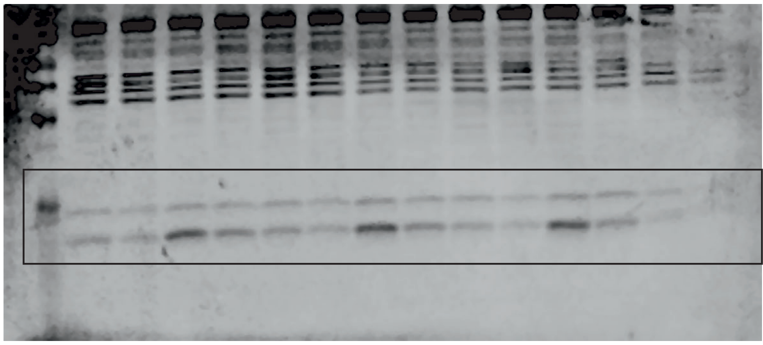

LC3

Figure 7:

niPS11; BPDE

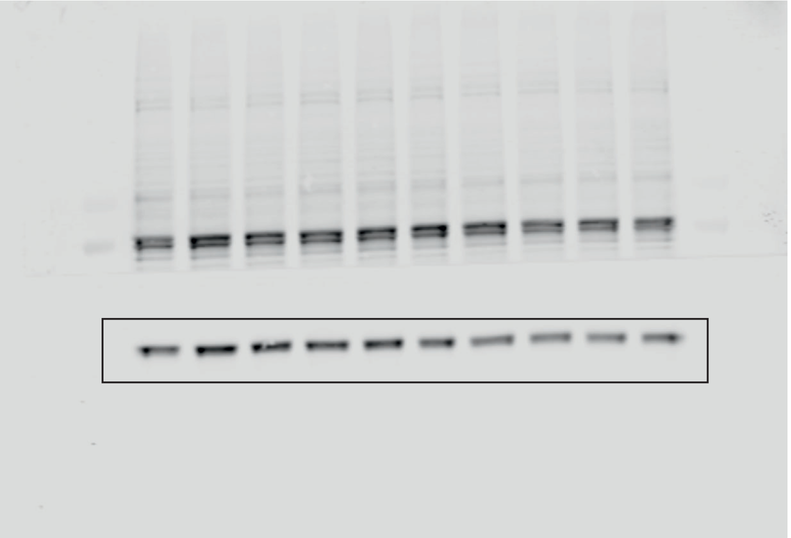

Aurora A

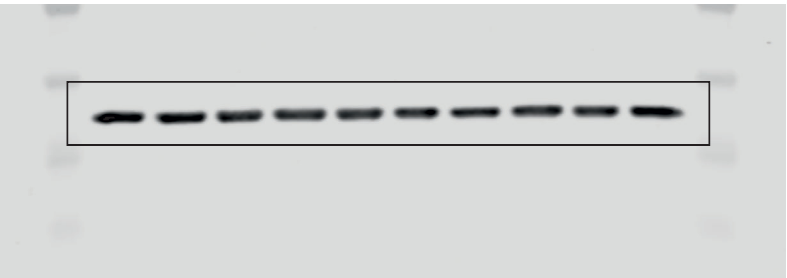

GAPDH

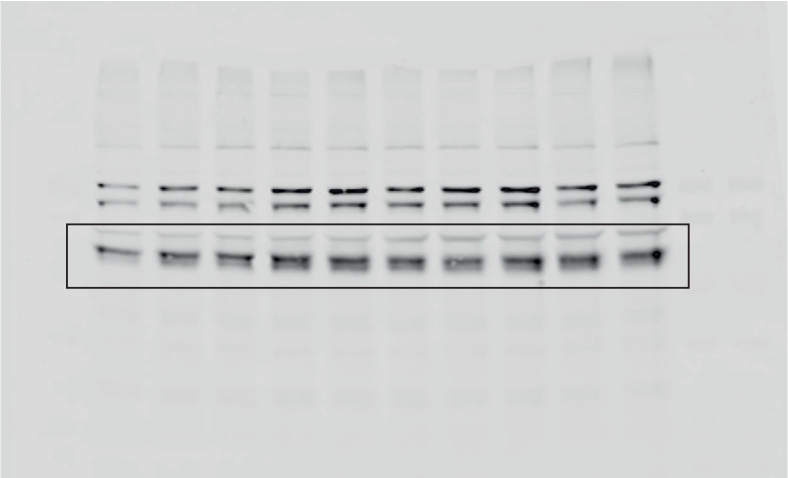

PLK1

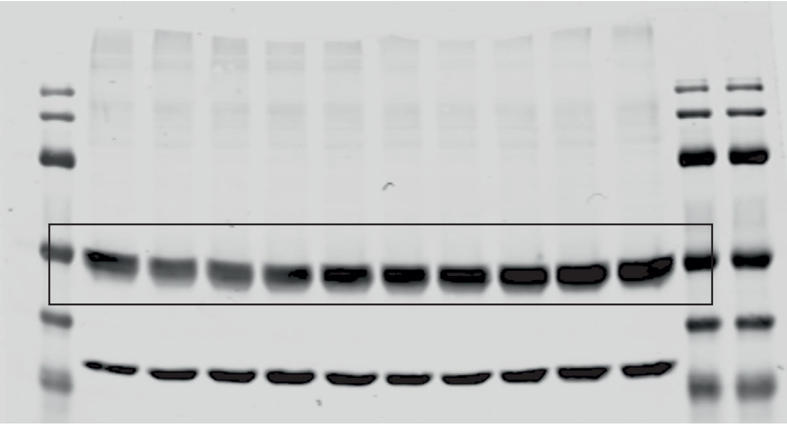

Acetylated Tubulin

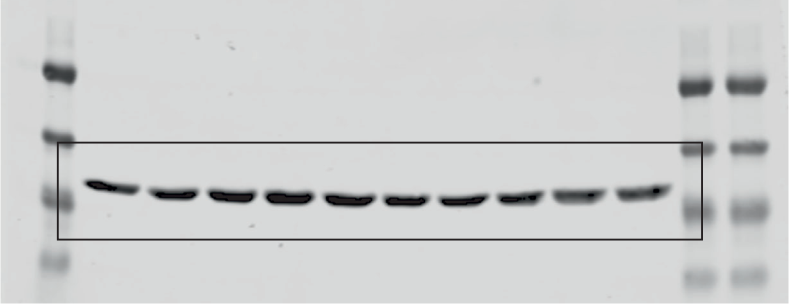

GAPDH

Figure 7:

iPS11; BPDE

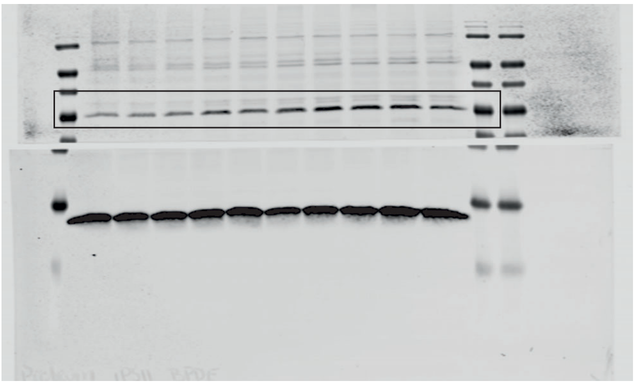

NUCKS1

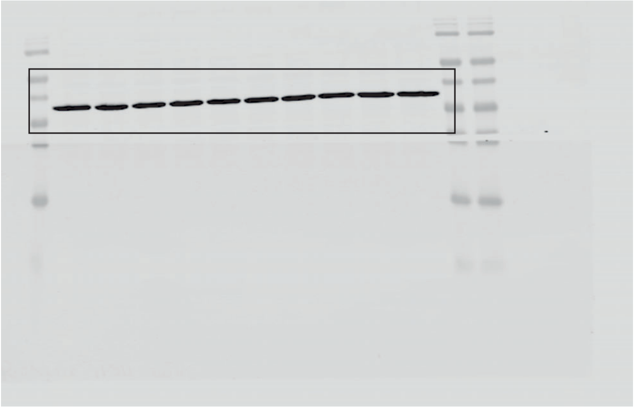

GAPDH

niPS11; Etoposide

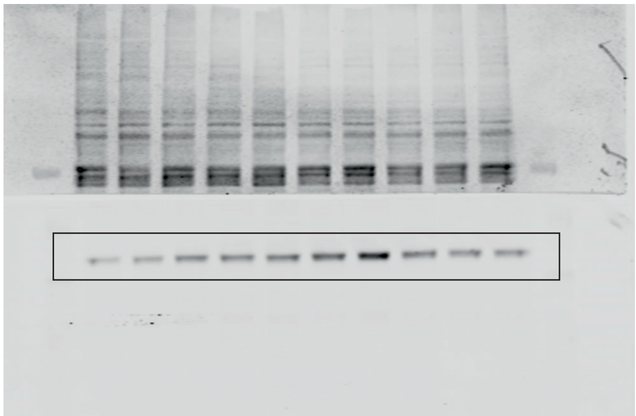

Aurora A

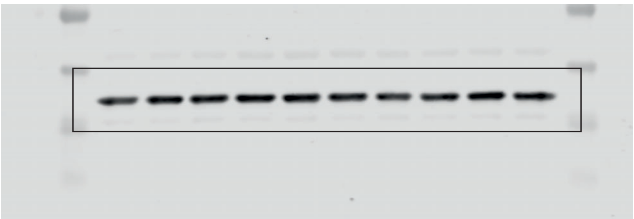

GAPDH

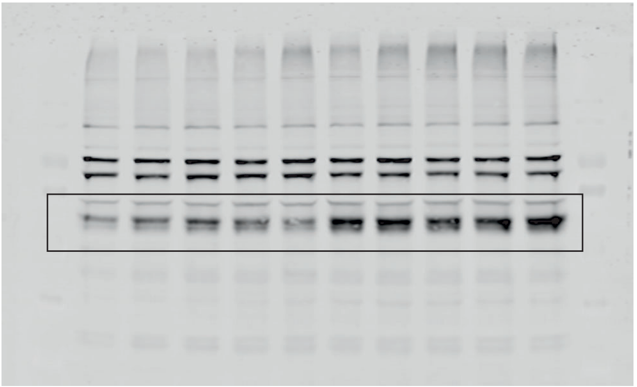

PLK1

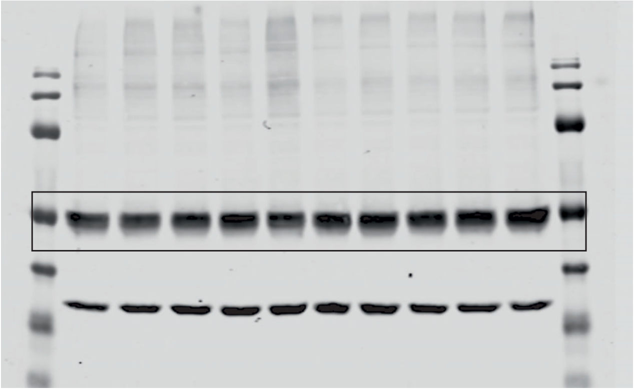

Acetylated Tubulin

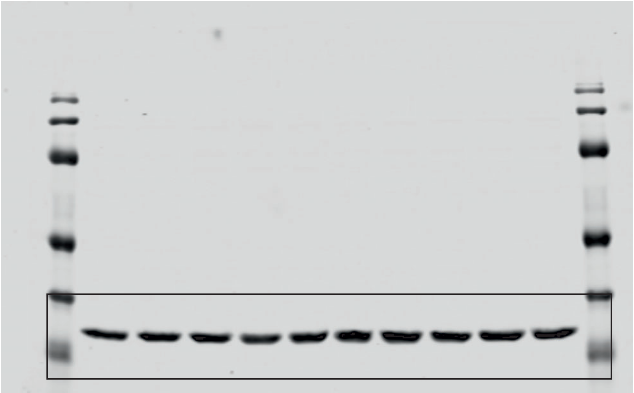

GAPDH

Figure S1:

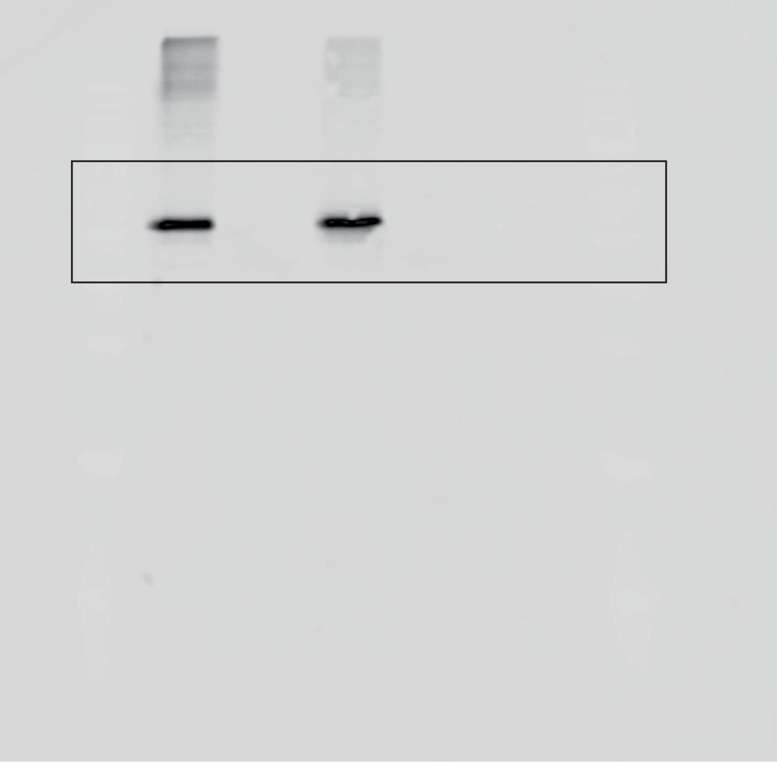

OCT4a

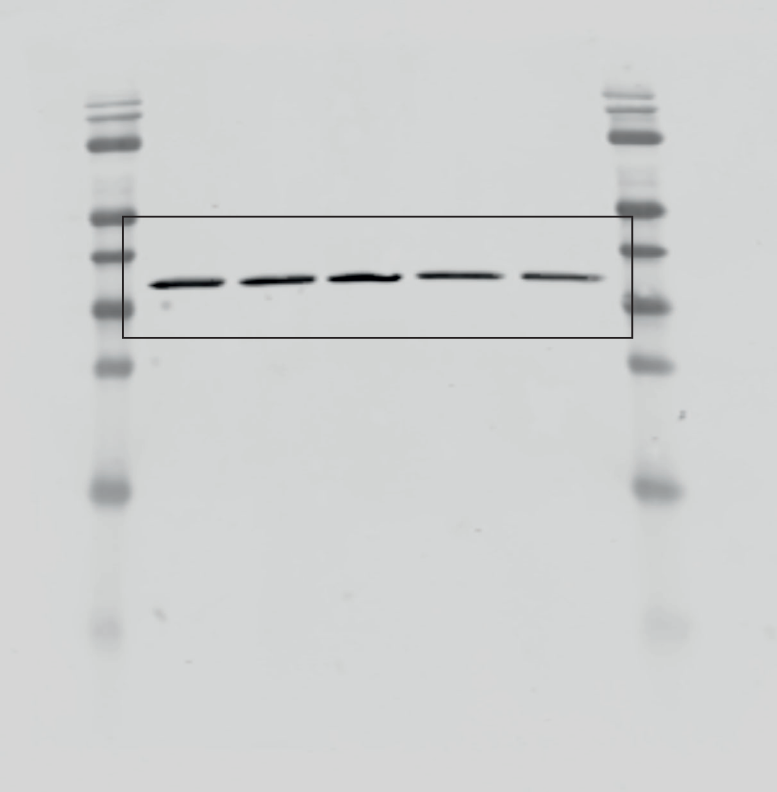

GAPDH

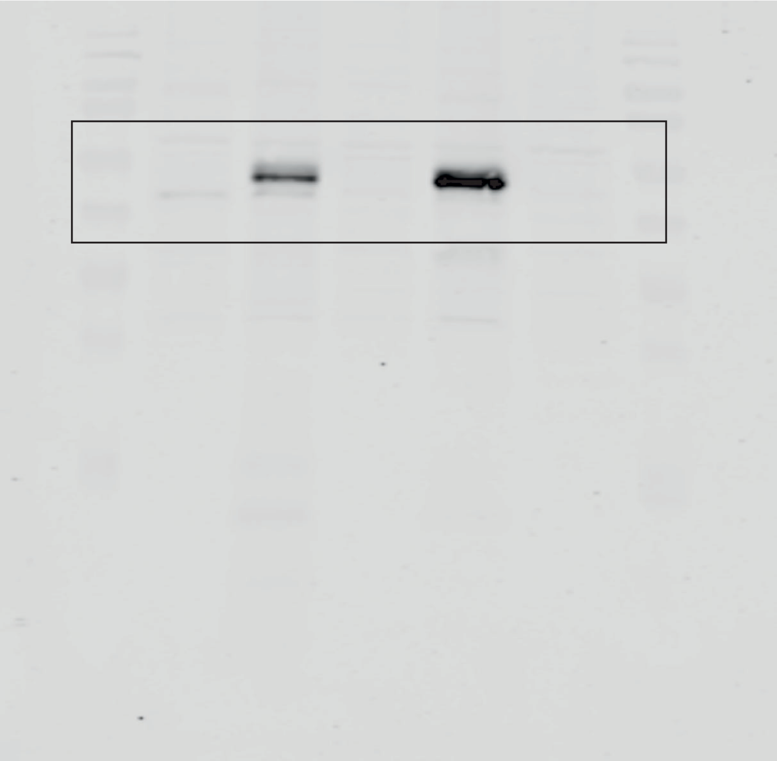

Pax6

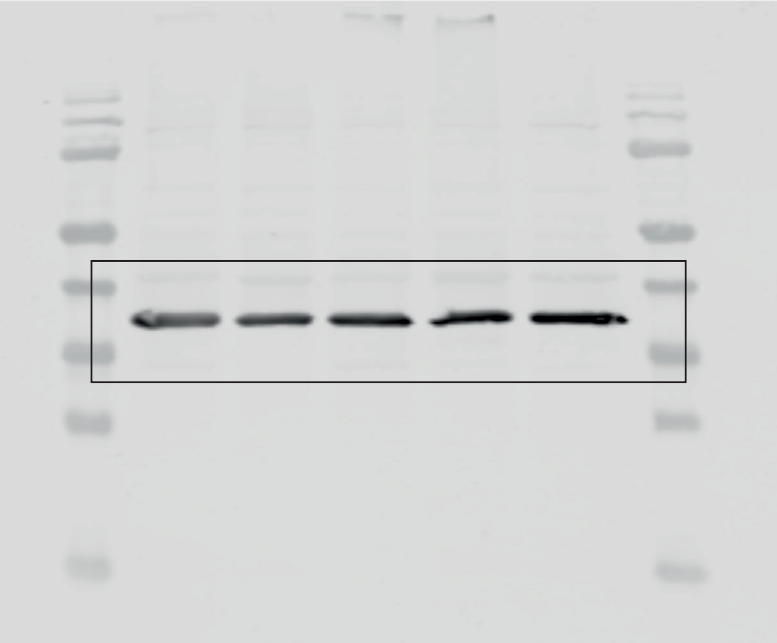

GAPDH

Figure S3:

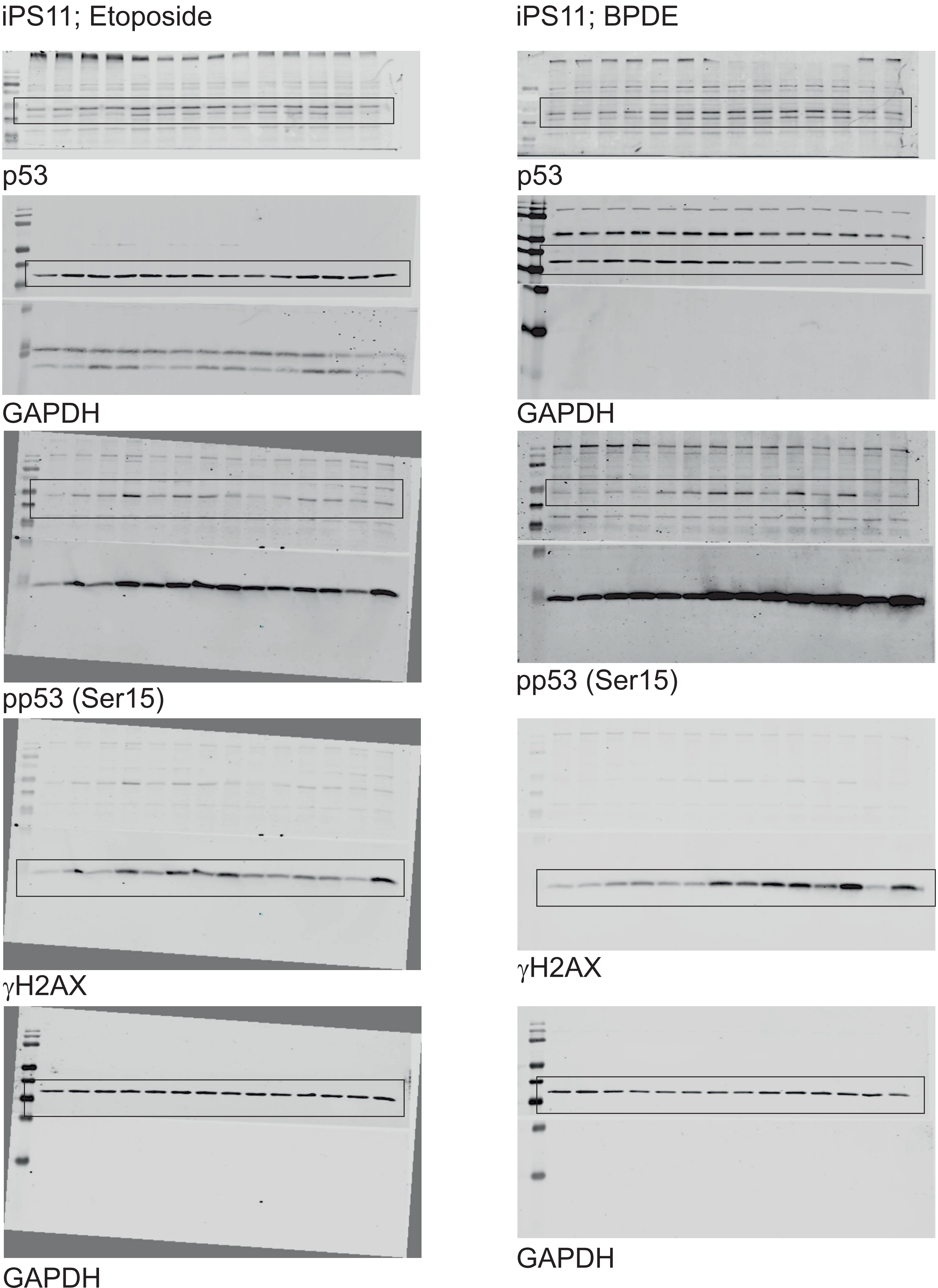
iPS11; Etoposide
iPS11; BPDE
p53
p53
GAPDH
GAPDH
pp53 (Ser15)
pp53 (Ser15)
γH2AX
γH2AX
GAPDH
GAPDH

Figure S3:

niPS11; Etoposide

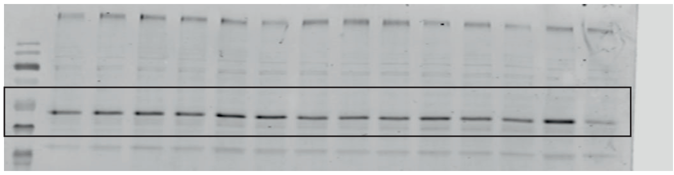

p53

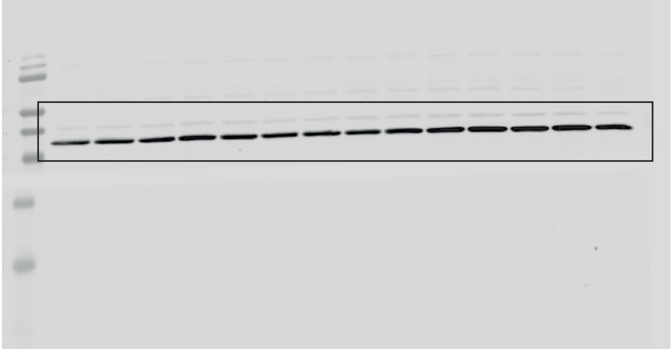

GAPDH

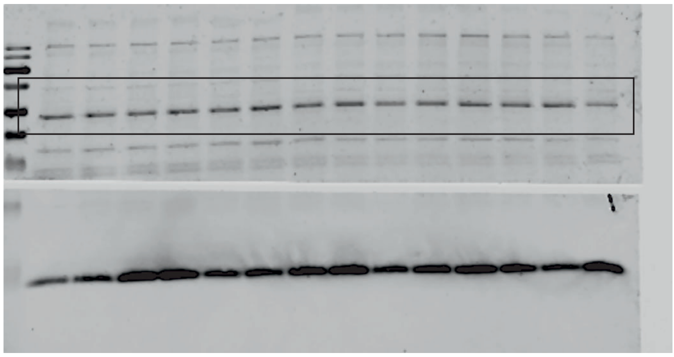

pp53 (Ser15)

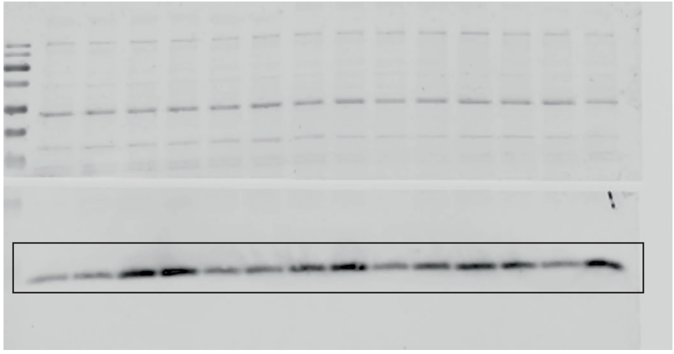

γH2AX

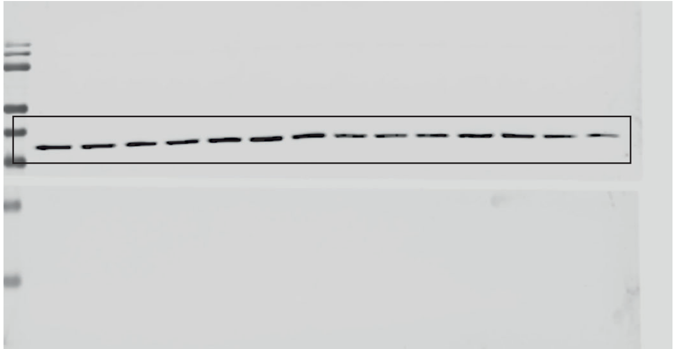

GAPDH

niPS11; BPDE

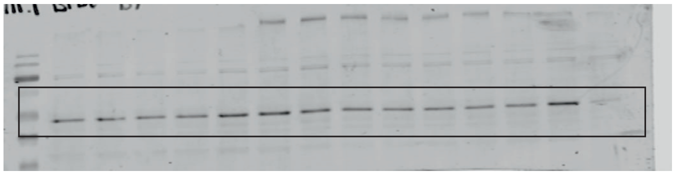

p53

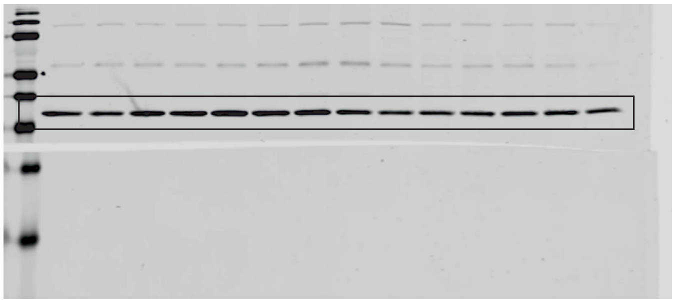

GAPDH

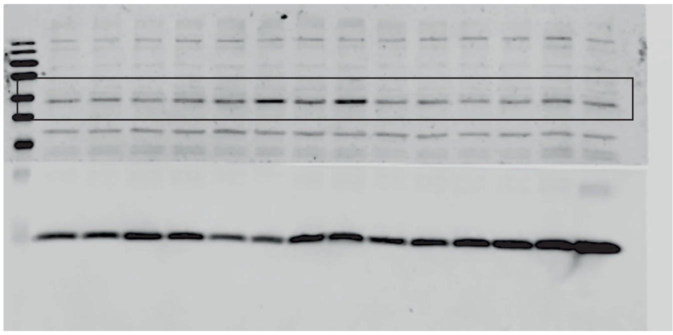

pp53 (Ser15)

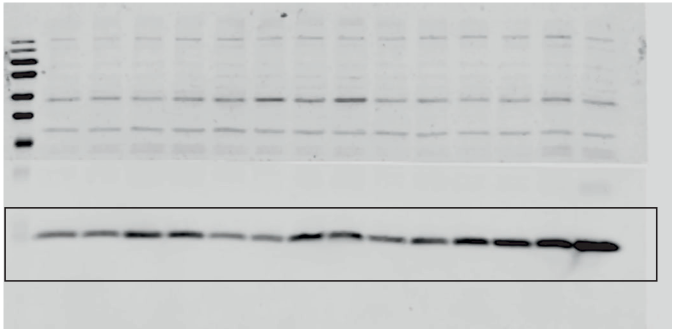

γH2AX

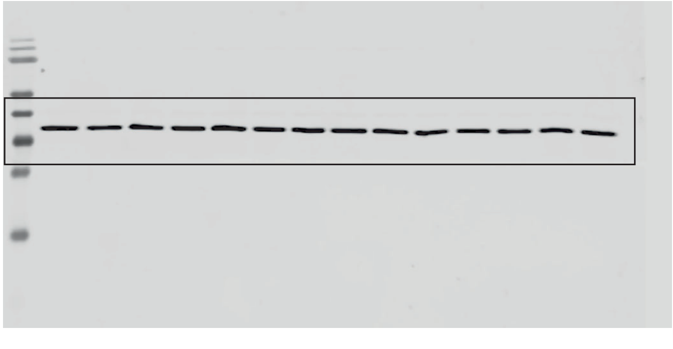

GAPDH

Figure S3:

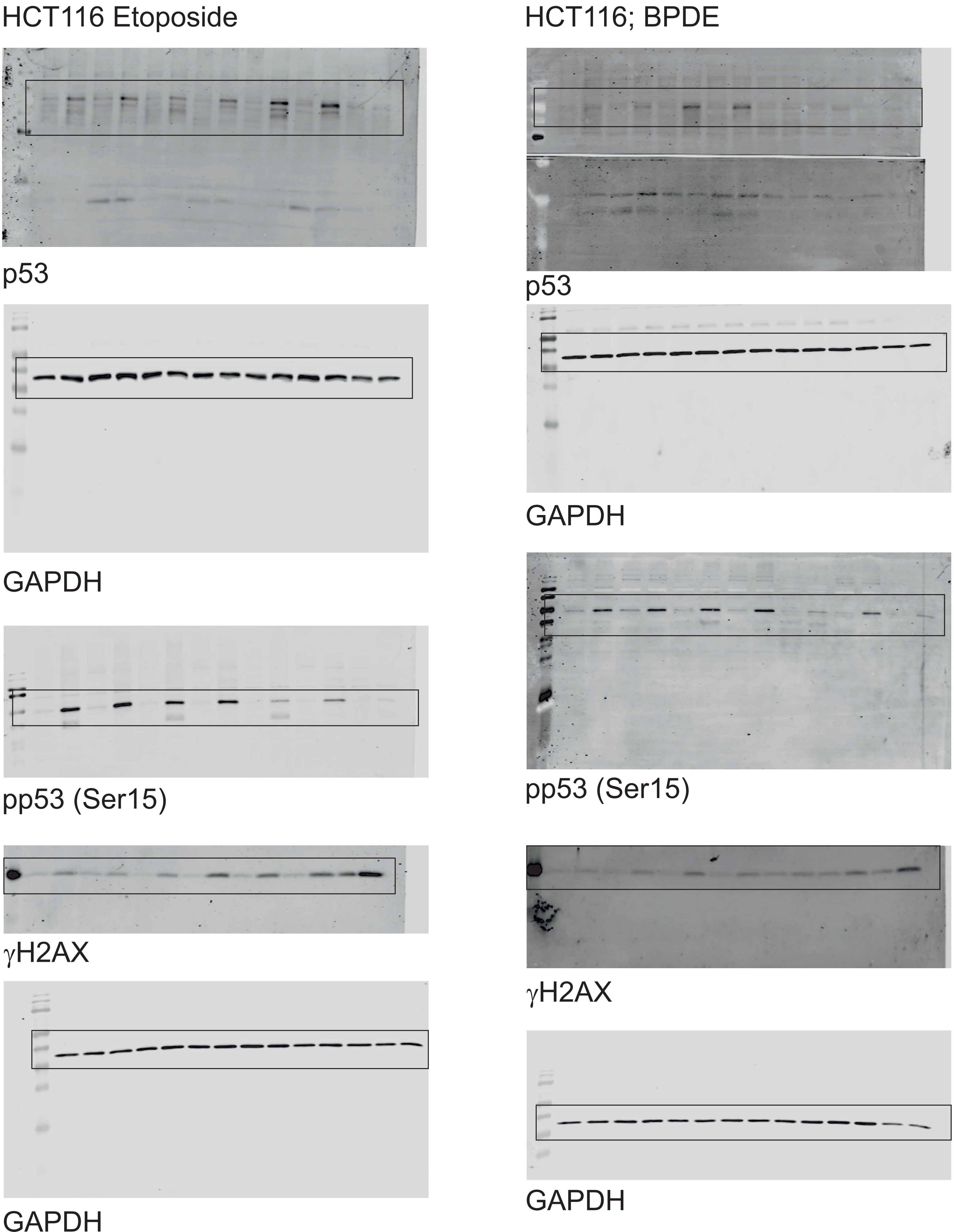

HCT116 Etoposide
HCT116; BPDE
p53
p53
GAPDH
GAPDH
pp53 (Ser15)
pp53 (Ser15)
γH2AX
γH2AX
GAPDH
GAPDH

Figure S3:

iPS11; Etoposide

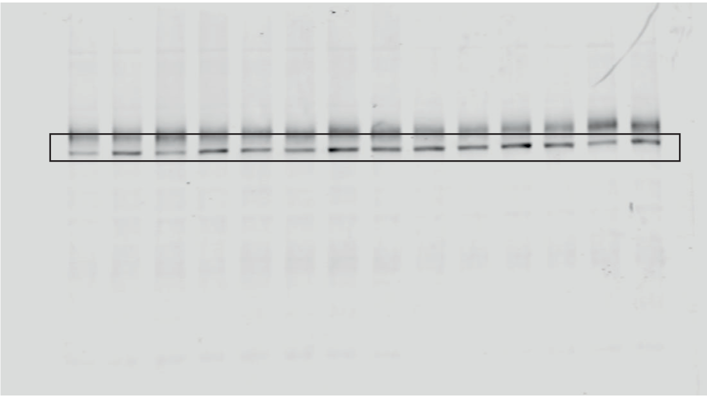

XPC

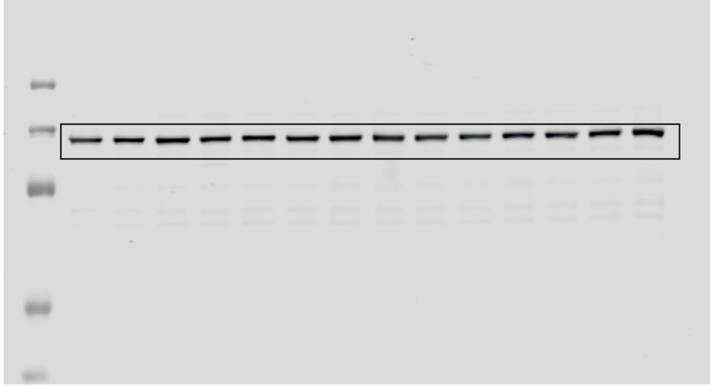

Vinculin

iPS11; BPDE

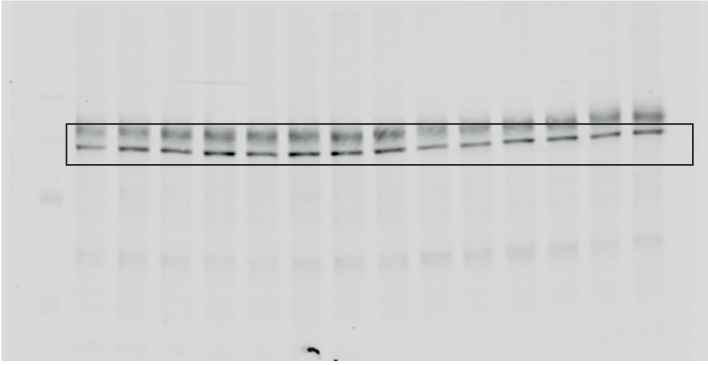

XPC

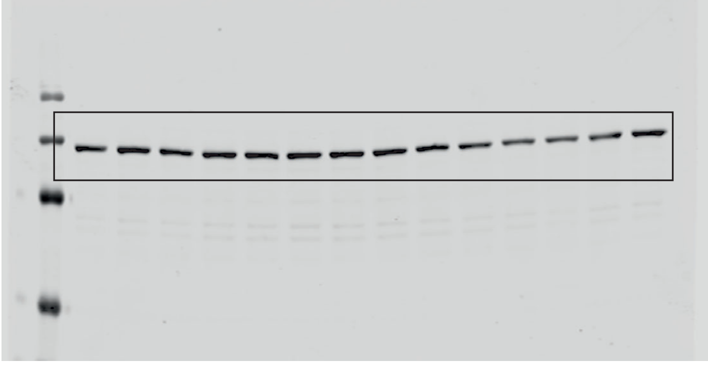

Vinculin

niPS11; Etoposide

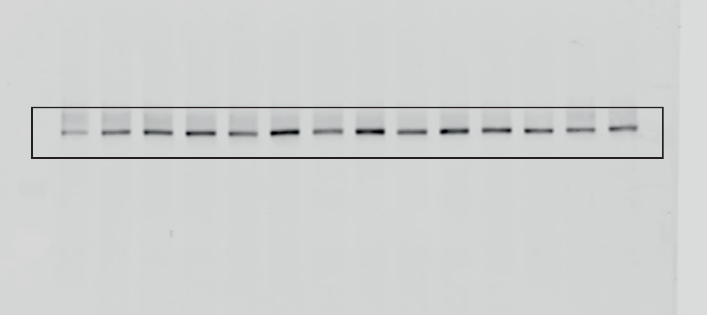

XPC

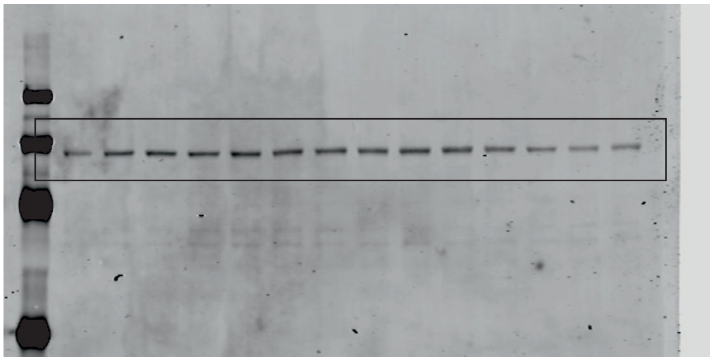

Vinculin

niPS11; BPDE

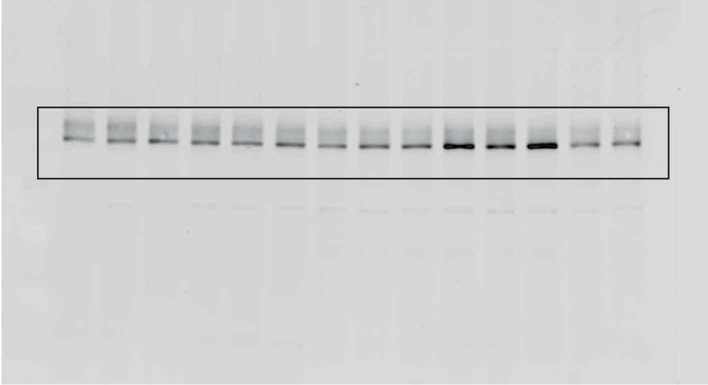

XPC

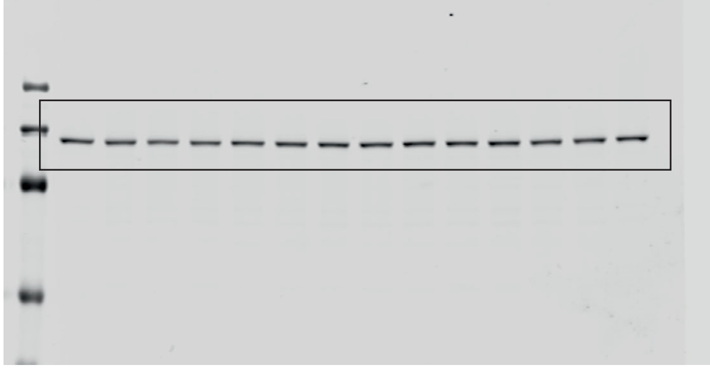

Vinculin

Figure S3:

HCT116; Etoposide

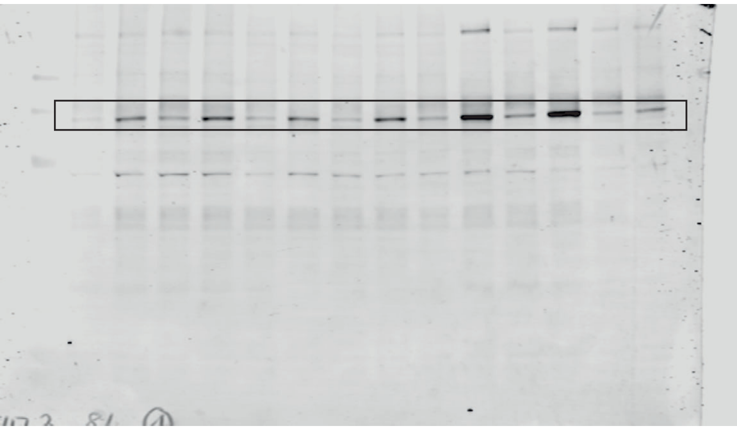

XPC

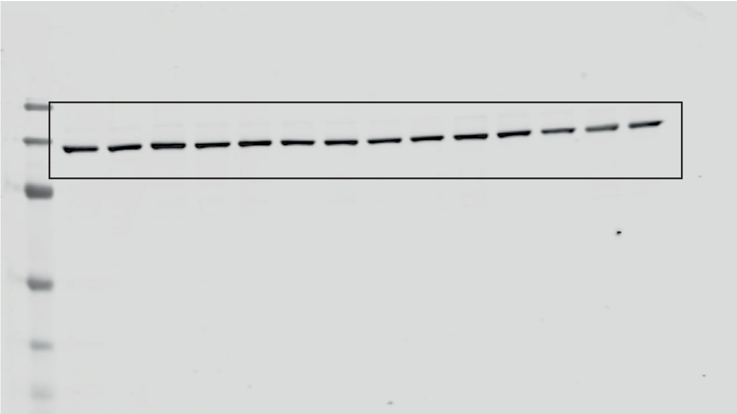

Vinculin

HCT116; BPDE

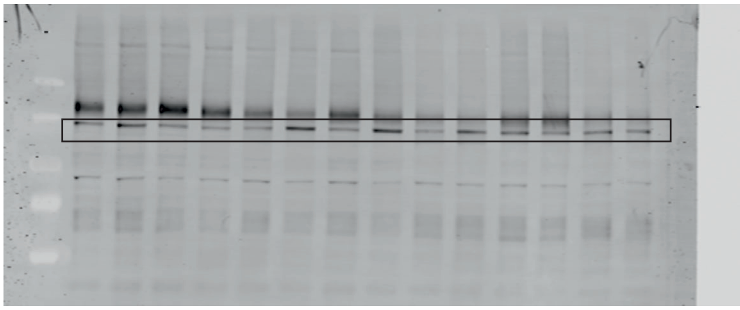

XPC

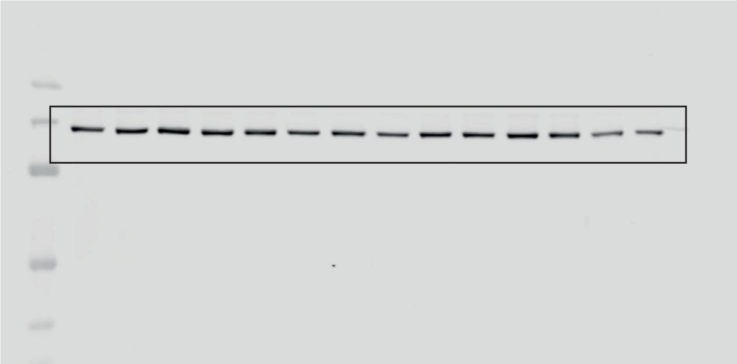

Vinculin

Figure S4:

iPS12; Etoposide

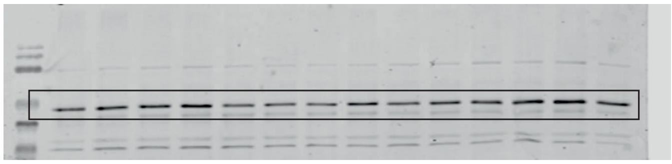

p53

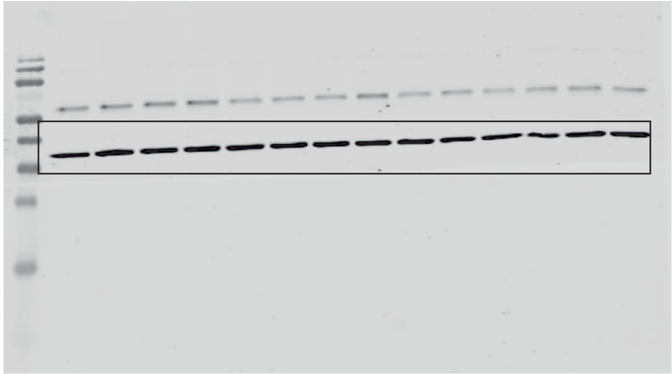

GAPDH

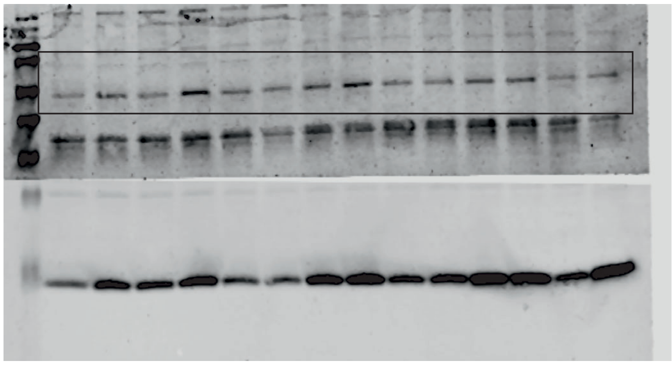

pp53 (Ser15)

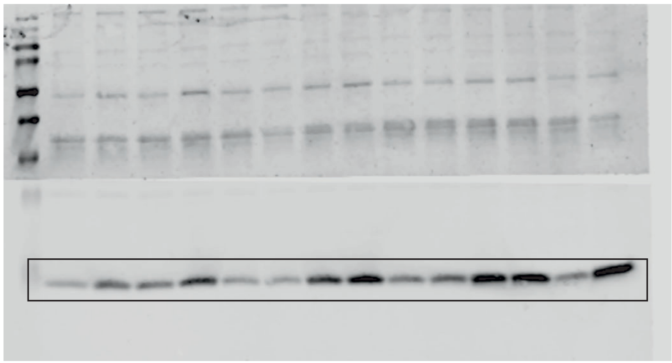

γH2AX

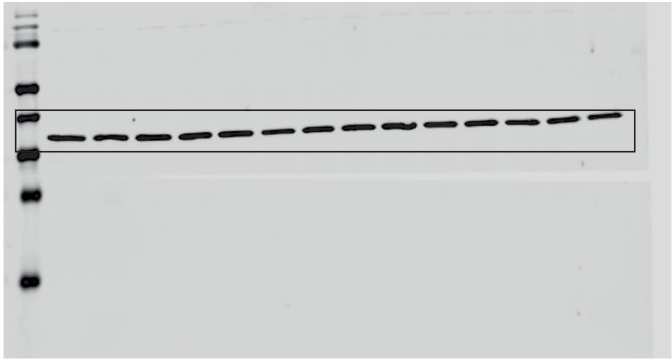

GAPDH

iPS12; BPDE

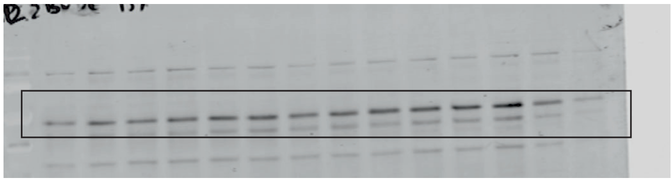

p53

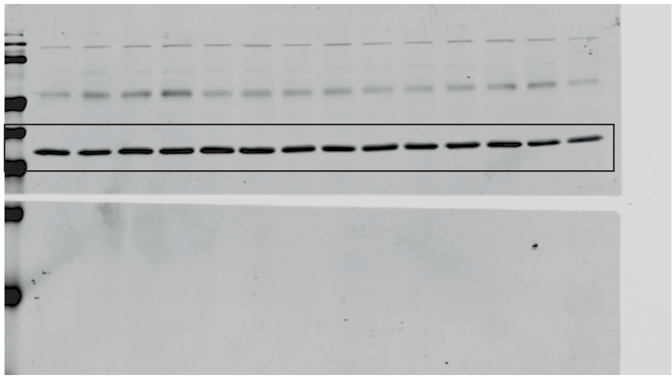

GAPDH

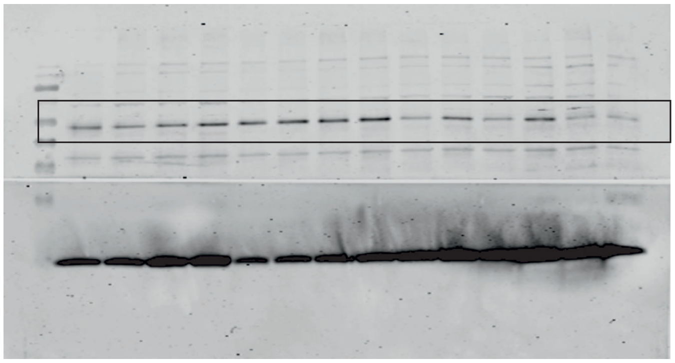

pp53 (Ser15)

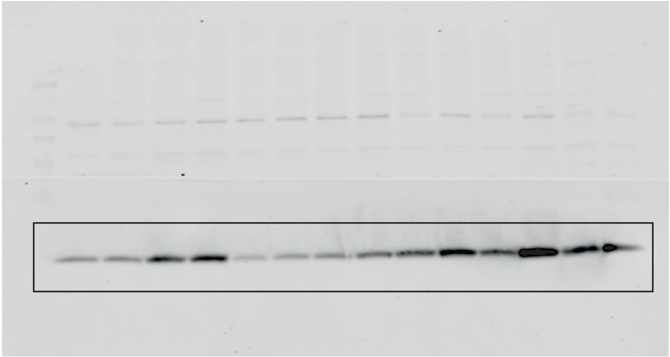

γH2AX

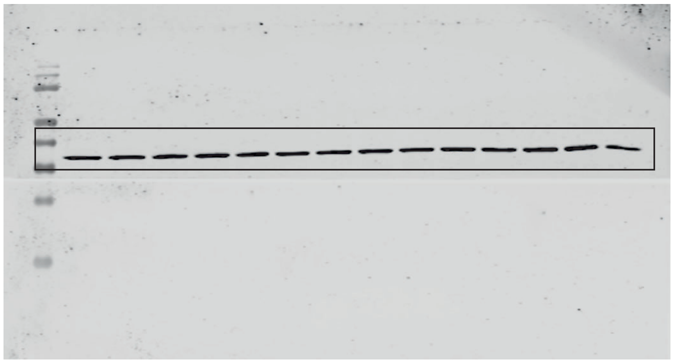

GAPDH

Figure S4:

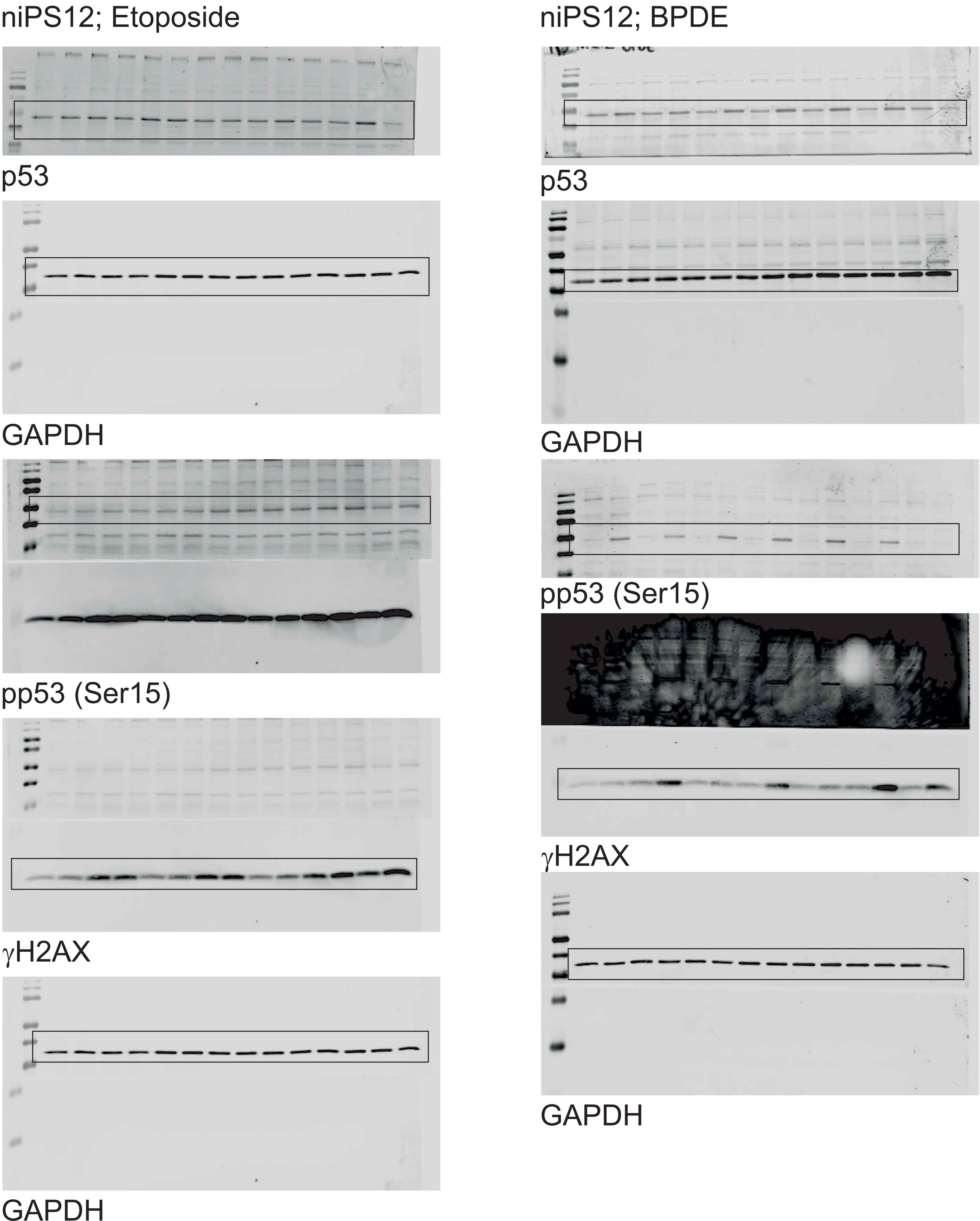
niPS12; Etoposide
p53
GAPDH
pp53 (Ser15)
γH2AX
GAPDH
niPS12; BPDE
p53
GAPDH
pp53 (Ser15)
γH2AX
GAPDH

Figure S4:

iPS12; Etoposide

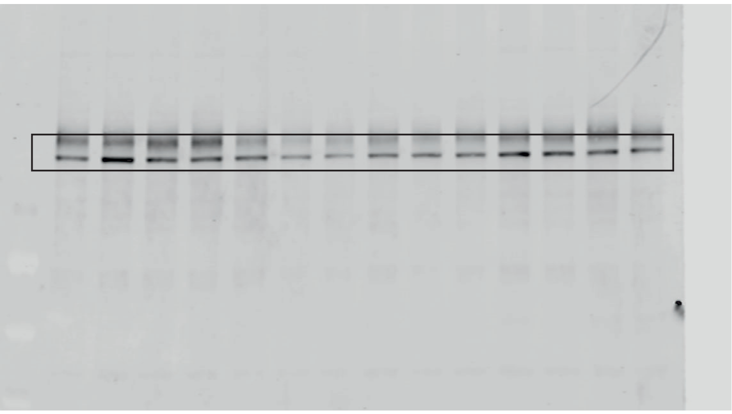

XPC

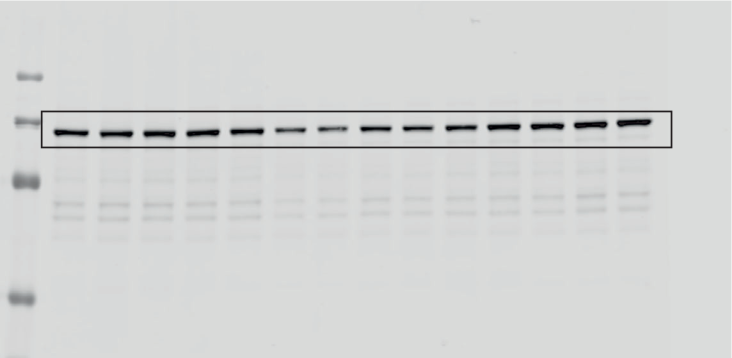

Vinculin

iPS12; BPDE

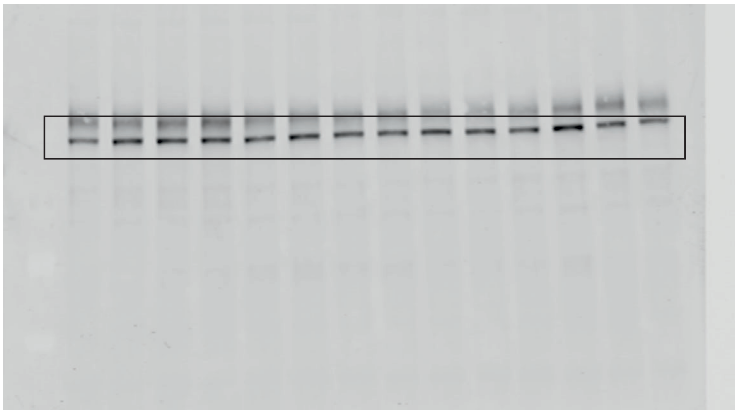

XPC

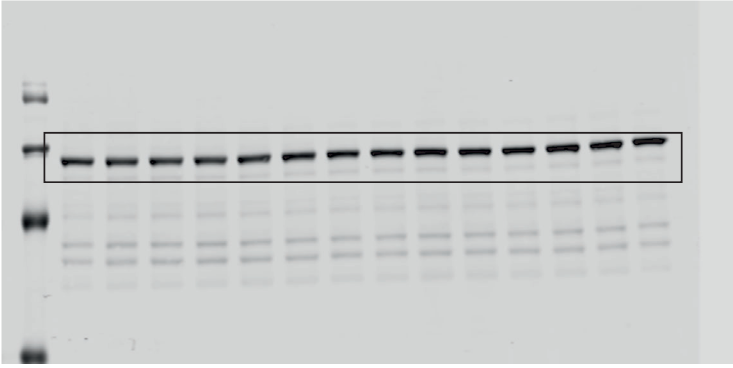

Vinculin

niPS12; Etoposide

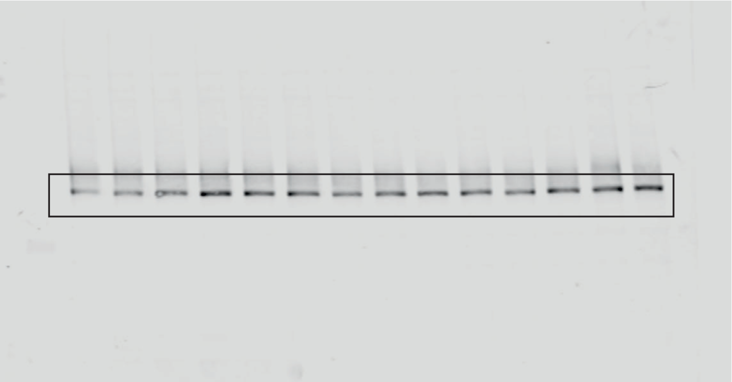

XPC

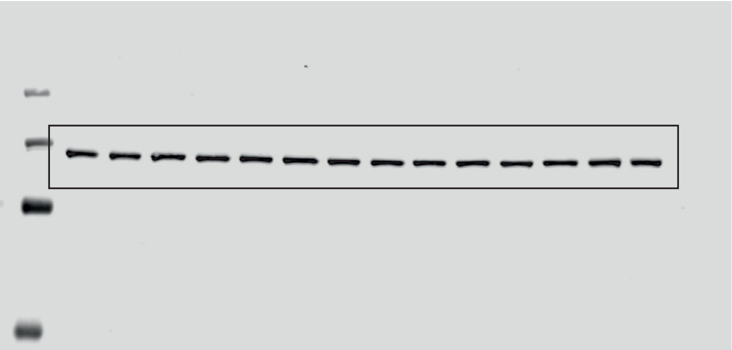

Vinculin

niPS12; BPDE

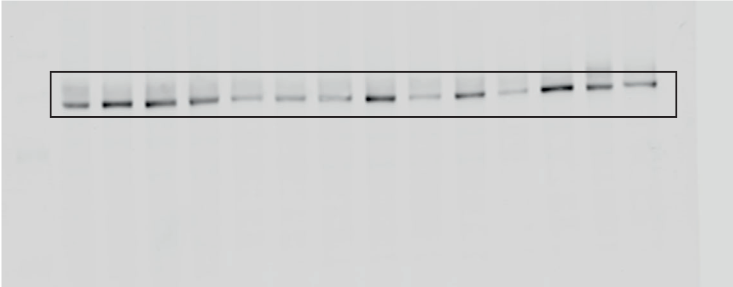

XPC

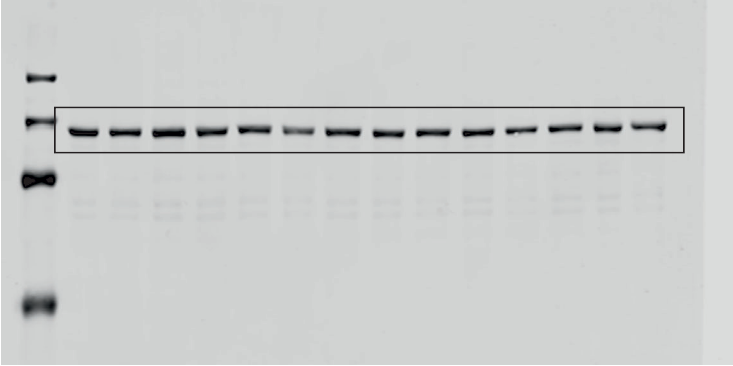

Vinculin

Figure S5:

iPS12; Etoposide

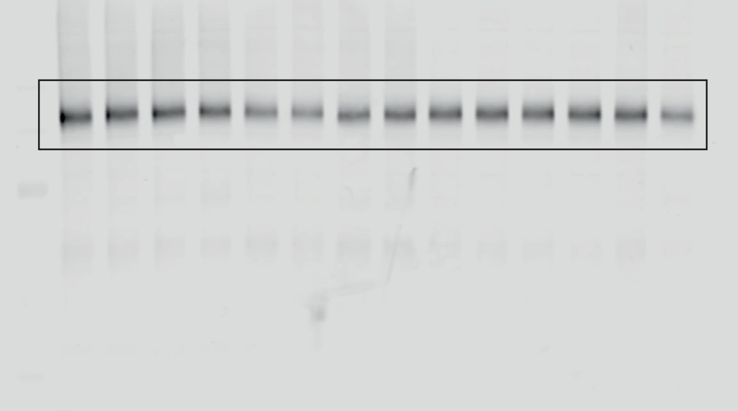

ULK1

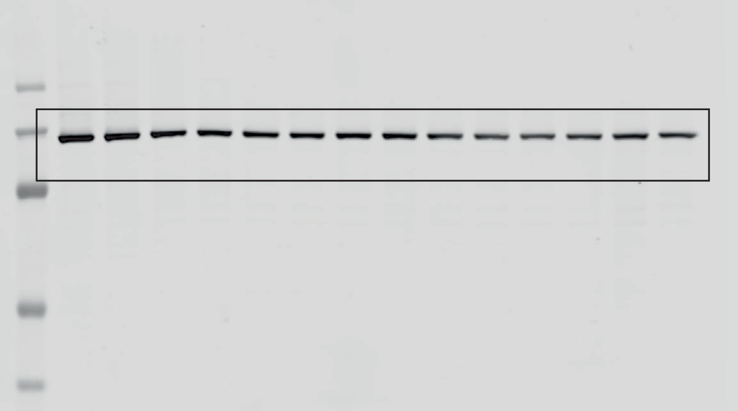

Vinculin

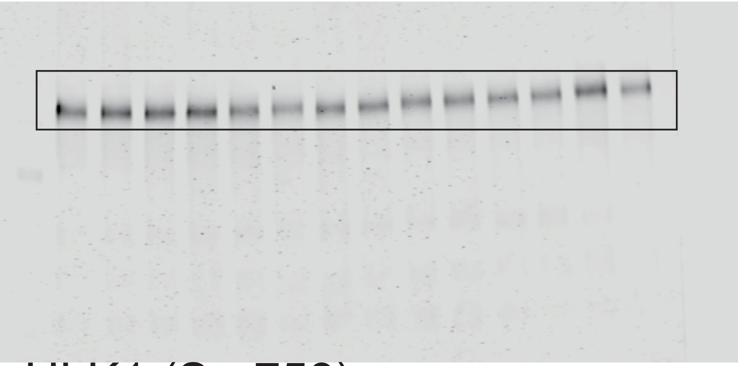

pULK1 (Ser758)

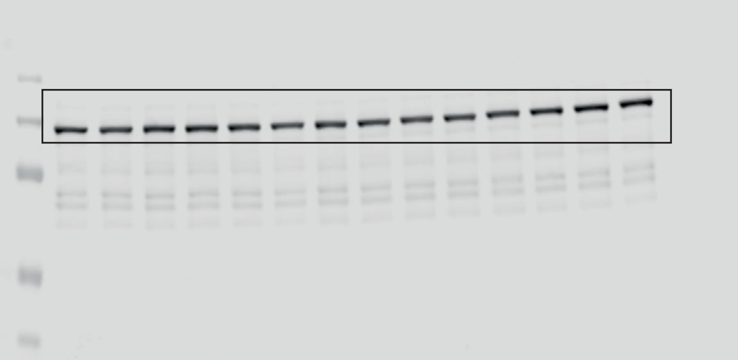

Vinculin

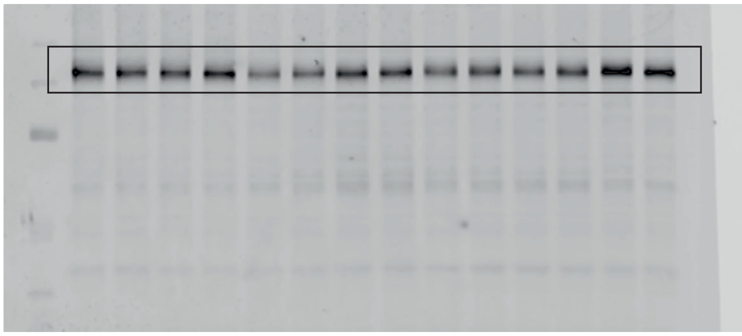

pULK1 (Ser638)

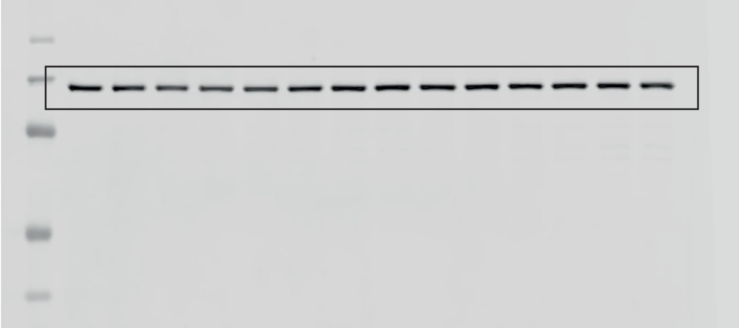

Vinculin

Figure S5:

niPS12; Etoposide

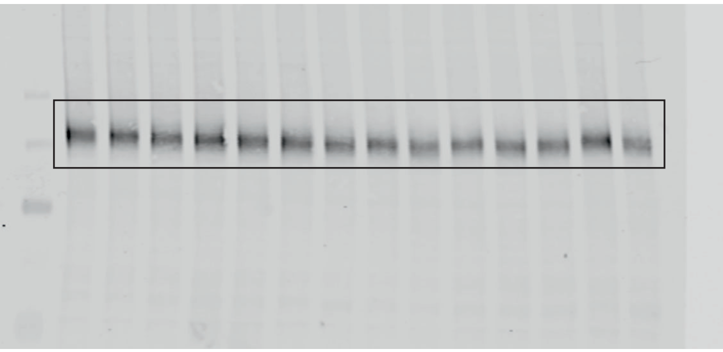

ULK1

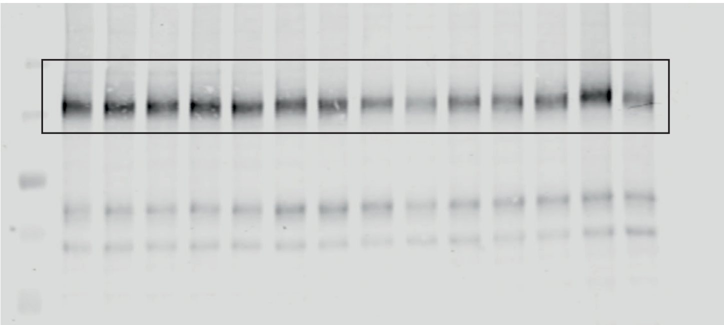

pULK1 (Ser638)

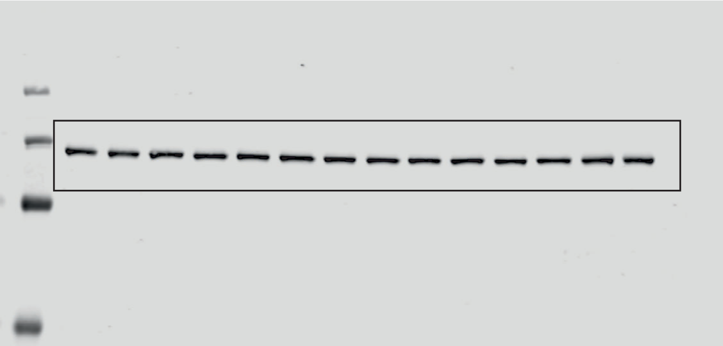

Vinculin

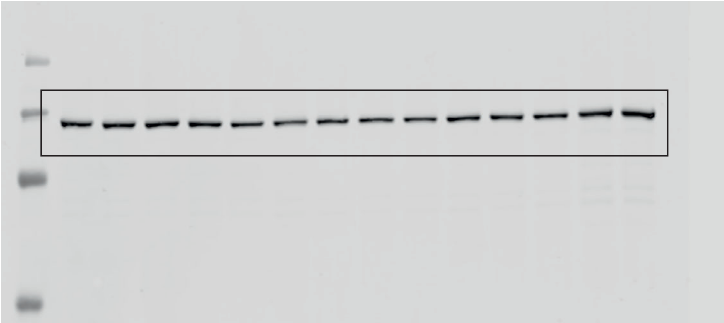

Vinculin

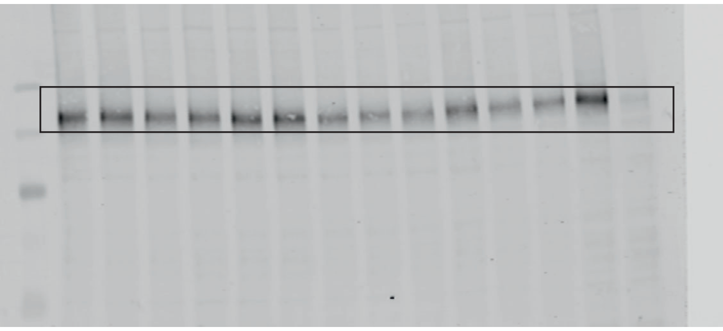

pULK1 (Ser758)

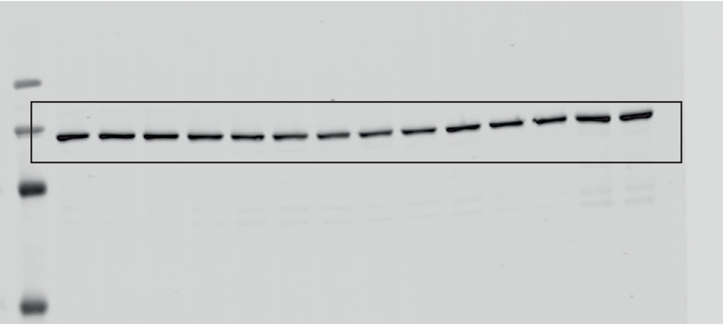

Vinculin

Figure S6:

iPS12; BPDE

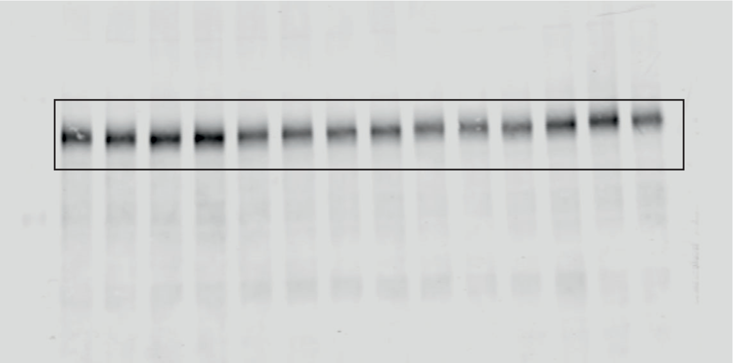

ULK1

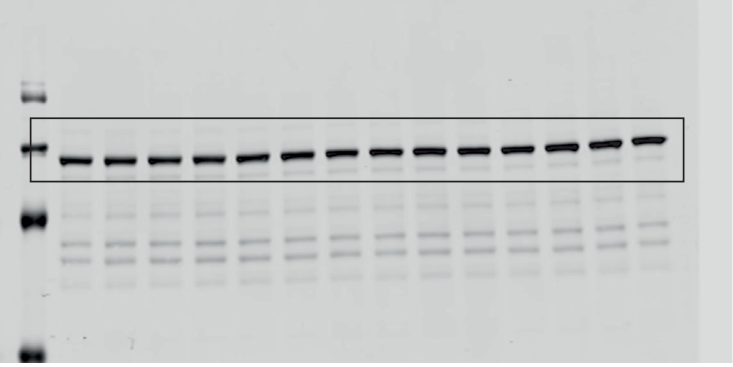

Vinculin

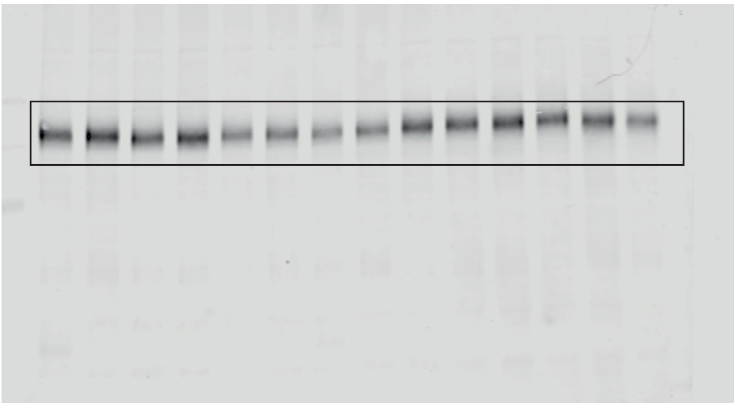

pULK1 (Ser758)

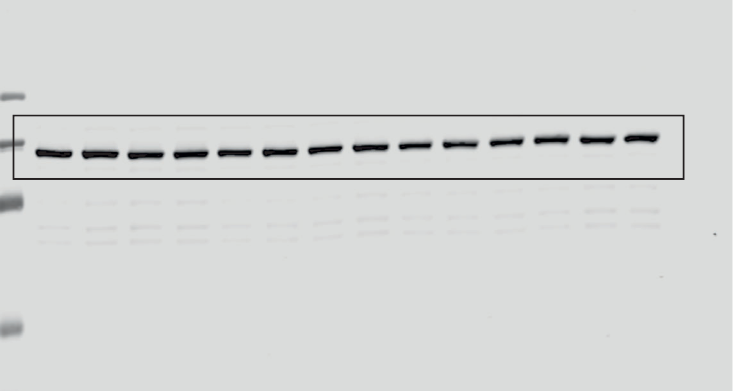

Vinculin

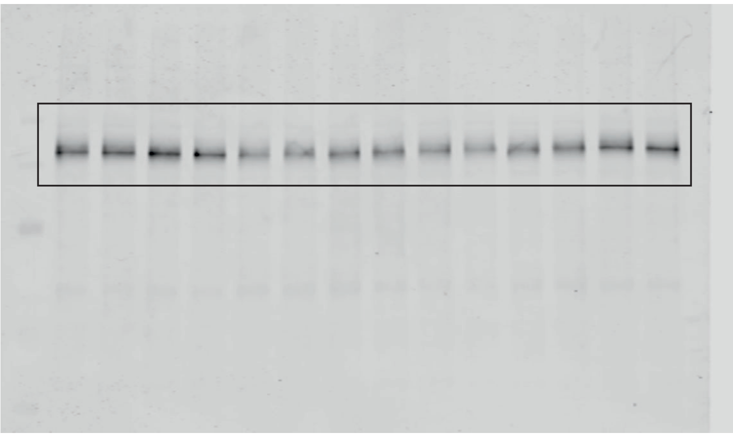

pULK1 (Ser638)

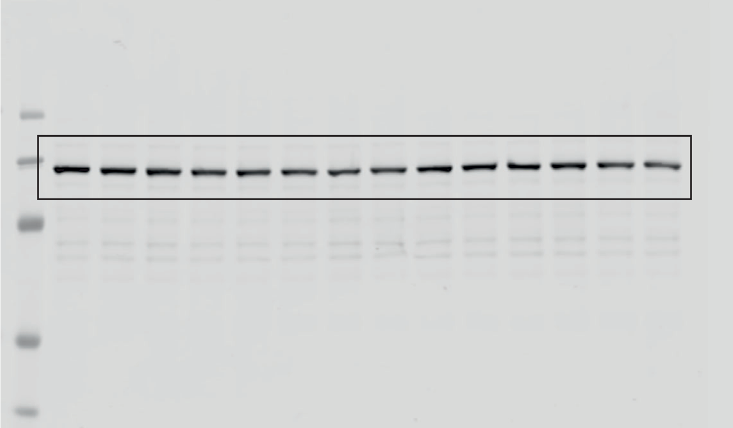

Vinculin

Figure S6:

niPS12; BPDE

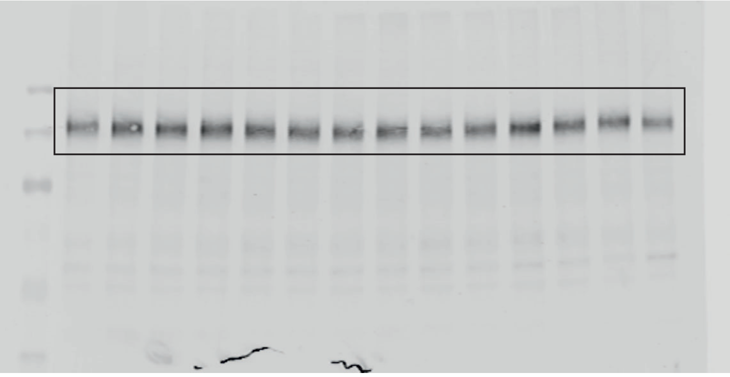

ULK1

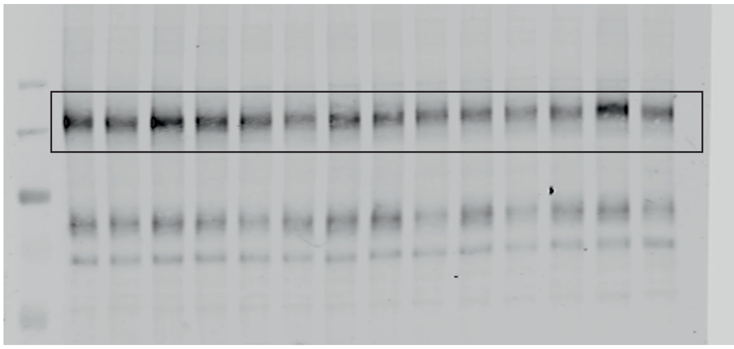

pULK1 (Ser638)

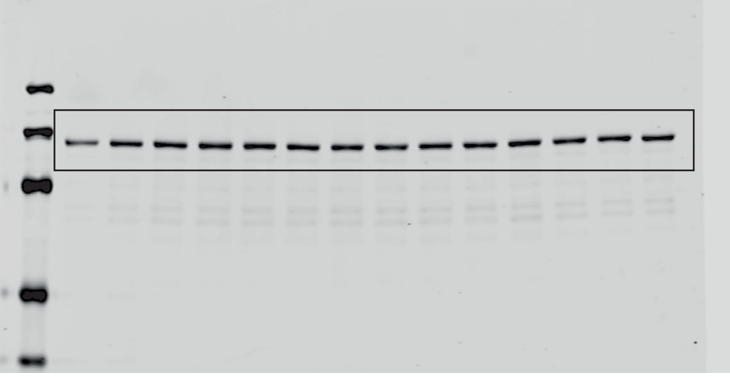

Vinculin

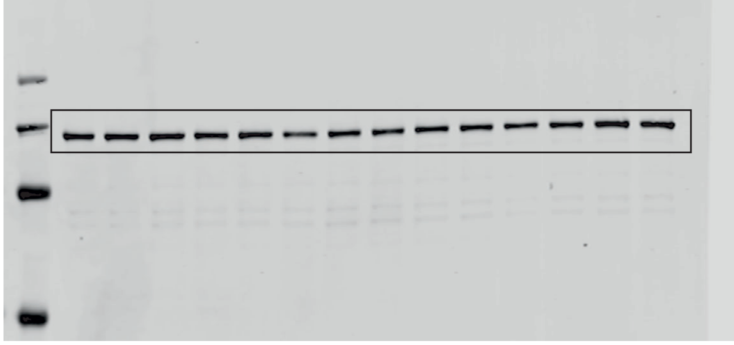

Vinculin

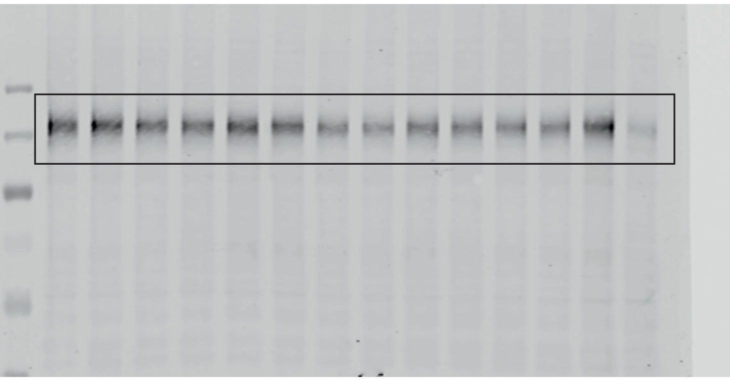

pULK1 (Ser758)

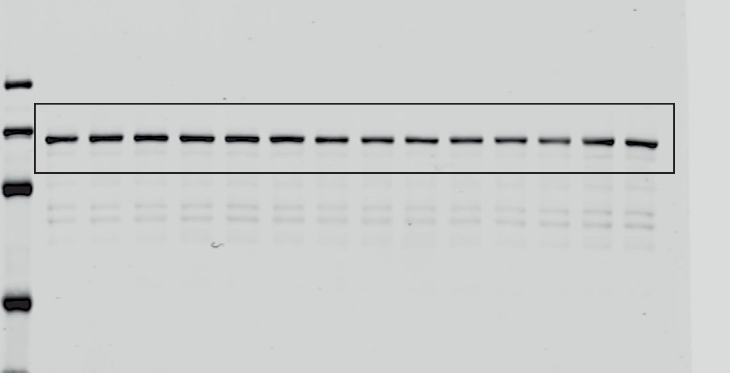

Vinculin

Figure S7:

iPS11; BPDE & Etoposide

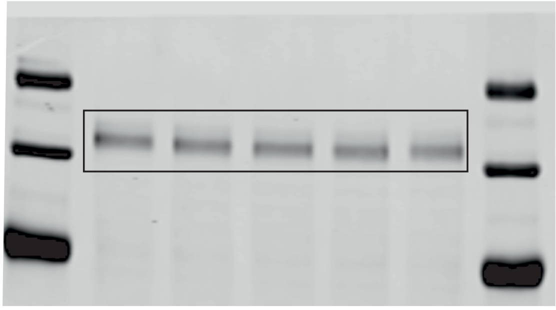

ULK1

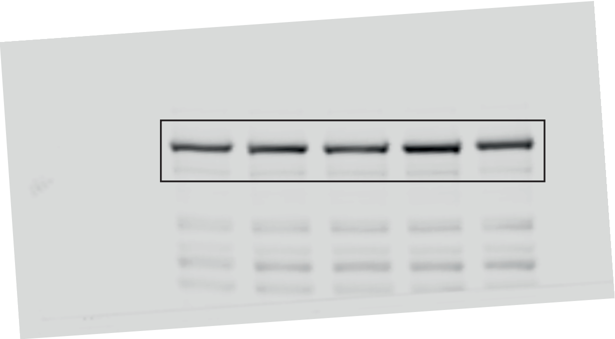

Vinculin

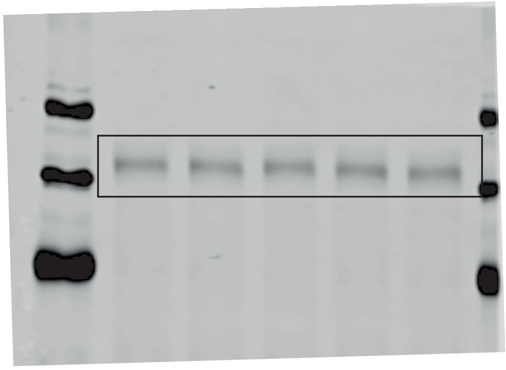

pULK1 (Ser638)

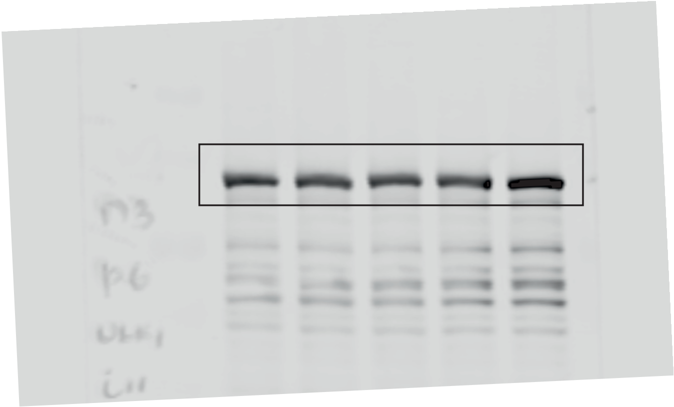

Vinculin

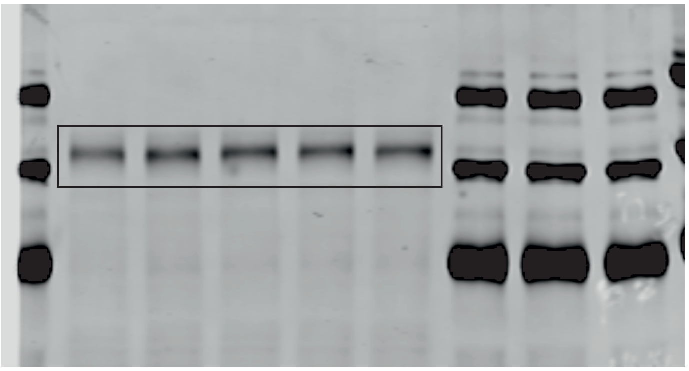

pULK1 (Ser758)

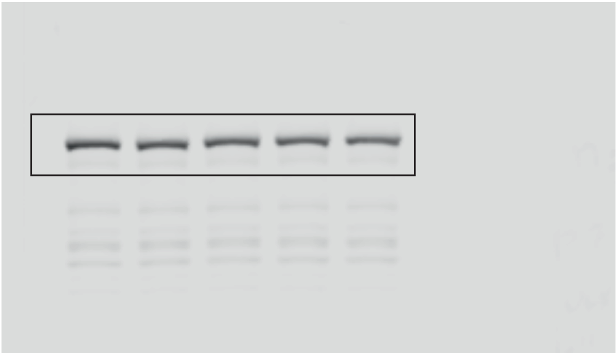

Vinculin

Figure S8:

iPS12; Etoposide

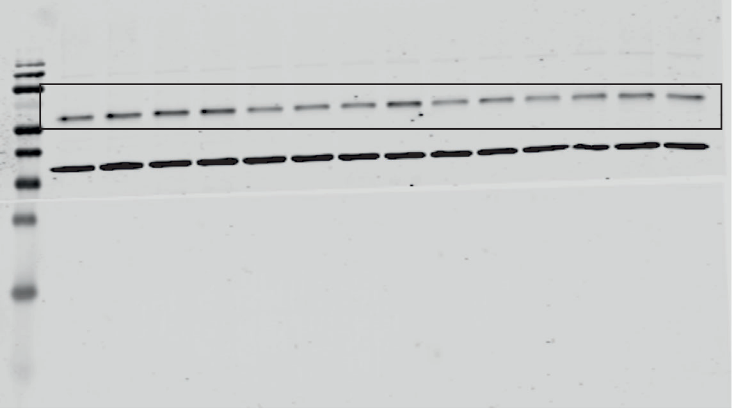

p62

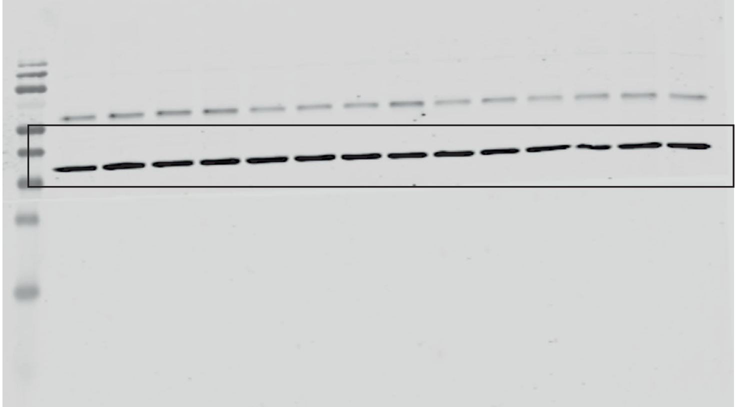

GAPDH

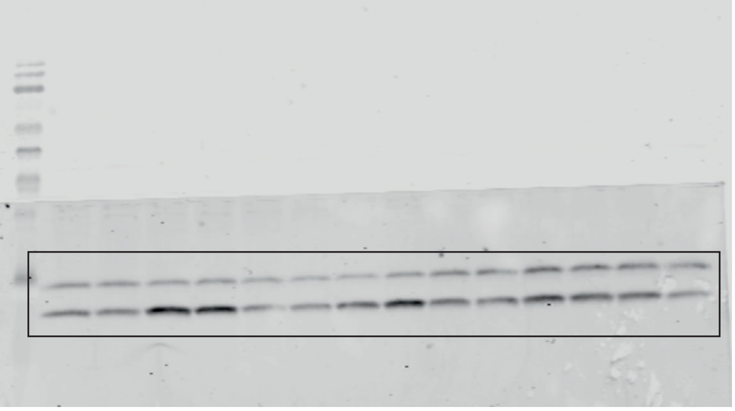

LC3

iPS12; BPDE

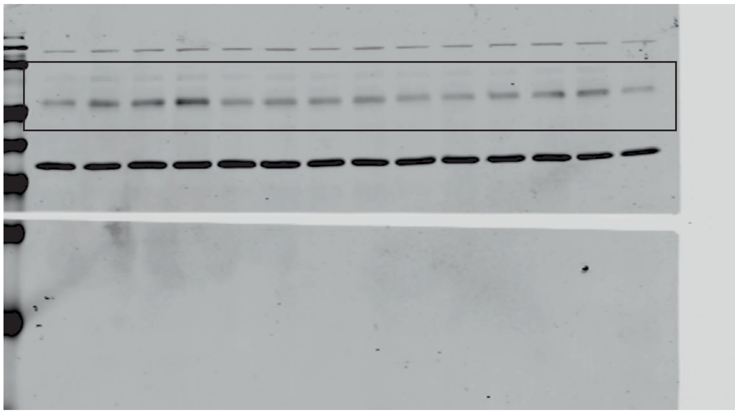

p62

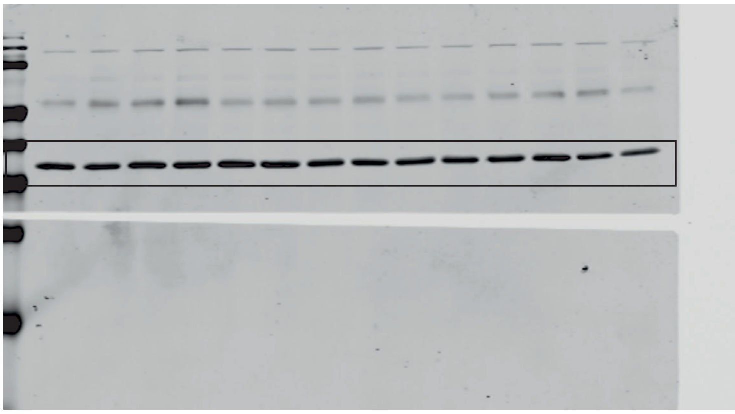

GAPDH

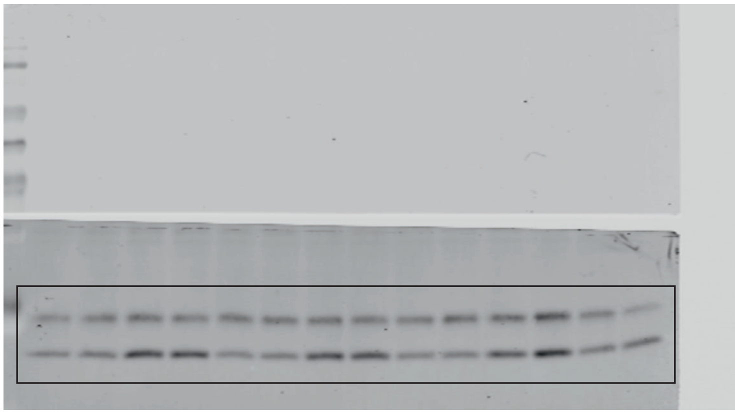

LC3

Figure S8:

niPS12; Etoposide

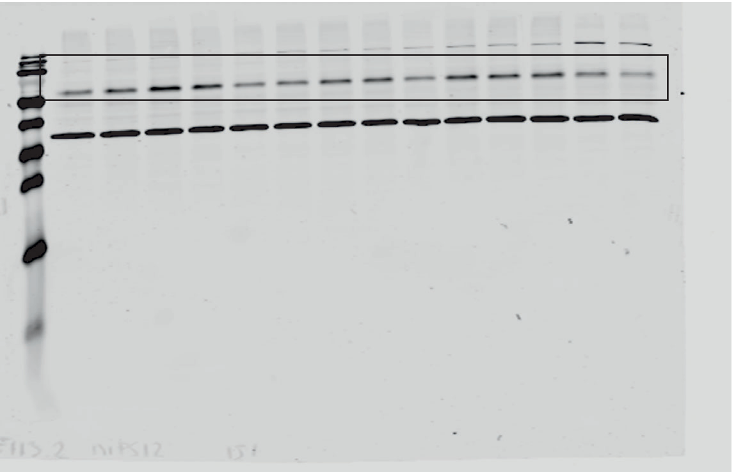

p62

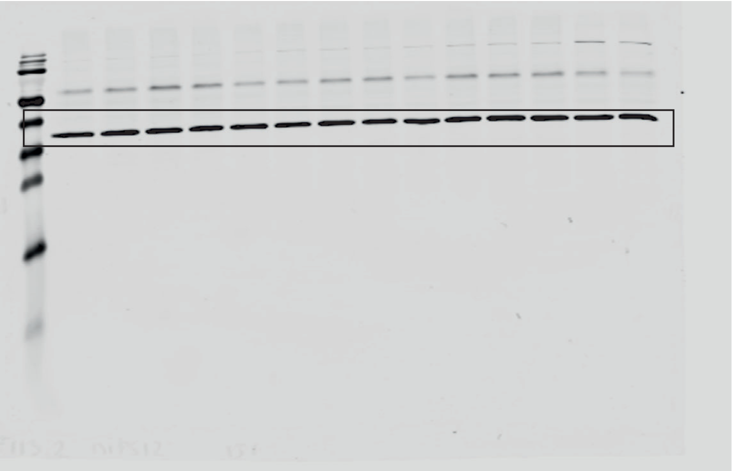

GAPDH

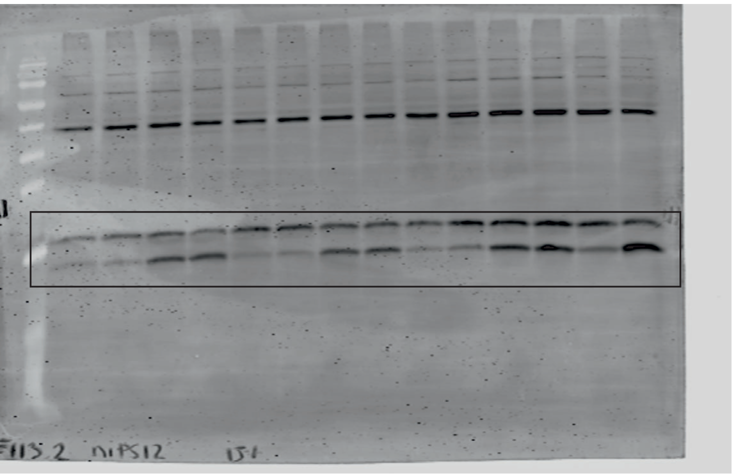

LC3

niPS12; BPDE

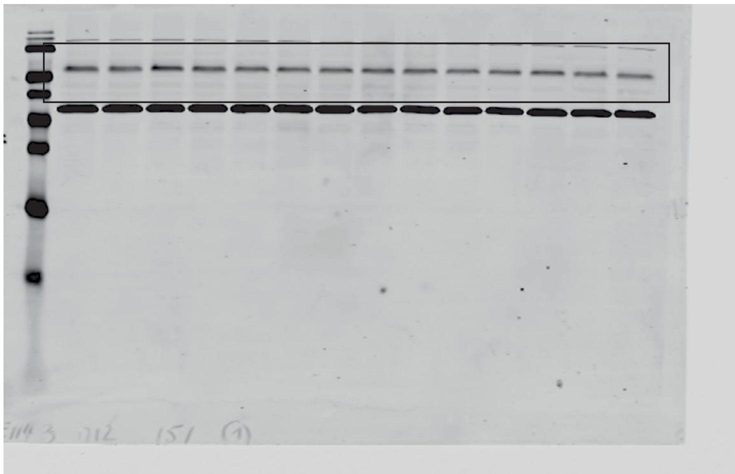

p62

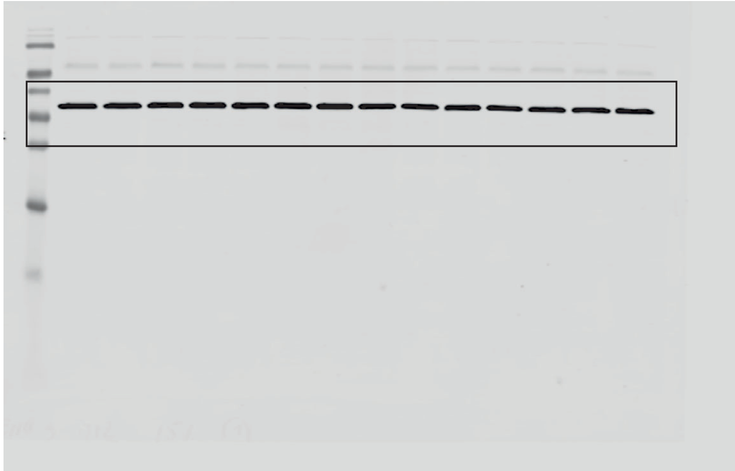

GAPDH

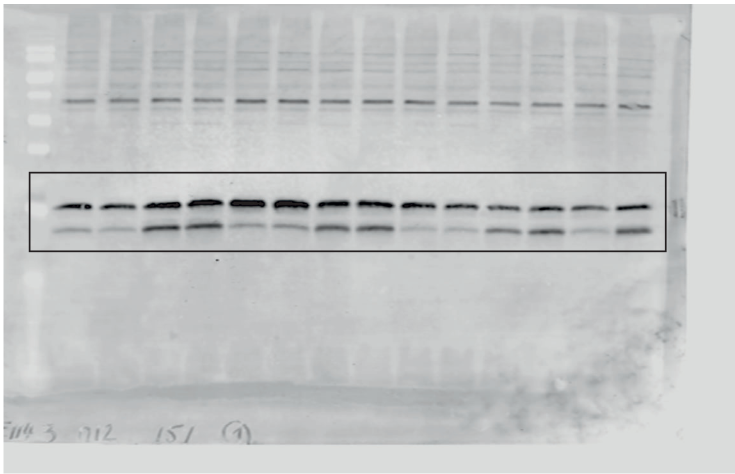

LC3

Figure S9:

iPS11; BPDE & Etoposide

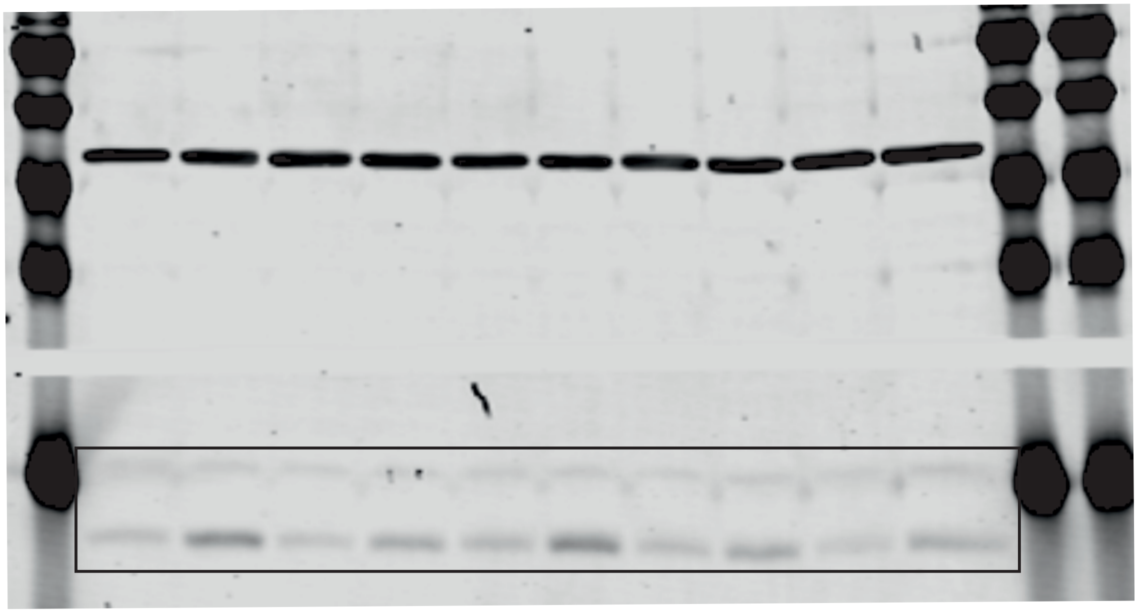

LC3

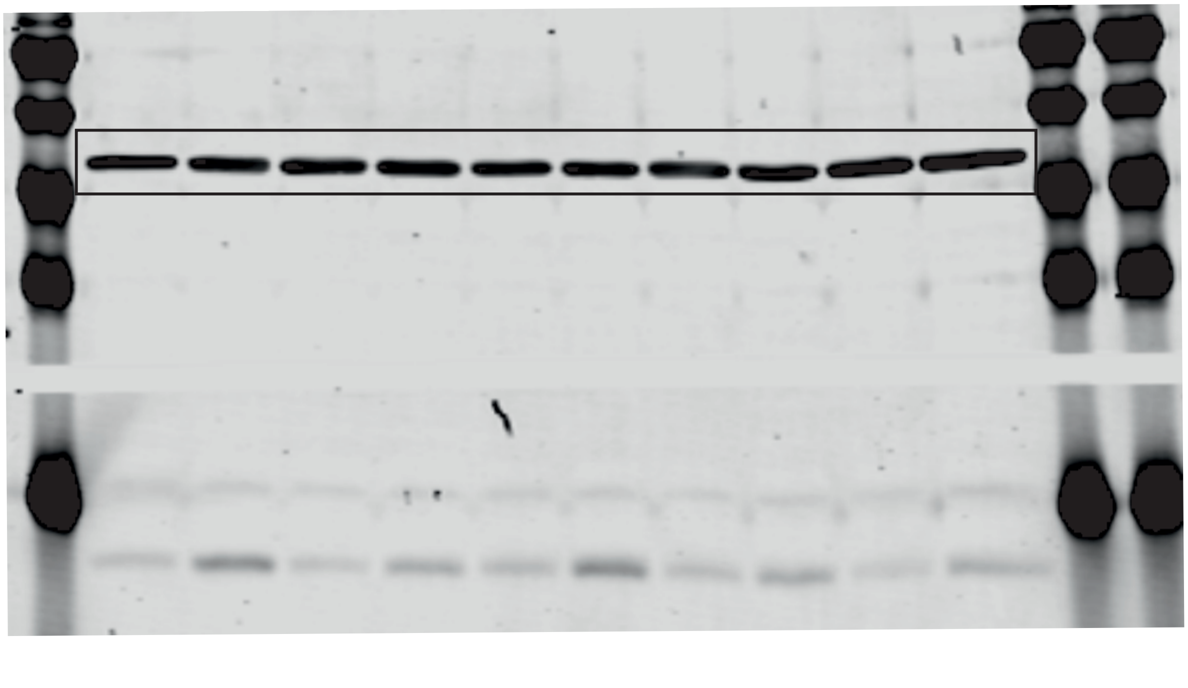

GAPDH

Figure S9:

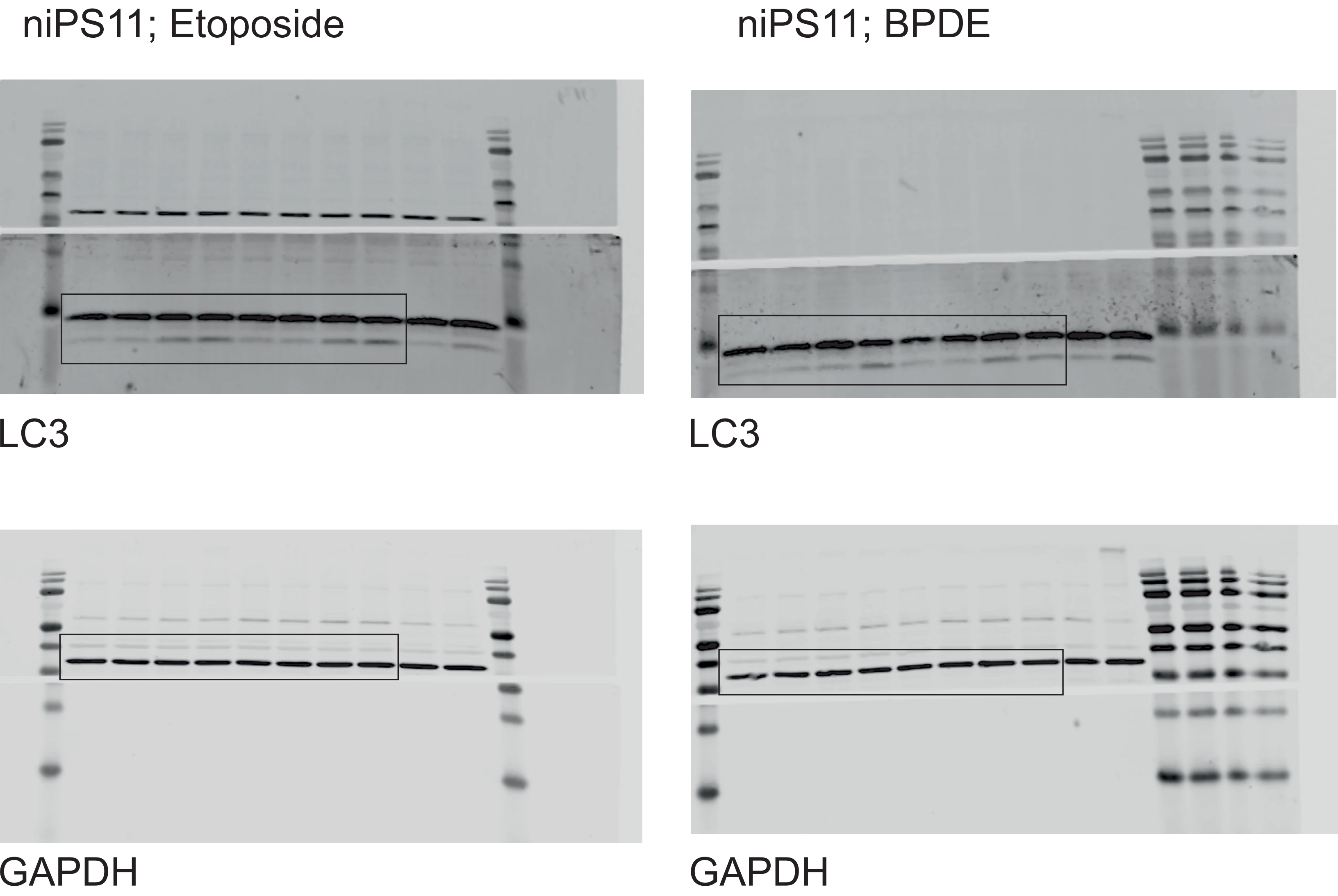

Figure S9:

HCT116; Etoposide

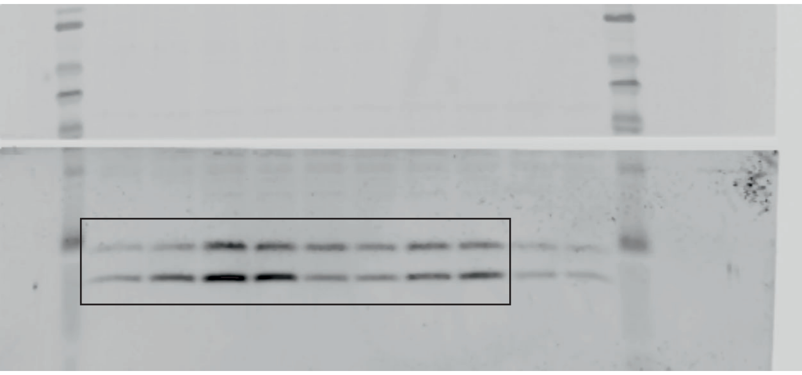

LC3

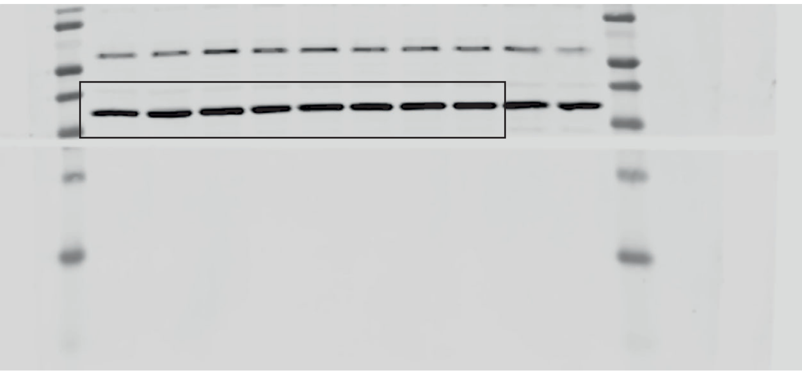

GAPDH

HCT116; BPDE

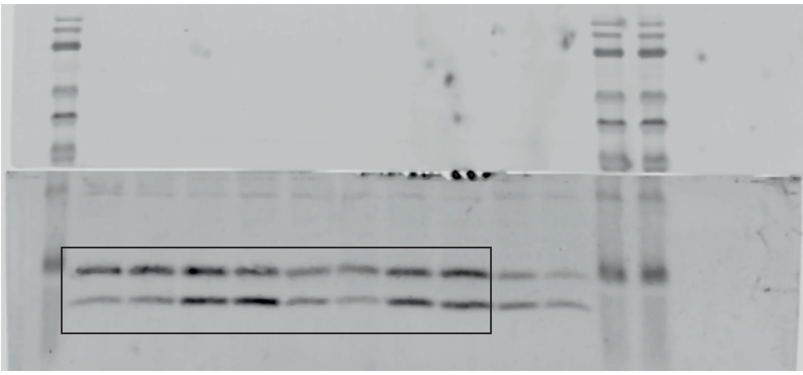

LC3

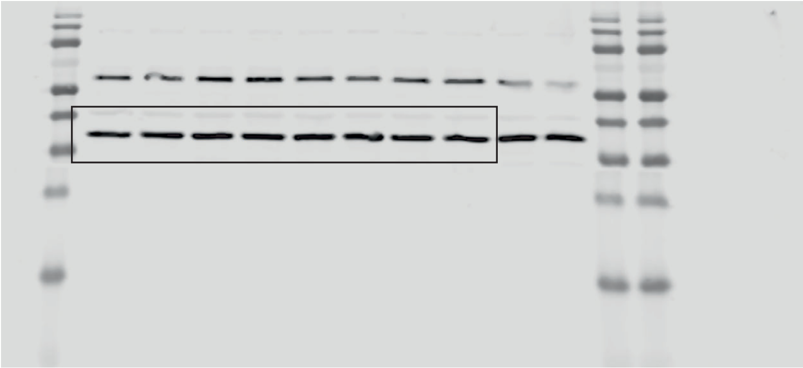

GAPDH

Figure S10:

iPS11; Etoposide, BPDE (IC50)

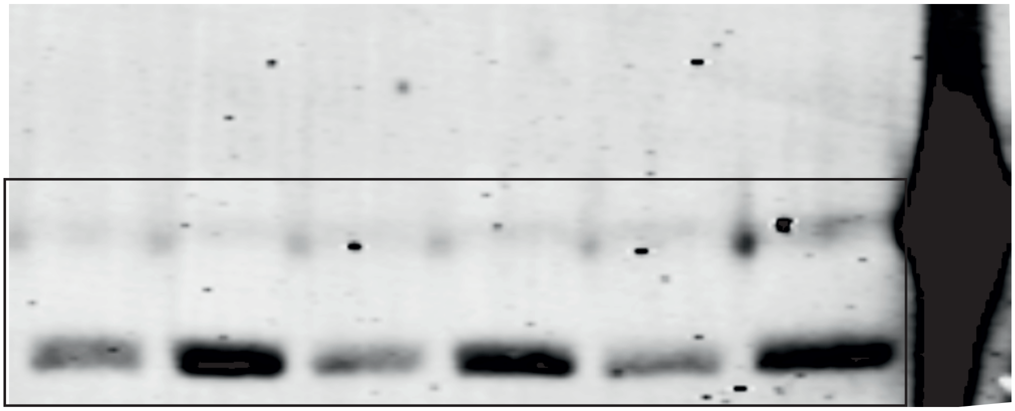

LC3

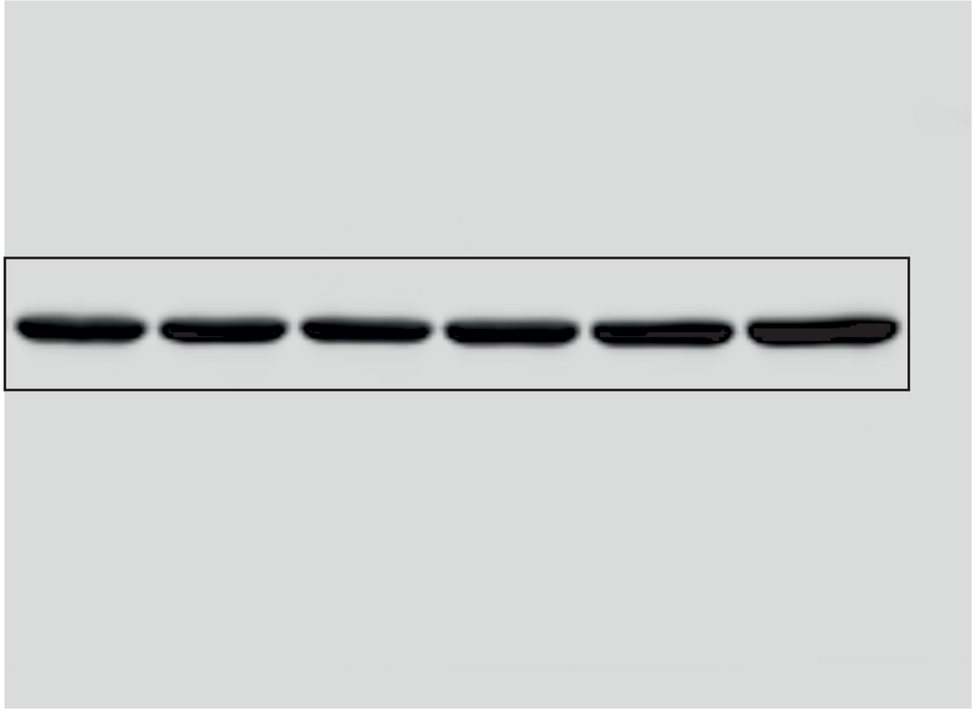

β-actin

Figure S11:

niPS11; Etoposide (24h)

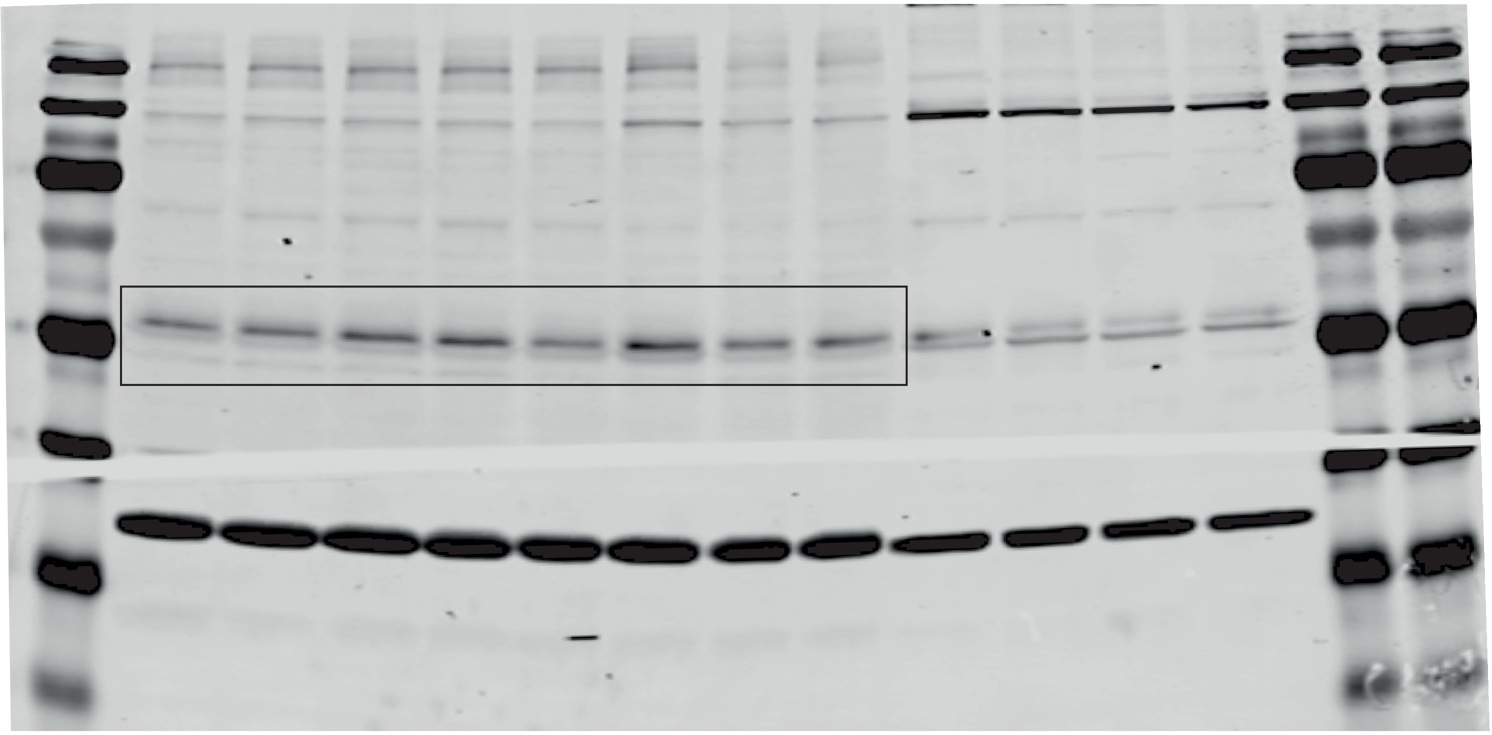

SESN2

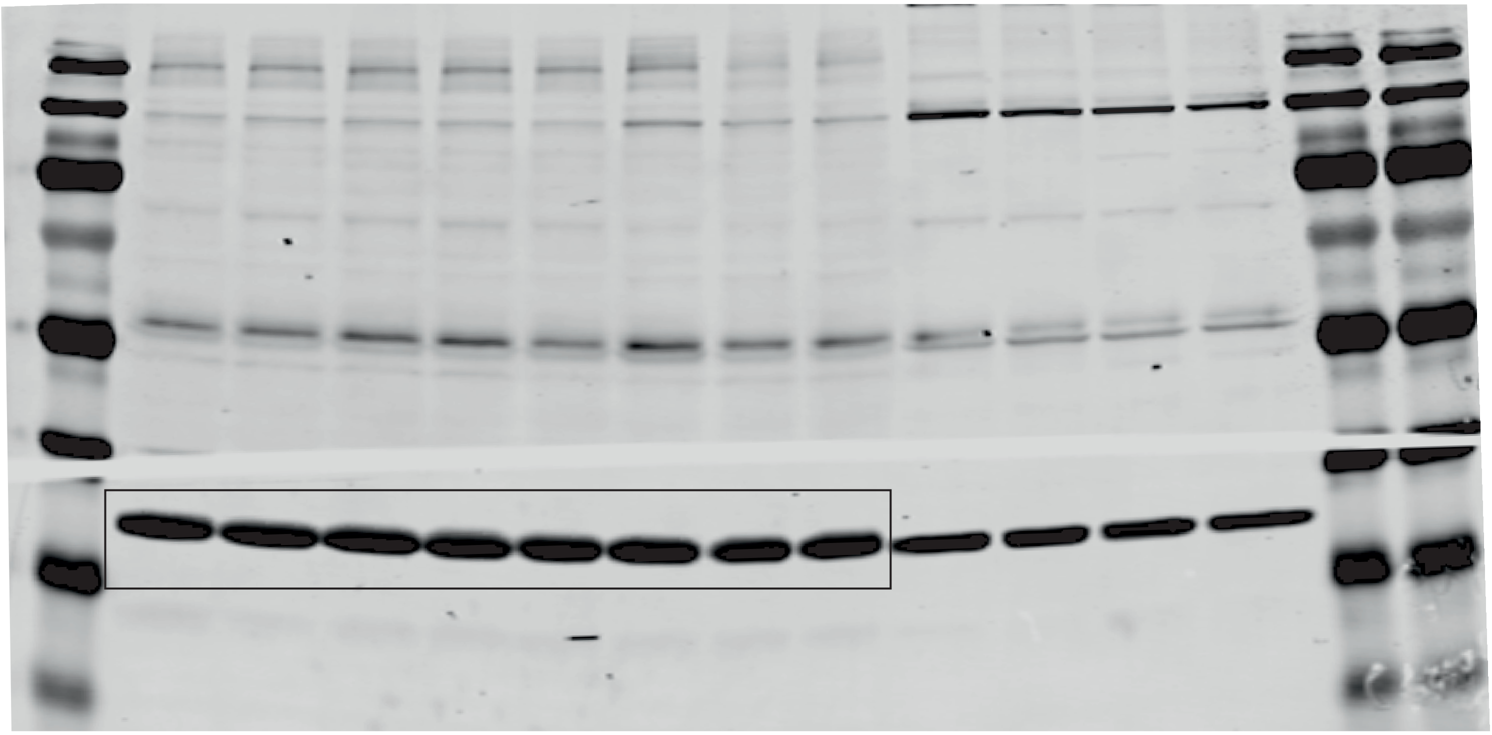

β-actin

Figure S13:

niPS12; Etoposide

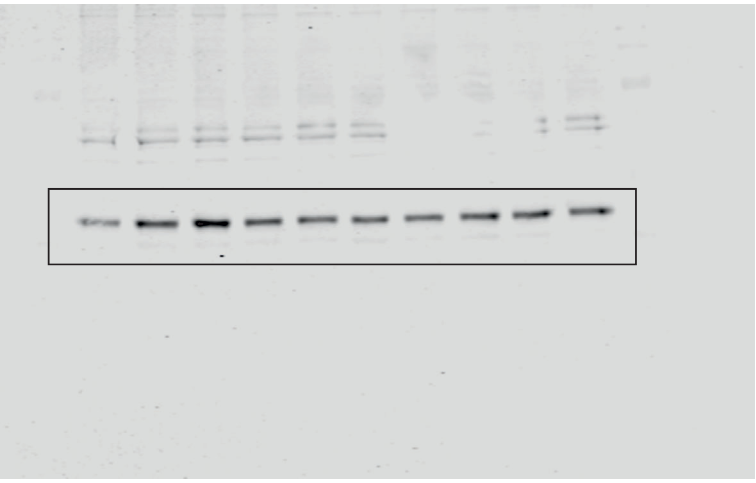

Aurora A

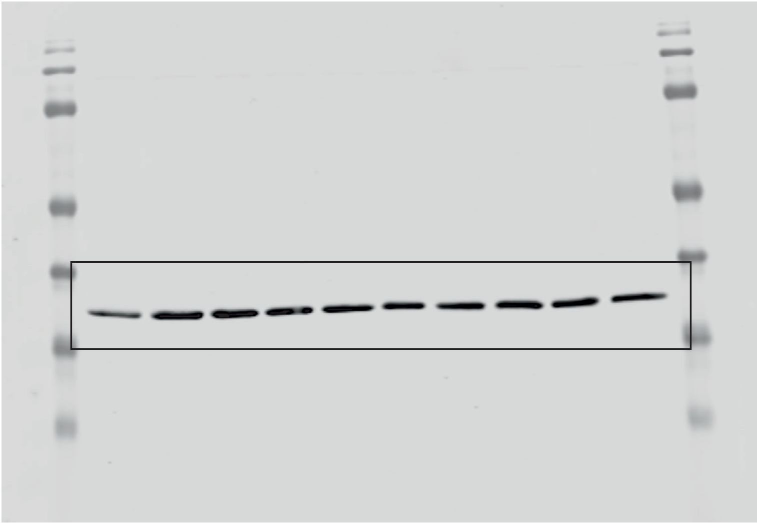

GAPDH

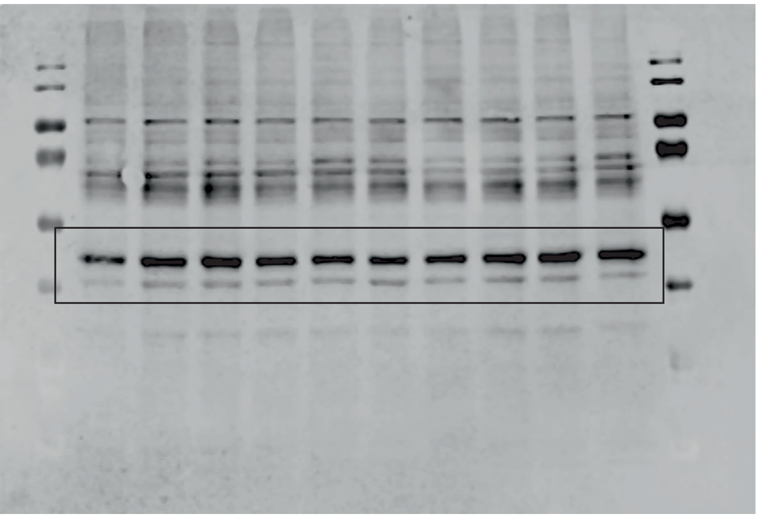

PLK1

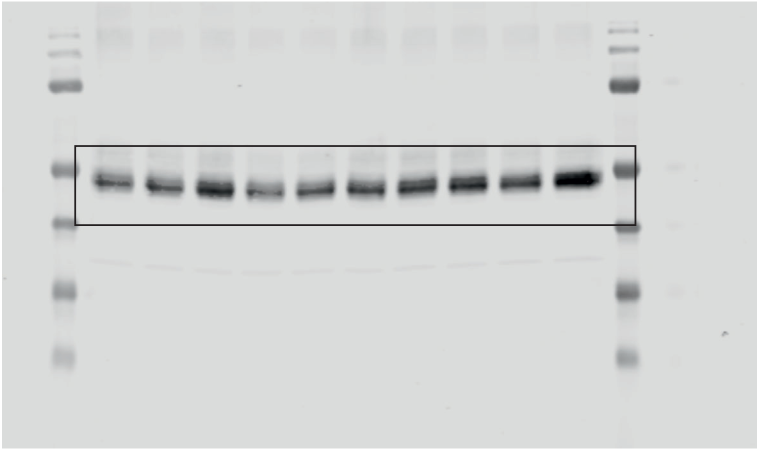

Acetylated Tubulin

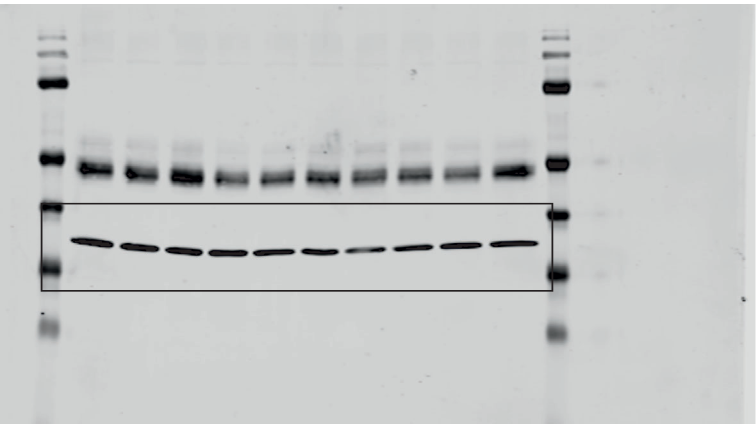

GAPDH

Figure S13:

niPS12; BPDE

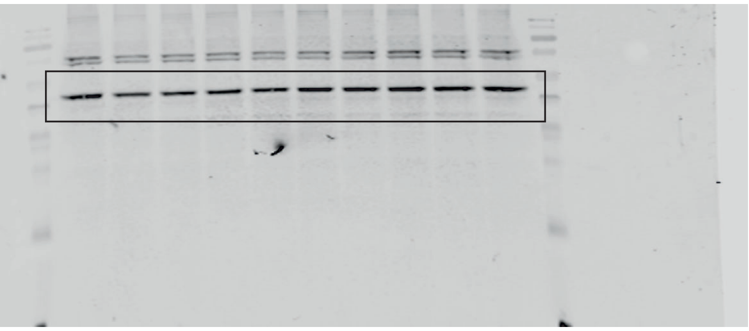

Aurora A

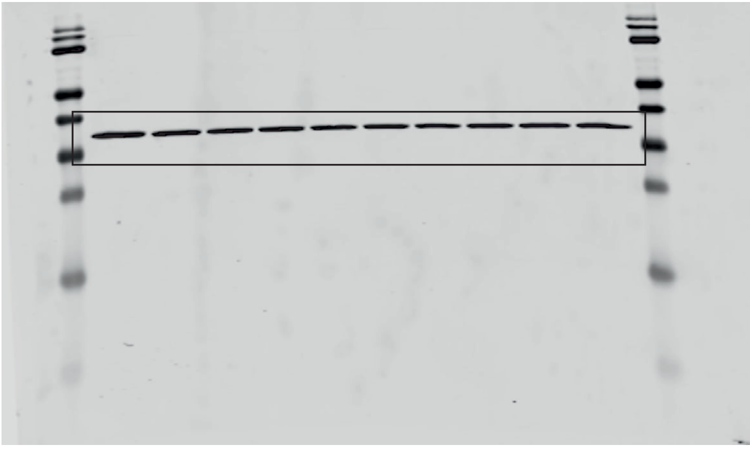

GAPDH

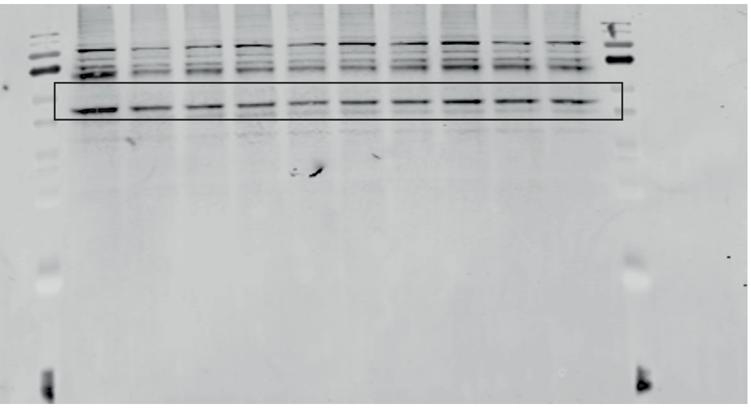

PLK1

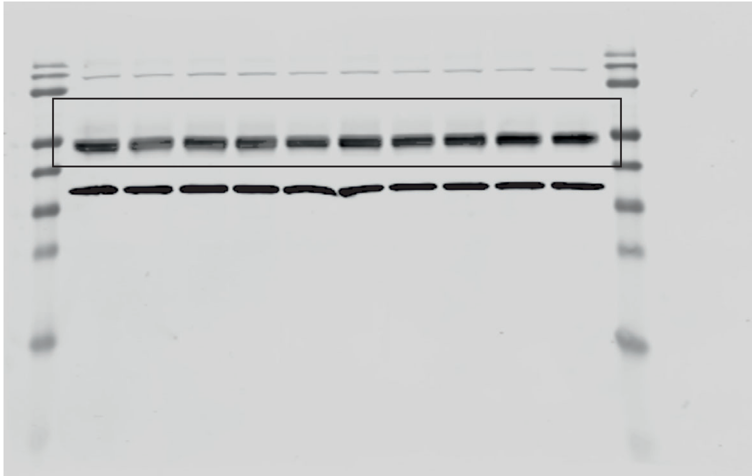

Acetylated Tubulin

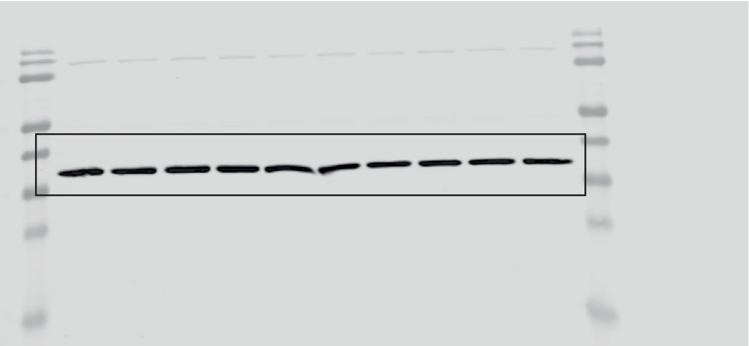

GAPDH
