# Supplementary material for: Canonical autophagy remains inactive in induced pluripotent stem cells and neuronal progenitor cells following DNA damage induced by BPDE or etoposide
Source: Sci Rep. 2026 Jun 8;16:18028. doi: 10.1038/s41598-026-54127-6 (PMC13254337; doi:10.1038/s41598-026-54127-6)
Supplement: Supplementary file 2 — Supplementary Material 2 [file 41598_2026_54127_MOESM2_ESM.pdf]

Supporting Information

Canonical autophagy remains inactive in induced pluripotent stem cells and neuronal progenitor cells following DNA damage induced by BPDE or etoposide

AUTHORS/AFFILIATIONS

Seda Akgün[1], Padmashri Naren[1,#], Thomas Lenz[2,#], Annika Zink[3], Karina Stephanie Krings[1], Sebastian Wesselborg[1], María José Mendiburo[1], Alessandro Prigione[3], Kai Stühler[1,2], Björn Stork[1,*]

[1]*Institute of Molecular Medicine I, Medical Faculty and University Hospital Düsseldorf, Heinrich Heine University, 40225 Düsseldorf, Germany*

[2]*Molecular Proteomics Laboratory, Biological Medical Research Center, Heinrich Heine University Düsseldorf, 40225 Düsseldorf, Germany*

[3]*Department of General Pediatrics, Neonatology and Pediatric Cardiology, Medical Faculty and University Hospital Düsseldorf, Heinrich Heine University, 40225 Düsseldorf, Germany*

# These authors contributed equally

CONTACT

*Corresponding author:

Björn Stork, Universitätsstr. 1, Building 22.03, 40225 Düsseldorf, Germany

Tel.: +49 (0)211 81 11954, E-mail: bjoern.stork@hhu.de

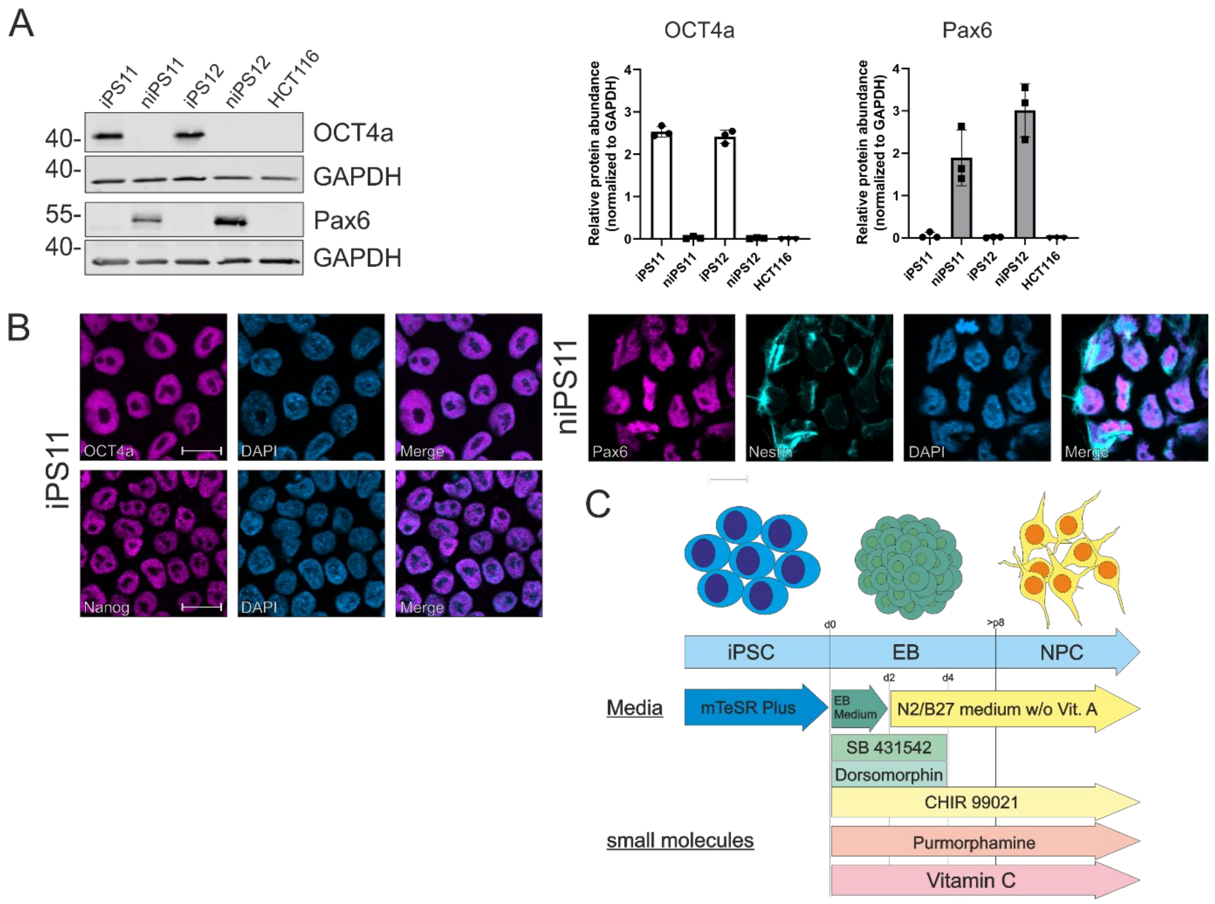

Figure S1: Verification of induced pluripotent stem cells and thereof differentiated neural progenitor cells. (**A**) Induced pluripotent stem cells (iPS11/12), neural progenitor cells (niPS11/12) and HCT116 were lysed, and cellular lysates were immunoblotted for OCT4a, Pax6 and GAPDH respectively. One representative immunoblot is shown. The quantifications of indicated ratios are from three independent experiments (means ± SD). (**B**) Cells were fixed and stained for pluripotency markers OCT4a and Nanog in iPS11 and neural markers Pax6 and Nestin in niPS11 and visualized by immunofluorescence. Scale bar: 10 µm. (**C**) Differentiation of iPSCs into NPCs was achieved by SMAD and AMPK inhibition via SB 431542 and Dorsomorphin, directed and sustained as neural progenitor cells by supplementation of above mentioned compounds.

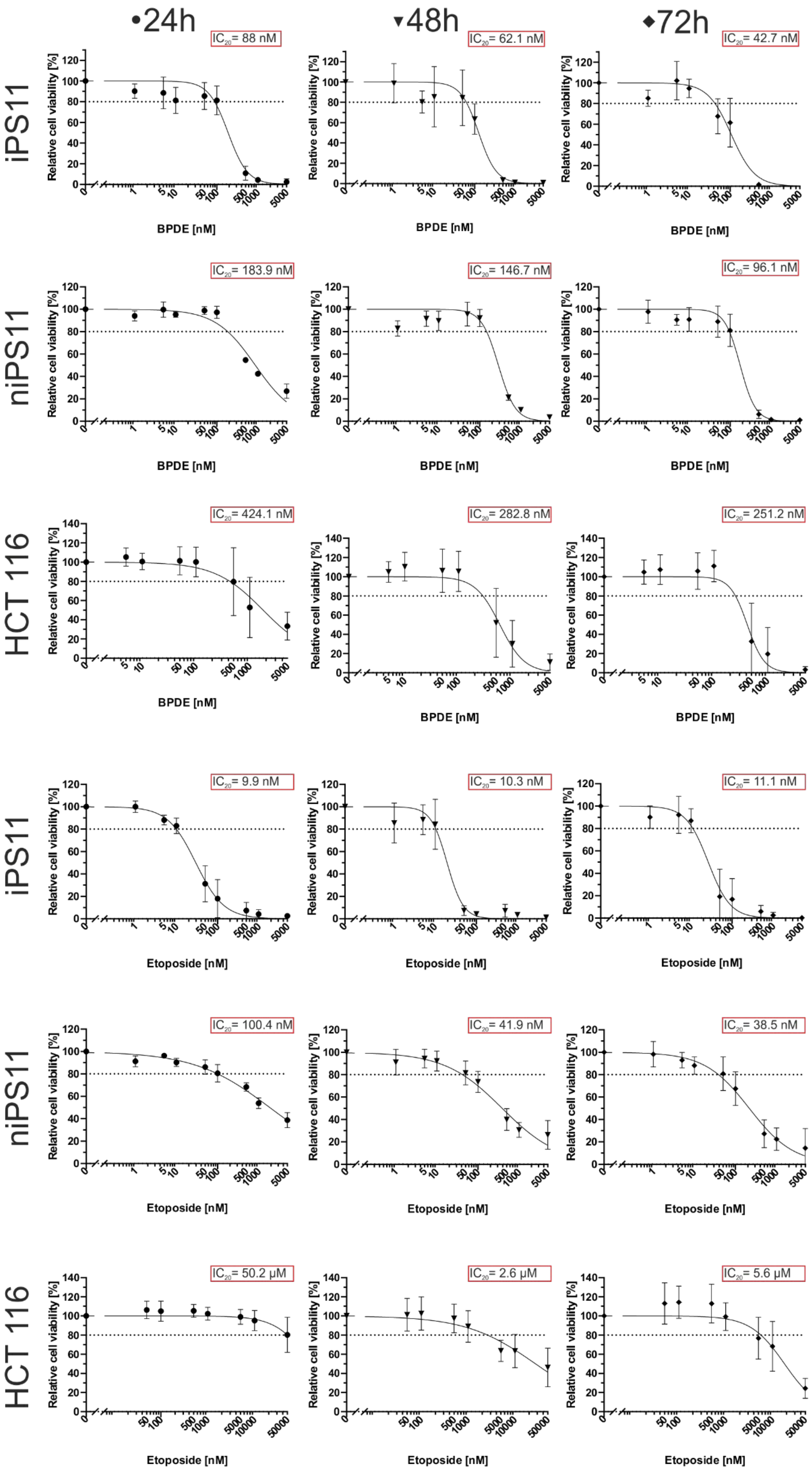

•24h
▾48h
◆72h
iPS11
IC20= 88 nM
IC20= 62.1 nM
IC20= 42.7 nM
niPS11
IC20= 183.9 nM
IC20= 146.7 nM
IC20= 96.1 nM
HCT 116
IC20= 424.1 nM
IC20= 282.8 nM
IC20= 251.2 nM
iPS11
IC20= 9.9 nM
IC20= 10.3 nM
IC20= 11.1 nM
niPS11
IC20= 100.4 nM
IC20= 41.9 nM
IC20= 38.5 nM
HCT 116
IC20= 50.2 µM
IC20= 2.6 µM
IC20= 5.6 µM
Relative cell viability [%]
BPDE [nM]
Etoposide [nM]

Figure S2: Definition of IC_{20} values for iPS11, niPS11 and HCT116.

To identify a sublethal IC_{20} dose, cells were treated with different concentrations of BPDE and etoposide for 24, 48 or 72 h while etoposide was only supplemented for 24 h and BPDE was given daily to the cells. After treatment, cell viability was measured using a thiazolylblue (MTT) assay. Results are shown as the mean ± SD of 3-5 independent experiments performed in triplicates for each treatment.

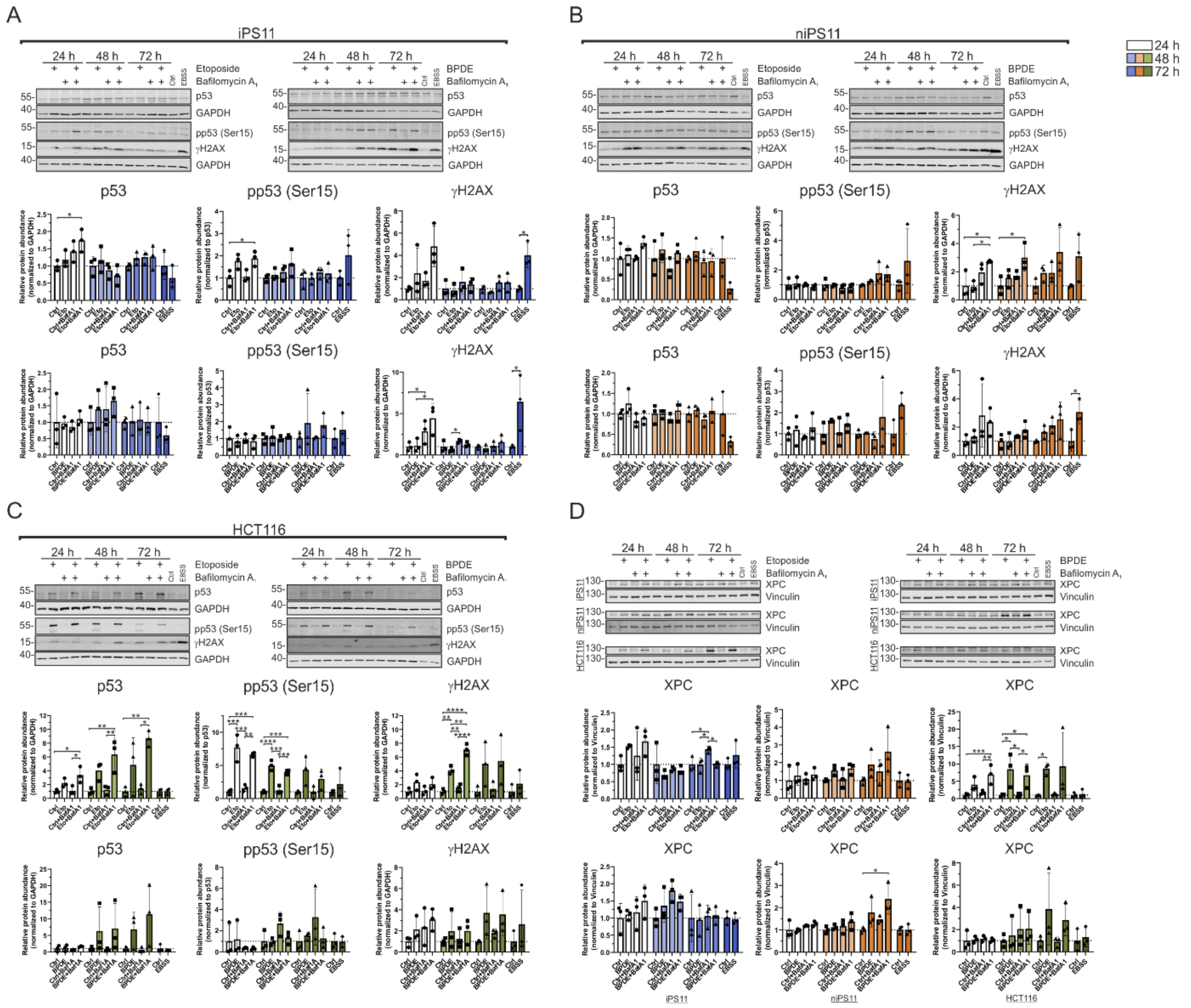

Figure S3: Stem cells show a more moderate impact on DNA damage markers compared to HCT116. (**A-D**) General DNA damage response proteins were investigated by immunoblotting. (**A**) iPS11, (**B**) niPS11 and (**C**) HCT116 were treated with corresponding IC_{20} dose for 24, 48 or 72 h. Therefore, etoposide was supplemented for 24 h and exchanged with genotoxin-free medium for 48 h and 72 h while BPDE was supplemented daily. 4 hours before harvesting medium was exchanged and 40 nM bafilomycin A_1 was supplemented to labeled samples 2 h before lysis. Cells were lysed, and cellular lysates were immunoblotted for p53, phospho-p53 Ser15, phospho-H2AX Ser139 (γH2AX) and GAPDH. (**D**) NER protein XPC was immunoblotted as described above. One representative immunoblot is shown. Results show mean + SD of three independent experiments. For statistical analysis, ordinary

one-way ANOVA (Tukey´s multiple comparisons test; for kinetic analysis) and Student´s t-test (for control/EBSS) were utilized. * $p < 0.05$, ** $p < 0.01$, *** $p < 0.001$, **** $p < 0.0001$.

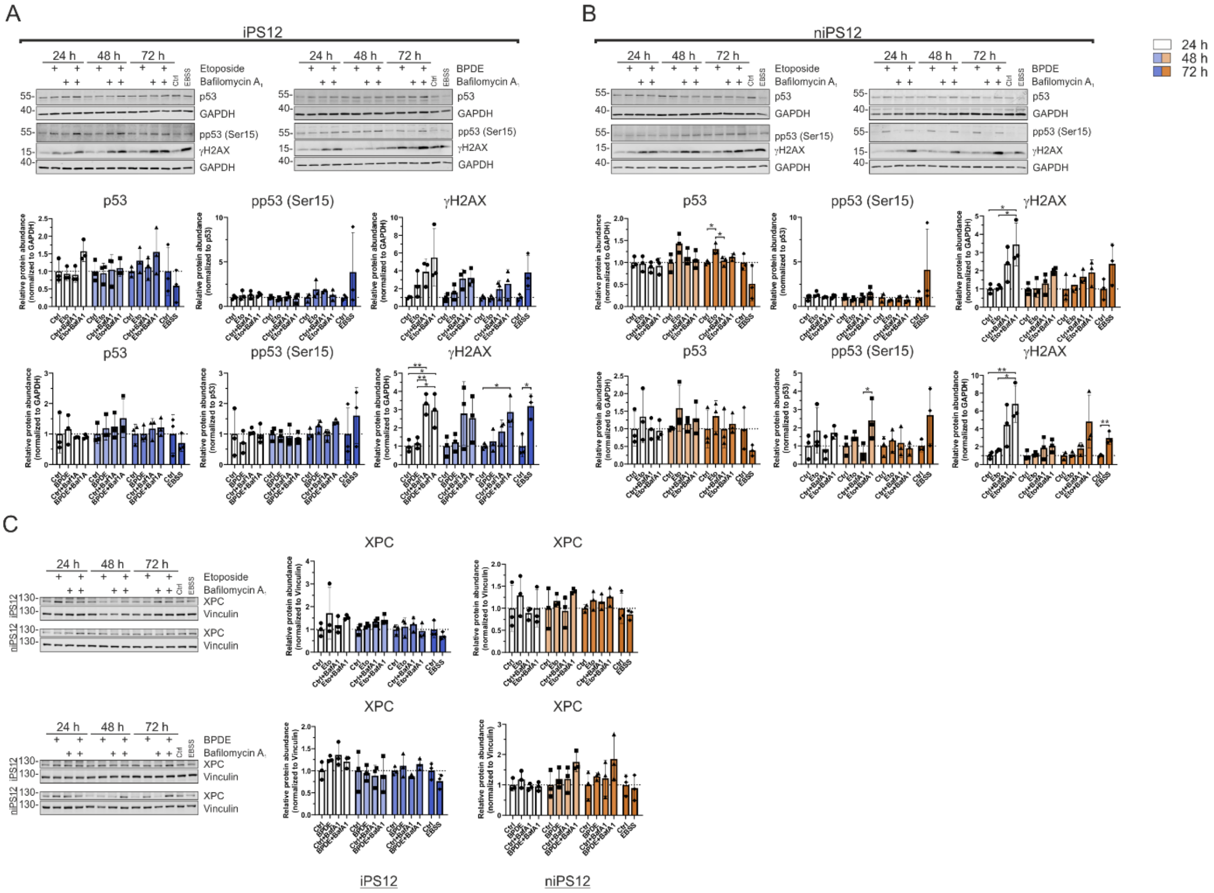

Figure S4: iPS12/niPS12 show no significant effect on p53 but an increase of γH2AX after genotoxic treatment. (**A-C**) General DNA damage response proteins were investigated by immunoblotting. (**A**) iPS12 and (**B**) niPS12 were treated with corresponding IC_{20} dose for 24, 48 or 72 h. Therefore, etoposide was supplemented for 24 h and exchanged with genotoxin-free medium for 48 h or 72 h while BPDE was supplemented daily. 4 hours before harvesting medium was exchanged and 40 nM bafilomycin A_1 was supplemented to labeled samples 2 h before lysis. Cells were lysed, and cellular lysates were immunoblotted for p53, phospho-p53 Ser15, phospho-H2AX Ser139 (γH2AX) and GAPDH. (**C**) NER protein XPC was immunoblotted as described above. One representative immunoblot is shown. Results show mean + SD of three independent experiments. For statistical analysis, ordinary one-way ANOVA (Tukey´s multiple comparisons test; for kinetic analysis) and Student´s t-test (for control/EBSS) were utilized. * $p < 0.05$, ** $p < 0.01$, *** $p < 0.001$, **** $p < 0.0001$.

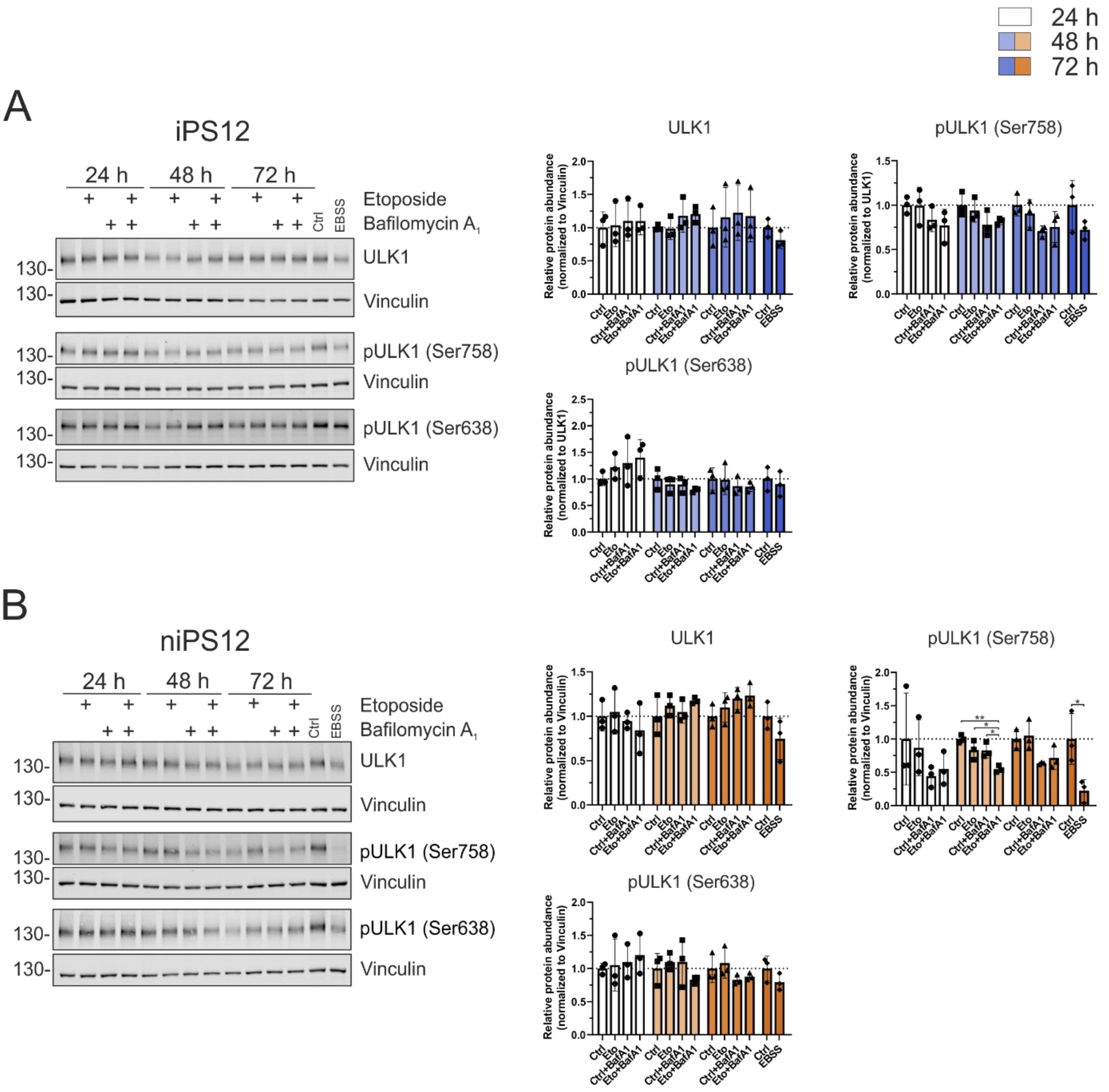

Figure S5: ULK1 activation status is not affected in iPS12 after etoposide treatment. (**A**) iPS12 and (**B**) niPS12 were treated with corresponding IC_{20} dose of etoposide and lysed after 24, 48 or 72 h. Cellular lysates were immunoblotted for ULK1, phospho-ULK1 Ser758 and phospho-ULK1 Ser638 respectively. One representative immunoblot is shown. Results show mean + SD of three independent experiments. For statistical analysis, ordinary one-way ANOVA (Tukey´s multiple comparisons test; for kinetic analysis) and Student´s t-test (for control/EBSS) were utilized. * p < 0.05, ** p < 0.01, *** p < 0.001, **** p < 0.0001.

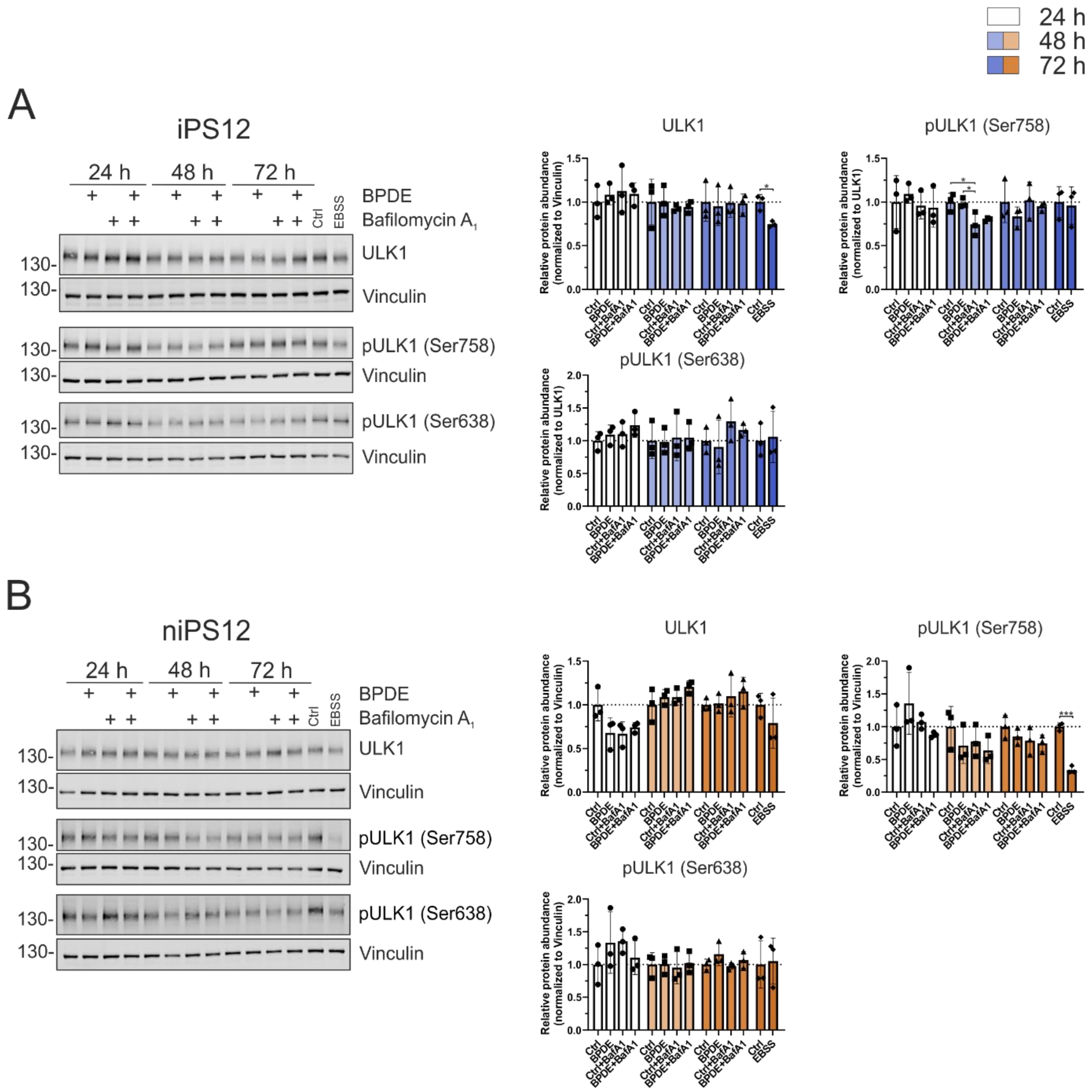

Figure S6: BPDE exposure does not influence ULK1 phosphorylation. (**A**) iPS12 and (**B**) niPS12 were treated with corresponding IC_{20} dose of BPDE and lysed after 24, 48 or 72 h. Cellular lysates were immunoblotted for ULK1, phospho-ULK1 Ser758, phospho-ULK1 Ser638, or vinculin. One representative immunoblot is shown. Results show mean + SD of three independent experiments. For statistical analysis, ordinary one-way ANOVA (Tukey´s multiple comparisons test; for kinetic analyis) and Student´s t-test (for control/EBSS) were utilized. * $p < 0.05$, ** $p < 0.01$, *** $p < 0.001$, **** $p < 0.0001$.

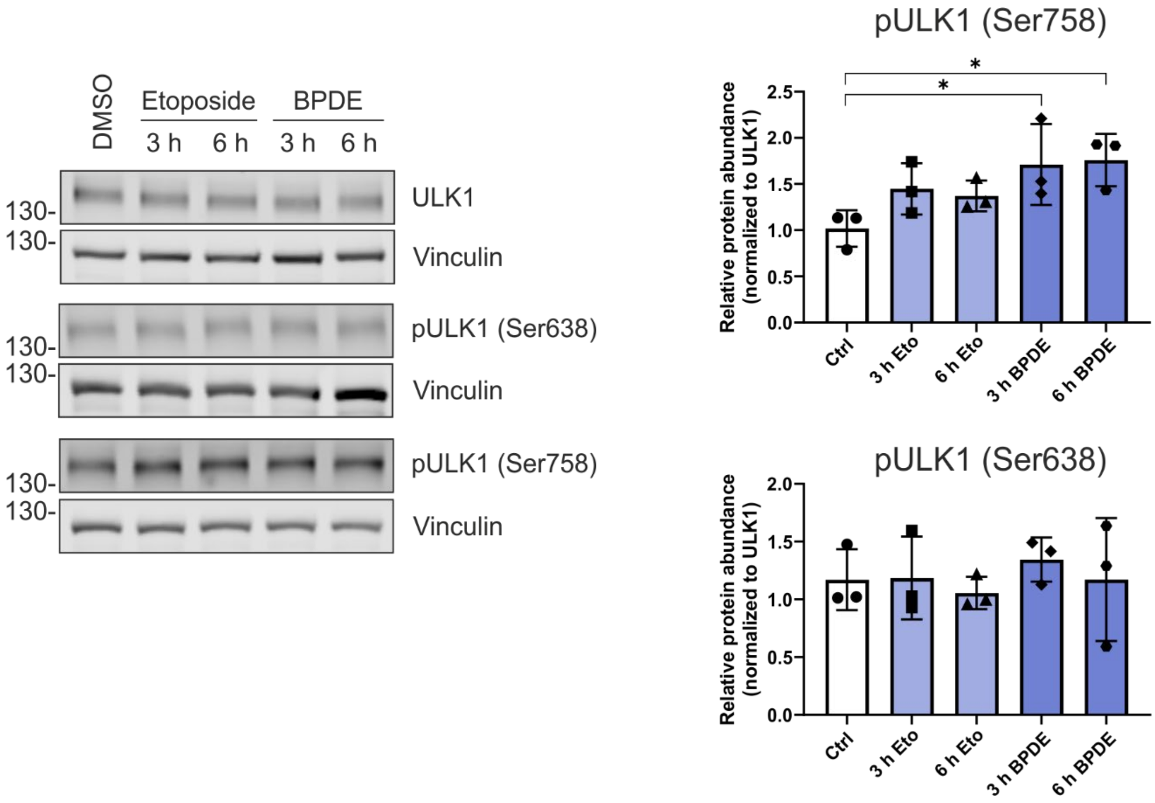

Figure S7: Etoposide or BPDE treatment does not affect ULK1 phosphorylation after 3 or 6 hours. iPS11 were treated with corresponding IC_{20} doses of etoposide or BPDE, respectively, and lysed after 3 or 6 h. Cellular lysates were immunoblotted for ULK1, phospho-ULK1 Ser758, phospho-ULK1 Ser638, or vinculin. One representative immunoblot is shown. Results show mean + SD of three independent experiments. For statistical analysis, ordinary one-way ANOVA (Tukey´s multiple comparisons test) was utilized. * $p < 0.05$, ** $p < 0.01$, *** $p < 0.001$, **** $p < 0.0001$.

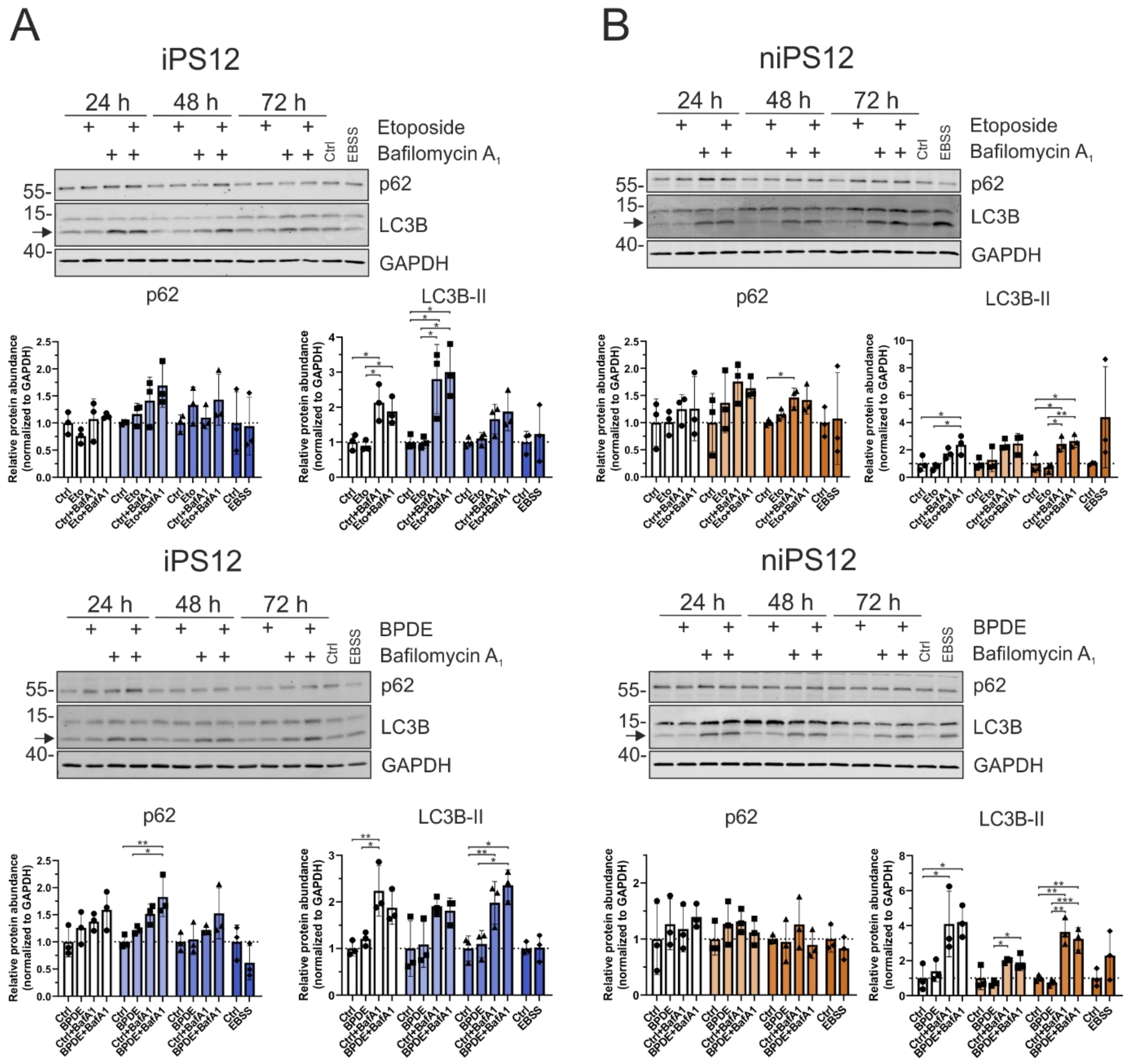

Figure S8: Autophagic flux is not affected by genotoxic treatment. (**A**) iPS12 and (**B**) niPS12 were treated with corresponding IC_{20} dose for 24, 48 or 72 h. Cells were lysed, and cellular lysates were immunoblotted for SQSTM1/p62, LC3B and GAPDH. One representative immunoblot is shown. Results show mean + SD of three independent experiments. For statistical analysis, ordinary one-way ANOVA (Tukey´s multiple comparisons test; for kinetic analysis) and Student´s t-test (for control/EBSS) were utilized. * $p < 0.05$, ** $p < 0.01$, *** $p < 0.001$, **** $p < 0.0001$.

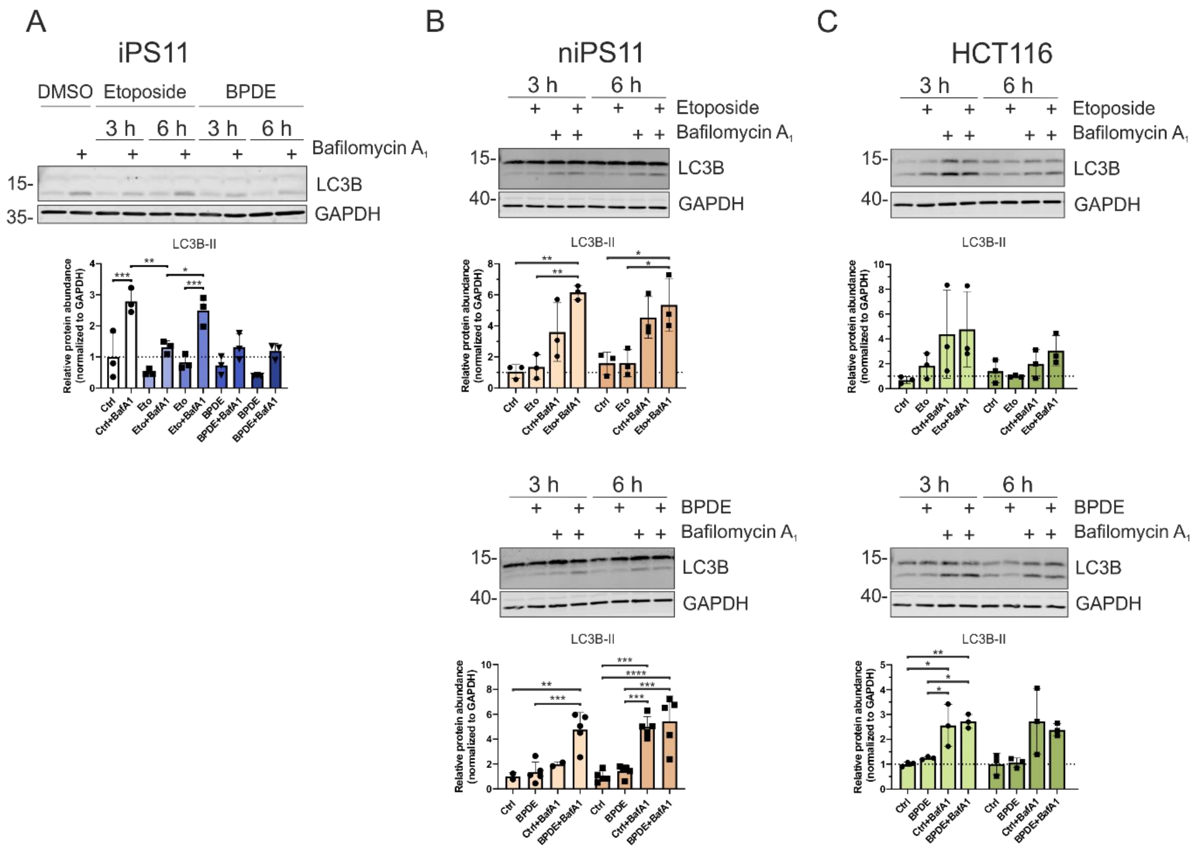

Figure S9: LC3 turnover is not affected by genotoxic treatment after 3 or 6 hours. (**A**) iPS11, (**B**) niPS11, and (C) HCT116 cells were treated with corresponding IC_{20} doses for 3 or 6 h. Cells were lysed, and cellular lysates were immunoblotted for LC3B and GAPDH. One representative immunoblot is shown. Results show mean + SD of three independent experiments. For statistical analysis, ordinary one-way ANOVA (Tukey´s multiple comparisons test) were utilized. * p < 0.05, ** p < 0.01, *** p < 0.001, **** p < 0.0001.

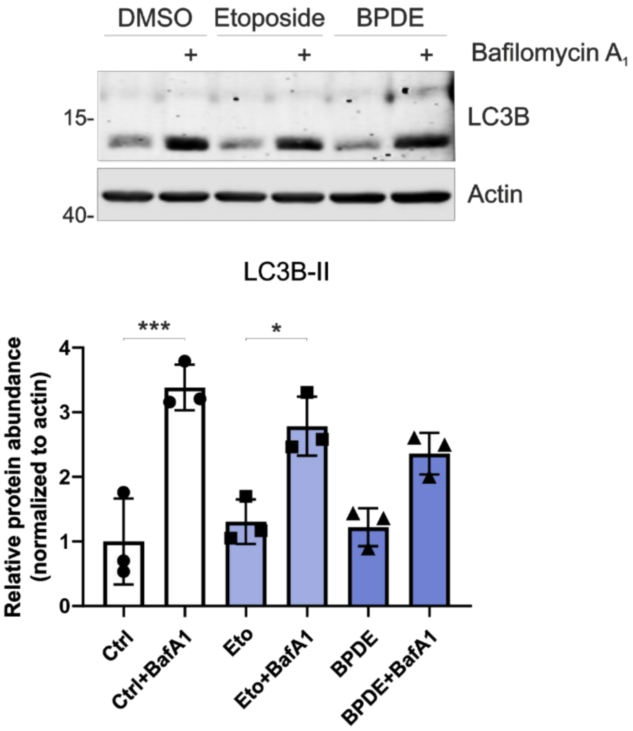

Figure S10: LC3 turnover is not affected by treatment with IC_{50}s of genotoxins. iPS11 were treated with corresponding IC_{50} doses for 24 h. Cells were lysed, and cellular lysates were immunoblotted for LC3B and actin. One representative immunoblot is shown. Results show mean + SD of three independent experiments. For statistical analysis, ordinary one-way ANOVA (Tukey´s multiple comparisons test) were utilized. * $p < 0.05$, ** $p < 0.01$, *** $p < 0.001$, **** $p < 0.0001$.

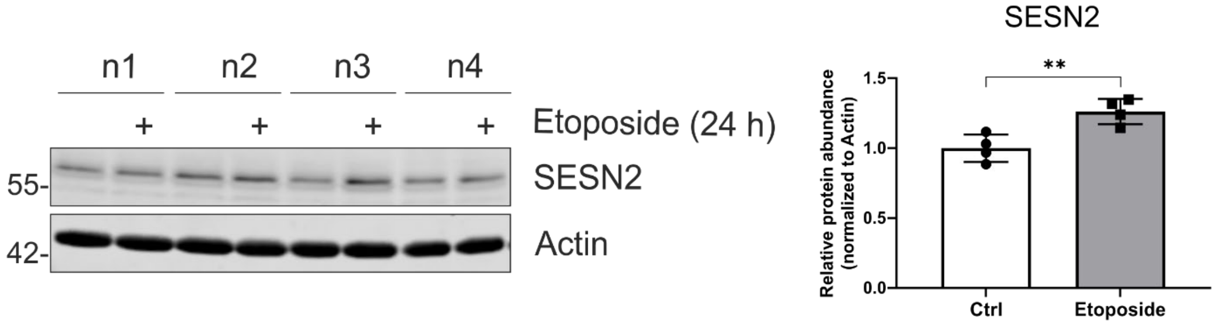

Figure S11: SESN2 protein abundance is slightly upregulated upon etoposide treatment in niPS11. niPS11 were either treated with etoposide for 24 h afterwards lysed. Cellular lysates were immunoblotted for SESN2 and actin as loading control. The blot with all replicates is shown. Results show mean + SD of four independent experiments. Statistical analysis was performed using unpaired two-tailed Student's t-test with Welch's correction to compare genotoxin-treated samples with DMSO controls. ** $p < 0.01$.

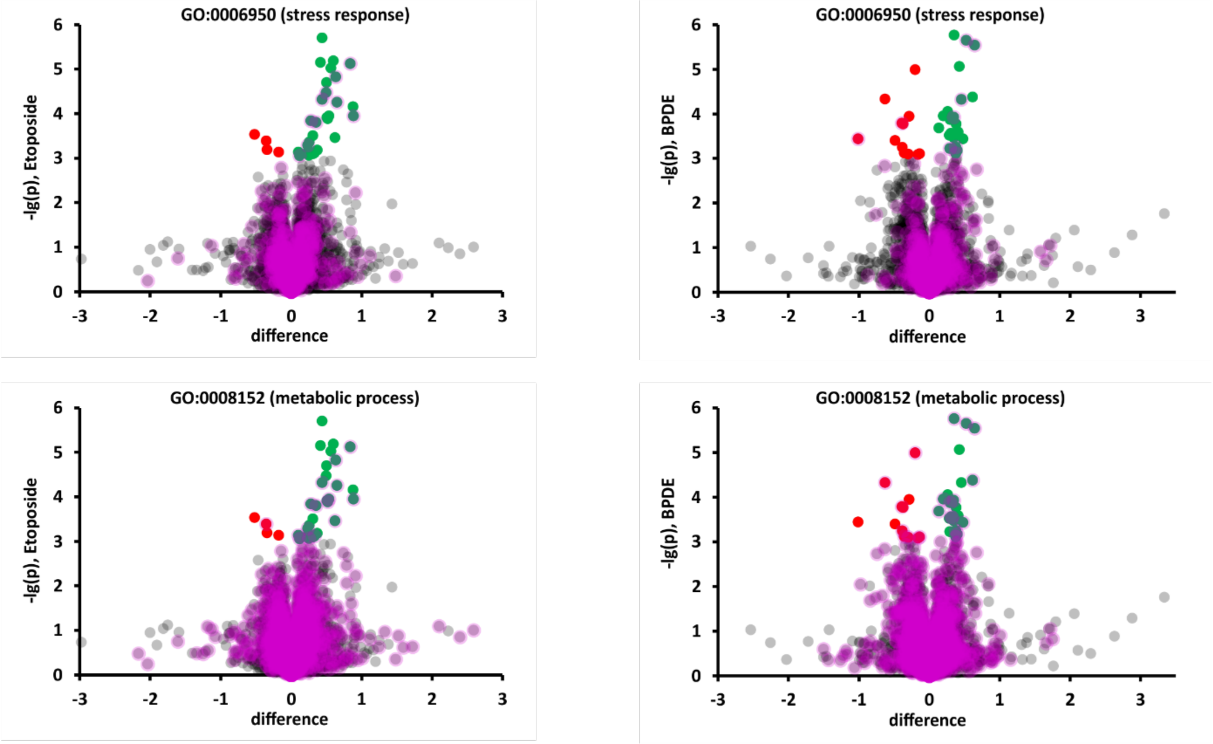

Figure S12: Differential Proteome Analysis of BPDE- and etoposide-treated niPS11 with regard to proteins involved in stress response or metabolic process. Volcano plots based on intensity values for MS-based proteomics of BPDE- or etoposide-treated niPS11 as shown in Figure 7C. Proteins linked to stress response (GO:0006950 from String 12.0 database) or metabolic processes (GO:0008152 from String 12.0 database) are indicated by purple shading of data points. Proteins with a -lg(p-value) >= 3 significance cutoff are displayed as red (down-regulated) or green (up-regulated) data points.

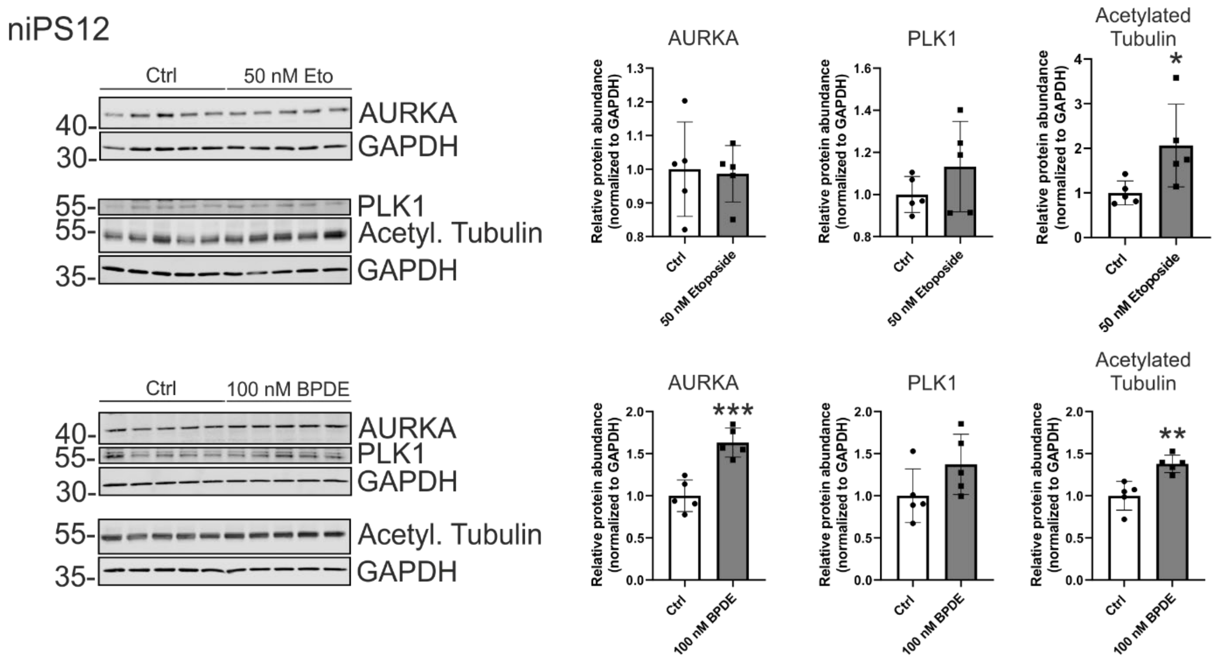

Figure S13: Mitosis-related proteins are affected upon genotoxic treatment in niPS12. niPS12 were either treated with etoposide for 24 h or BPDE for 48 h and afterwards lysed. Cellular lysates were immunoblotted for Aurora kinase A (AURKA), polo like kinase 1 (PLK1), acetylated tubulin (K40), and GAPDH as loading control. The blot with all samples is shown. Results show mean + SD of five independent experiments. For statistical analysis, Student´s t-test was utilized to compare means of genotoxin-treated samples to DMSO. * p < 0.05, ** p < 0.01, *** p < 0.001, **** p < 0.0001.

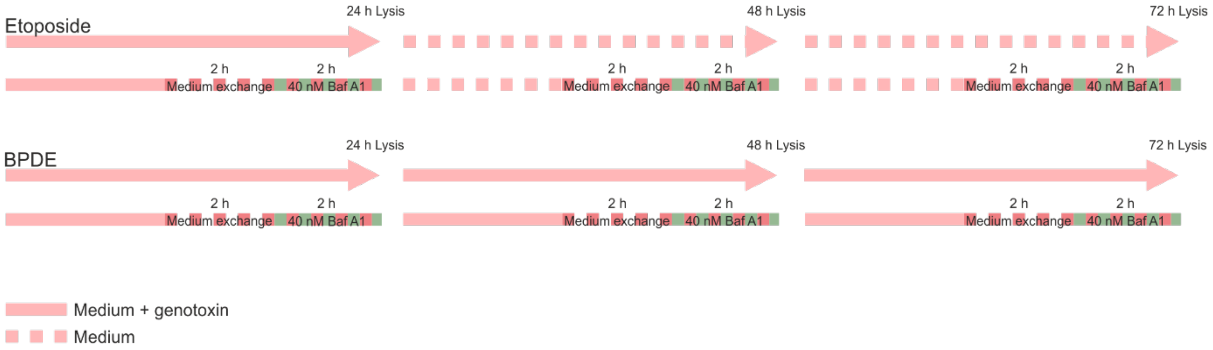

Figure S14: Treatment scheme used in this study.

The medium was exchanged every 24 h to exclude starvation-induced autophagy. On the day of lysis, medium was exchanged 4 h and 40 nM bafilomycin A_1 was supplemented 2 h prior to lysis. Cells treated with etoposide were incubated with the genotoxin for 24 h (solid line). In following medium exchanges etoposide was not included (dotted line). In case of BPDE treatment, BPDE was supplemented daily to the cells corresponding to the IC_{20} value, and cells were processed as described before on the day of lysis.
